# The impact of genomic variation on protein phosphorylation states and regulatory networks

**DOI:** 10.15252/msb.202110712

**Published:** 2022-05-16

**Authors:** Jan Grossbach, Ludovic Gillet, Mathieu Clément‐Ziza, Corinna L Schmalohr, Olga T Schubert, Maximilian Schütter, Julia S P Mawer, Christopher A Barnes, Isabell Bludau, Matthias Weith, Peter Tessarz, Martin Graef, Ruedi Aebersold, Andreas Beyer

**Affiliations:** ^1^ Excellence Cluster on Cellular Stress Responses in Aging Associated Diseases University of Cologne Cologne Germany; ^2^ Department of Biology Institute of Molecular Systems Biology ETH Zurich Zurich Switzerland; ^3^ Center for Molecular Medicine Cologne (CMMC) Medical Faculty, University of Cologne Cologne Germany; ^4^ Lesaffre International Marcq‐en‐Barœul France; ^5^ Department of Human Genetics University of California, Los Angeles Los Angeles CA USA; ^6^ Max Planck Institute for Biology of Ageing Cologne Germany; ^7^ Novo Nordisk Research Center Seattle, Inc. Seattle WA USA; ^8^ Department of Proteomics and Signal Transduction Max Planck Institute of Biochemistry Martinsried Germany; ^9^ Faculty of Science University of Zurich Zurich Switzerland; ^10^ Institute for Genetics Faculty of Mathematics and Natural Sciences University of Cologne Cologne Germany

**Keywords:** budding yeast, multi‐omics genetic effects, phosphorylation, QTL, systems genetics, Genetics, Gene Therapy & Genetic Disease, Post-translational Modifications & Proteolysis, Proteomics

## Abstract

Genomic variation impacts on cellular networks by affecting the abundance (e.g., protein levels) and the functional states (e.g., protein phosphorylation) of their components. Previous work has focused on the former, while in this context, the functional states of proteins have largely remained neglected. Here, we generated high‐quality transcriptome, proteome, and phosphoproteome data for a panel of 112 genomically well‐defined yeast strains. Genetic effects on transcripts were generally transmitted to the protein layer, but specific gene groups, such as ribosomal proteins, showed diverging effects on protein levels compared with RNA levels. Phosphorylation states proved crucial to unravel genetic effects on signaling networks. Correspondingly, genetic variants that cause phosphorylation changes were mostly different from those causing abundance changes in the respective proteins. Underscoring their relevance for cell physiology, phosphorylation traits were more strongly correlated with cell physiological traits such as chemical compound resistance or cell morphology, compared with transcript or protein abundance. This study demonstrates how molecular networks mediate the effects of genomic variants to cellular traits and highlights the particular importance of protein phosphorylation.

## Graphical abstract

A systematic analysis of how genetic variation in a yeast cross affects the transcriptome, the proteome and the phosphoproteome reveals multi‐layered changes in regulatory networks that ultimately have an impact on hundreds of cellular traits.

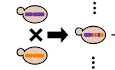

## Introduction

Genetic polymorphisms are important modifiers of many physiological traits, such as body height or disease susceptibility. Differences in these traits are caused by alterations in the underlying molecular regulatory networks (Emilsson *et al*, 2008). The signal processing in regulatory networks is determined by both the concentrations of relevant molecules and their state or activity. The abundance of molecules is altered by biosynthetic or degradative processes, such as transcription and translation, whereas their biological activity is determined by their location, protein folding, or post‐translational modifications (among others). For instance, the net activity of a protein can be modulated not only by changing its abundance but also by changing its state, e.g., by increasing or decreasing regulatory phosphorylation. The regulatory effect of phosphorylation can be realized not only via direct activation or inhibition (Ardito *et al*, 2017) but also via modulation of the degradation rate (Henchoz *et al*, 1997), changes in subcellular localization (Miller & Cross, 2001), or complex association (Abdollah *et al*, 1997). Regulatory phosphorylation in turn affects the biosynthesis of yet other gene products, e.g., through modulating transcription and translation. Thus, there is a great diversity of as yet poorly defined mechanisms of crosstalk between biosynthesis and molecular signaling networks (van der Sijde *et al*, 2014).

High‐throughput molecular profiling (“omics”) technologies have enabled the characterization and quantification of individual molecular layers such as the transcriptome, proteome, and metabolome in large populations. Such data have been used to identify genetic variants that explain some of the variation in the measured traits, termed quantitative trait loci (QTLs). For example, it is possible to detect expression QTLs (eQTLs) for virtually all transcripts present in a yeast cell (Albert *et al*, 2018). Likewise, mass spectrometry has been used to identify QTLs for hundreds of proteins (pQTLs) (Fu *et al*, 2009; Foss *et al*, 2011; Holdt *et al*, 2013; Picotti *et al*, 2013; Wu *et al*, 2013; Okada *et al*, 2016; Singh *et al*, 2016; Keele *et al*, 2021) and metabolic traits (mQTLs; reviewed in Gowda & Djukovic, 2014). Despite technological progress in multi‐layer molecular analyses, existing studies have focused on effects of genomic variants on molecular abundance, whereas the impact on protein states (and thus regulatory network changes) has not been systematically explored. There is only anecdotal evidence about how specific genetic variants influence regulatory pathways with downstream effects on RNA levels (Smith & Kruglyak, 2008). Post‐translational modifications (PTMs) such as phosphorylation have been largely neglected in this context, and no systematic phosphorylation QTL (phQTL) studies have been performed to date. Consequently, we lack a detailed understanding of genetic effects on regulatory networks and important questions remain unanswered: Are genomic variants that affect protein states located primarily in coding or non‐coding regions? To what extent is protein abundance determined by the abundance of the respective transcript compared with post‐transcriptional regulation? To what extent do genomic variants affecting cellular traits mediate their effects through molecular state changes as opposed to through abundance changes?

**Figure 1:**
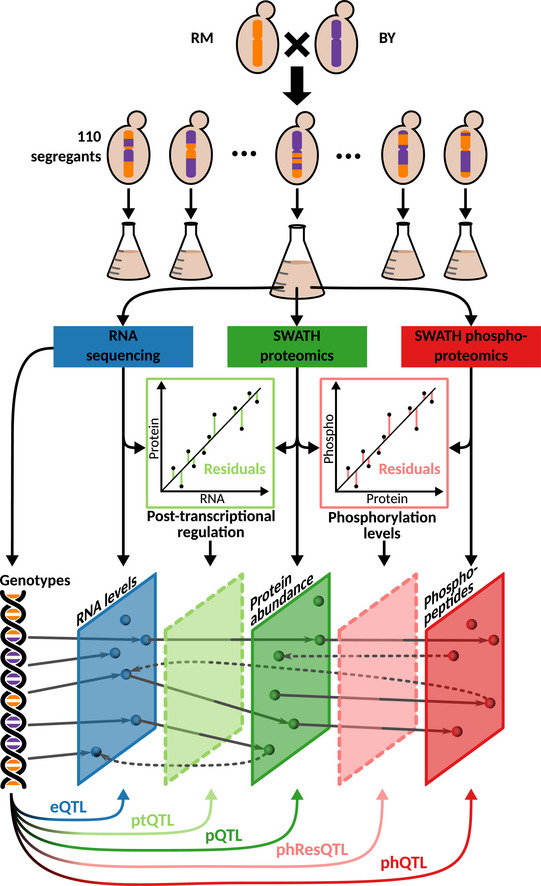
Experimental design and study overview Yeast segregants derived from two parental strains, BY and RM, were grown and characterized with different omics approaches. Their transcriptome, proteome, and phosphoproteome were directly quantified in samples obtained from the same culture flask. Residual traits representing the disparities between transcript and protein levels (i.e., measuring post‐transcriptional regulation, light green), and those between protein and phosphopeptide levels (i.e., measuring phosphorylation status, pink) were computed. QTL analyses were performed to determine the effect of genetic variation on each of these molecular layers.

To answer these questions, we designed a multi‐omics QTL study in recombinant offspring of a cross of two budding yeast strains. The study has four key distinguishing features: (i) We quantified transcripts, proteins, and protein phosphorylation levels at a genomic scale; (ii) by using the SWATH‐mass spectrometry (SWATH‐MS) technology, we could reproducibly quantify a large number of proteins across virtually all samples; (iii) the analyzed RNA, protein, and phosphoprotein samples were obtained from the same yeast cultures to minimize experimental variability and to accommodate for the complex relationship between abundance and regulatory modifications (Civelek & Lusis, 2014); and (iv) the data integration scheme that we developed for this study enabled us to investigate the crosstalk between biosynthetic processes and regulatory network changes. This setup, illustrated in Fig 1, enabled us, for the first time, to map the response of cellular signaling networks to genomic variation, to dissect the interaction of transcript and protein abundance changes with protein phosphorylation states, and to investigate the relevance of phQTLs for complex cellular traits.

## Results

### Multi‐omics profiling of a yeast panel

**Figure 2:**
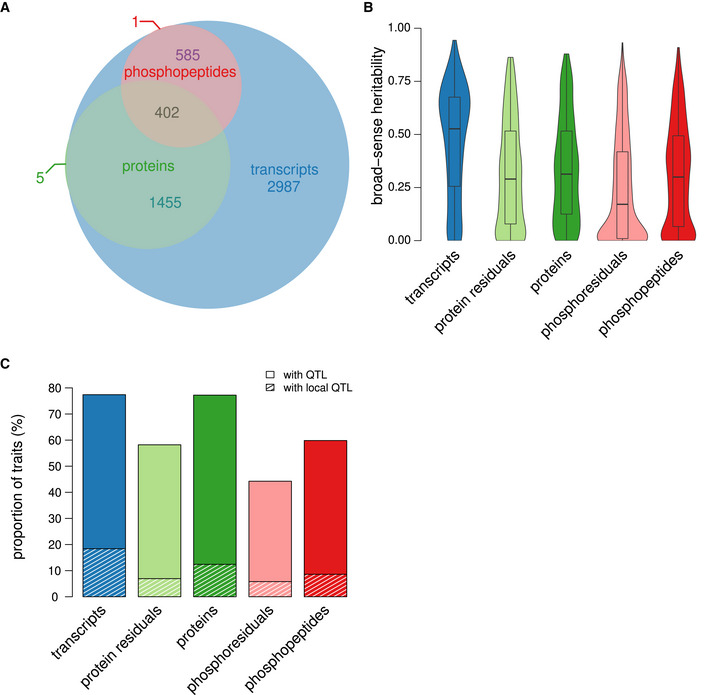
Genetic control of molecular phenotypes Overlap of the quantified transcriptome, proteome, and phosphoproteome. The numbers refer to the amount of genes for which a product could be measured on the respective layer.Broad‐sense heritability of traits belonging to the 402 genes for which measurements at each molecular level were available (*n* = 402 for the first three boxes from the left corresponding to trait levels by gene; *n* = 879 for the two boxes on the right corresponding to individual phosphopeptides belonging to the same 402 genes). The boxes extend from the first quartile to the third quartile of the data. The median is represented by the central line. Whiskers extend up to the most extreme data point within a distance of 1.5 times the interquartile range relative to the closest border of the box.Proportions of molecular traits affected by at least one QTL are shown separately for each molecular layer. Shaded areas indicate the proportion of traits for which a local QTL was found.

The BYxRM *Saccharomyces cerevisiae* segregant panel used in this study results from a cross between a laboratory strain (BY4716) isogenic to the reference strain S288C, and a derivative of a wild isolate from a vineyard (RM11‐1a). This cross was previously used to study the genetic contribution to molecular traits including RNA and protein levels and to test novel systems genetics approaches (Brem *et al*, 2002; Foss *et al*, 2007; Albert *et al*, 2014). We grew the two parental haploid yeast strains and 110 of their recombinant offspring under tightly controlled conditions with multiple replicates for some of the strains (Fig 1, Dataset EV3). The transcriptomes of 150 cultures were sequenced at high coverage (38‐186x) allowing for the quantification of 5,429 transcripts in all samples (Dataset EV1). The RNA‐seq data were also used to infer the genotypes of the strains (Clément‐Ziza *et al*, 2014) (Appendix Fig S1, Dataset EV2), which increased the number of unique markers compared with array‐based genotyping (3,593 unique markers compared with 2,957 markers from Gillet *et al*, 2012). While the resolution of the QTL mapping is mainly limited by the number of segregants (i.e., recombinations), this procedure allows for more accurate genotyping. In samples obtained from the same yeast cultures, we used SWATH‐MS (Gillet *et al*, 2012; Selevsek *et al*, 2015) to measure the abundance of 1,862 proteins with < 1.8% missing values across all samples (Fig 2A, Dataset EV1). This represents a fourfold increase in the number of consistently quantified proteins compared with previous studies in the same cross (Foss *et al*, 2007, 2011; Picotti *et al*, 2013; Albert *et al*, 2014). We also quantified the phosphorylation state of the proteins by SWATH‐MS. After stringent filtering, we obtained the abundance of 2,116 phosphopeptides from 988 proteins with < 2.4% missing values across all samples (Dataset EV1). These peptides correspond to a total of 3,716 phosphorylated residues, including serines (2,832, 76.2%), threonines (825, 22.2%), and tyrosines (59, 1.6%). Only peptides that were not polymorphic between the parental strains were considered here.

We observed that in most cases, protein abundance was positively correlated with the corresponding transcript level (average *r* = 0.23), and the abundance of most phosphopeptides was positively correlated with the corresponding protein (average *r* = 0.29) (Appendix Fig S2). However, each layer can also be affected independently of the genetic effects on the other layers. In order to identify such direct effects, we generated computationally derived traits, from which the effects acting on other molecular layers were removed (Foss *et al*, 2011) (termed residual traits). First, we estimated the contribution of transcript to protein‐level changes by regressing the concentration of a given protein against the concentration of its encoding transcript across all strains (schematic representation in Fig 1). Deviations from this regression (residuals) can either result from noise in the data or from pQTL effects that are independent of transcript‐level changes. We used the residuals as estimates of post‐transcriptional regulation, resulting in 1,857 post‐transcriptional (pt) traits (Foss *et al*, 2011). Likewise, we regressed phosphopeptide levels against levels of the proteins of origin and used the residuals as estimates of differential phosphorylation, resulting in 879 phospho‐residual (phRes) traits. The QTLs obtained by mapping these residual traits were termed post‐transcriptional QTL (ptQTL) and phospho‐residual QTL (phResQTL), respectively.

### QTLs have widespread effects on all quantified molecular layers

In order to quantify the fraction of trait variation that can be attributed to genetic differences, we quantified broad‐sense heritability by leveraging available replicates as proposed before (Bloom *et al*, 2013) (Fig 2B, Appendix Fig S3).

In short, broad‐sense heritability was estimated by comparing the variation between replicates from the same strain (which is non‐genetic) and the total variation between strains. When the intra‐strain variation is small compared with the inter‐strain variation, it can be concluded that the genetic contribution to trait variation is large under the experimental conditions tested (Bloom *et al*, 2013). All five types of molecular traits outlined above had heritability greater than expected by chance (all *P* < 2.2E‐16, Wilcoxon rank‐sum test). The fact that 525 (28%) pt traits and 165 (18%) phRes traits had heritability greater than 50% indicates that at least part of the residual variation is genetically determined and not just technical and/or biological noise (Foss *et al*, 2011). Furthermore, while transcripts and protein abundance tend to reach greater heritability on average, there are many genes with greater heritability on the phospho‐layer compared with their transcript and protein abundance (Appendix Fig S3). This observation is suggesting direct genetic effects on phosphopeptides that are not (solely) mediated via protein abundance changes.

**Figure EV1:**
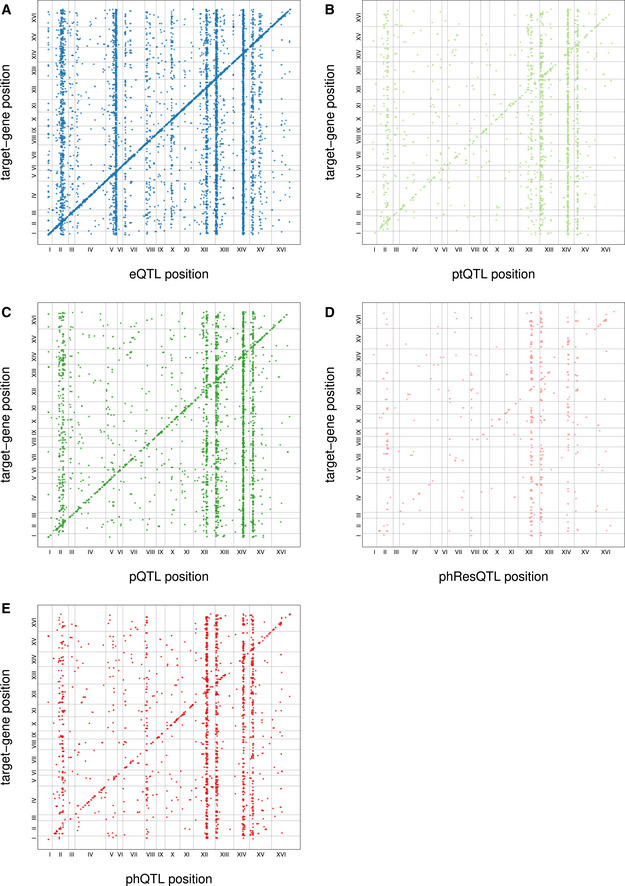
Significant QTL at FDR < 10% A–EAssociations between QTL and targets are shown as dots with X‐coordinates showing the position of the QTL and Y‐coordinates showing the position of the affected trait in the genome. Vertical bars indicate QTL hotspots while the diagonal consists of local QTL. Shown are eQTL (A), ptQTL (B), pQTL (C), phResQTL (D), and phQTL (E).

We utilized a Random Forest‐based mapping strategy to identify QTLs (Michaelson *et al*, 2010), resulting in the detection of 5,776 eQTLs, 2,078 pQTLs, and 1,327 ptQTLs at a false discovery rate (FDR) below 10% (Dataset [Link], [Link], Fig EV1A–E). The same fraction of transcripts and proteins had at least one QTL (77% at FDR<10% in both cases; Fig 2C). This large proportion of proteins with at least one pQTL underlines the high quality of the proteomics data (Foss *et al*, 2011). We also detected 1,595 phQTLs affecting 1,266 phosphopeptides (60%) and 466 phResQTLs affecting 389 phospho‐residuals (44%) (Dataset [Link], [Link]). The physiological importance (growth effects) of 76 of the phosphopeptides that we quantified had previously been analyzed using phospho‐deficient mutants (Viéitez *et al*, 2022). Based on that data, we estimated that at least 39 of these phosphopeptides were functionally relevant. Others might still have an effect in growth conditions that were not studied by Viéitez *et al*. Functional phosphopeptides were similarly likely to be affected by a phQTL (20 out of 39) compared with non‐functional phosphopeptides (23 out of 37). Thus, genetic variation in the BYxRM cross impacts on physiologically relevant phosphorylation traits to an extent that is similar to peptides for which no functional relevance was established yet.

To understand where these QTLs are located with respect to their target genes, we classified QTLs as either “local” or “distant”, based on their linkage disequilibrium with the genetic marker that is closest to the affected gene. QTLs were considered local if they had a correlation of *r* > 0.8 with the allele of the affected gene. Marker pairs fulfilling this criterion had a genetic linkage of 7.9 cM on average. The fraction of molecular traits with a local QTL was in a similar range for all directly measured traits (10–20%) (Fig 2C). The residual‐derived traits (pt and phRes) had the smallest fraction of local QTLs, which may be due to biological reasons, increased measurement noise (as discussed above), or a combination of the two.

**Figure 3:**
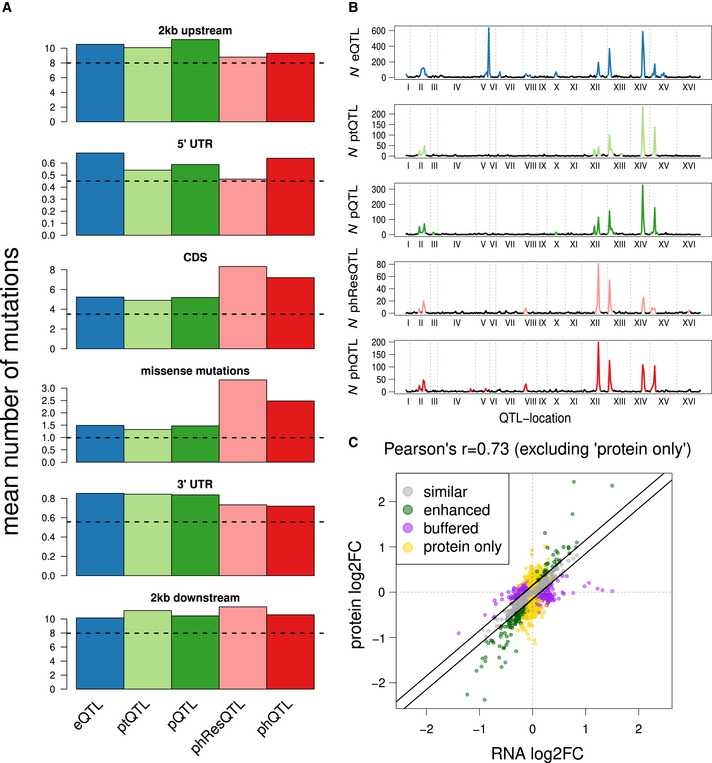
Genomic effects on transcript and protein levels Average number of SNPs in and around genes affected by local QTLs at the respective molecular layer. The horizontal dashed black line shows the average number of SNPs across all annotated genes in the respective regions.Number of traits affected by each locus at FDR < 10% for each molecular layer. Regions with more QTLs than expected by chance (QTL hotspots) are shown in color.Allelic effects of QTLs on transcript compared with their effect on the corresponding protein. Each dot represents an association of a QTL with a gene for all significant eQTLs. The relationship between effect size and direction on the transcript and protein levels is color‐coded based on the four effect classes described in the main text. Axes show the log2‐transformed fold changes of BY versus RM alleles.

Next, we asked whether local eQTLs and pQTLs could be attributed to changes in the sequence of the respective transcript, which might influence transcription and translation rates. When comparing genes with local QTLs to genes that were only affected by distant QTLs, we found that the former had an increased number of polymorphisms in non‐coding regions (e.g., 5′‐untranslated regions (UTRs) or 3′‐UTRs; Fig 3A, Appendix Fig S4). The existence of local ptQTLs (129 local ptQTLs for 6.9% of all pt traits) underlines that protein levels can be influenced independently of their coding transcripts. Notably, targets of local ptQTLs were enriched for polymorphisms outside of coding regions (Fig 3A, Appendix Fig S4), which suggests that variants in non‐coding parts of the genome can influence post‐transcriptional regulation (Foss *et al*, 2011). The underlying mechanisms might be polymorphisms in ribosomal binding sites or upstream open reading frames (uORFs) (Morris & Geballe, 2000) affecting translation initiation or variants altering mRNA processing (e.g., capping and polyadenylation) (Bernstein & Toth, 2012).

Regions in the genome that affect significantly more traits than expected by chance are referred to as QTL hotspots (Smith & Kruglyak, 2008). Since these loci affect large numbers of molecular traits, they often act through master regulators, for instance transcription factors or kinases in yeast (Yvert *et al*, 2003; Albert *et al*, 2018). We detected between 9 and 15 significant hotspots for each molecular layer, with the eQTL layer having the largest number of hotspots (Fig 3B, Dataset [Link], [Link]). Many of the detected hotspots have been reported as eQTL hotspots for this yeast cross before, and a causal gene has been validated for some of them, e.g., *HAP1* (chrXII:2, a transcription factor), *IRA2* (chrXV:1, an inhibitor of RAS signaling), and *MKT1* (chrXIV:1, a post‐transcriptional regulator and interactor of Pab1p)(Brem *et al*, 2002; Smith & Kruglyak, 2008). While we observed hotspots that predominantly impacted the transcriptome (e.g., chrV:2), the proteome (e.g., chrXII:1), or the phosphoproteome (e.g., chrVIII:1), most hotspots affected multiple molecular layers simultaneously. Furthermore, our data extend previous findings that most distant eQTLs act from within hotspots (Albert *et al*, 2018) to all five types of molecular traits considered here (Appendix Fig S5).

### Transmission of genetic effects from the transcriptome to the proteome

The deep coverage of the transcriptome and proteome in this study enabled us to investigate to what extent transcript‐level changes are transmitted to their corresponding proteins. First, we observed that local eQTLs and local pQTLs of the same genes significantly overlapped (*P* < 2.2E‐16, OR = 11, one‐sided Fisher's exact test), which is expected in the absence of major post‐transcriptional or post‐translational regulation. In order to estimate QTL effect sizes, we split the population of yeast segregants based on the alleles at a linked locus and computed the log fold change of the transcript and protein levels between the two subpopulations. When comparing transcript fold changes with the respective protein fold changes of the same genes, we found that they were strongly correlated (eQTLs with FDR < 10%, *r* = 0.73; Fig 3C). Previous work suggested that local and distant eQTLs affect the proteome in different ways (Foss *et al*, 2011; Chick *et al*, 2016) (Appendix Text S1). Using our data, a regression of transcript and protein fold changes had a slope close to 1 for both local and distant eQTLs (Appendix Fig S6), implying that changes in transcript levels tend to cause similar changes in protein levels of the same genes regardless of the eQTL being local or distant. In addition, for most but not all eQTL hotspots the ensemble of transcript variation was propagated to the protein level, exemplified by the *HAP1* locus (chrXII:2). Effects of this hotspot on protein concentrations were highly correlated with those on the transcripts of the same genes (Pearson’s correlation coefficient *r* = 0.94 for the 289 eQTLs at FDR < 10%).

Despite widespread concordance between eQTL effects on transcripts and proteins, we also detected many eQTLs that exhibited effects on the protein level deviating from those on the transcript level. We classified targets of eQTLs into three groups based on the difference in the QTL effects at the transcript and protein levels (Fig 3C). The first group contained genes for which the effects of an eQTL on the transcript and protein levels of its target gene were similar (“similar”). The second group contained genes for which the effects of an eQTL were repressed or even entirely buffered on the protein level (“buffered”). The third group contained genes for which proteins showed enhanced responses compared with their corresponding transcript (“enhanced”). As a fourth group, we added genes that were affected on their protein level by ptQTLs, without a significant effect on the corresponding transcript level (“protein only”). Gene Ontology (GO) enrichment revealed that genes with eQTLs in the “similar” group were enriched for genes related to protein import into the mitochondrial matrix (Dataset [Link], [Link]). Genes with “buffered” eQTL effects were strongly enriched for terms related to cytoplasmic translation, including the large subunit of the ribosome, and proteins localizing to the nucleolus (Dataset [Link], [Link]). Although buffering of ribosomal proteins has been observed before (Foss *et al*, 2011), it could not be excluded that this was due to technical issues in protein quantification. However, this is unlikely to be the case here: first, because ribosomal proteins are relatively highly expressed and hence easily quantifiable by mass spectrometry; and second, because these proteins were affected by other pQTLs at a similar rate as the rest of the proteome (73% of proteins with buffered eQTLs had at least one pQTL).

Genes affected by the group of “enhanced” eQTLs were strongly enriched for mitochondrial ribosomes and other terms related to mitochondrial translation (Dataset [Link], [Link]). Like buffered proteins, proteins affected by “protein‐only” effects were also enriched for functions related to cytoplasmic translation (Dataset [Link], [Link]). This unexpected functional similarity between buffered proteins and proteins subject to “protein‐only” effects raised the question whether the same proteins could be subject to both. Indeed, we found that genes affected by a buffered eQTL were more likely to also be affected by a “protein‐only” QTL (219 genes; *P* < 7E‐4, Fisher’s exact test). Thus, specific groups of proteins seem to require extensive post‐transcriptional fine‐tuning of their cellular concentrations, decoupling protein levels from transcript levels (Appendix Fig S7).

This notion was further supported by a detailed investigation of individual hotspots with heterogeneous eQTL and pQTL effects. For example, the effects of the *IRA2* hotspot (XV:1) on transcript levels of cytoplasmic ribosomal genes were not transmitted to protein levels, but the same locus affected protein levels of genes related to mitochondrial respiration without changing their transcript levels (Appendix Fig S8). Whereas the effects of the *MKT1* hotspot (XIV:1) on the protein levels of cytosolic ribosomes were buffered, the effects of the same locus on the protein levels of mitochondrial ribosomes were enhanced.

While effects of the same hotspot on the transcript level were transmitted to the protein level for some genes and not for others, we observed a strong agreement for different eQTLs of the same transcript. A pair of eQTLs for the same gene was more likely to be of the same class (“as expected”, “enhanced”, and “buffered”) than a random pair of eQTLs (*P* < 2.2E‐16, OR = 1.89, Fisher's exact test). Thus, our analysis reveals complex post‐transcriptional QTL effects, especially for genes involved in translation, with marked differences between cytoplasmic and mitochondrial translation. The transmission of these effects to the protein level seems to be more dependent on the target gene than the causal variation.

### Phosphorylation states are affected directly by local and distant variants

**Figure 4:**
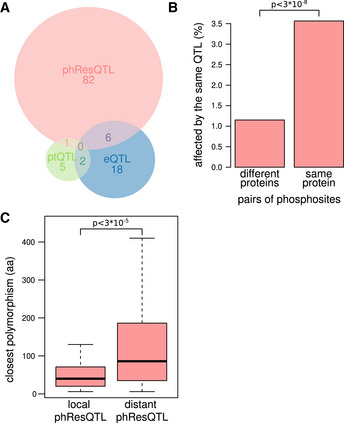
Analysis of phResQTLs Overlap of different QTL types in the *HAP1* locus (chrXII:2) for the 402 genes whose products were detected on the transcript, protein, and phosphopeptide level.Proportion of pairs of phosphosites on different or the same protein that share a phResQTL. The *P*‐value was determined using a one‐sided Fisher's exact test.Distance of phosphosites affected by a local phResQTL or exclusively by distant phResQTLs to the nearest missense mutation in the protein of origin (*n* = 49 and *n* = 131 for the left and right boxes, respectively). The *P*‐value was determined using a Wilcoxon rank‐sum test. The boxes extend from the first quartile to the third quartile of the data. The median is represented by the central line. Whiskers extend up to the most extreme data point within a distance of 1.5 times the interquartile range relative to the closest border of the box. Outliers (points more extreme than the whiskers) are not shown.

To investigate effects acting directly on the phosphorylation state of proteins (i.e., not through protein abundance changes), we focused on the phRes traits, for which the phosphorylation effects were corrected for abundance changes of the proteins of origin, as described above. After this correction, we detected 466 phResQTLs affecting 389 phRes traits (44% of all quantifiable phRes traits) and there were multiple phResQTL hotspots (Fig 3B). A striking example of a phResQTL hotspot is the *HAP1* locus (chrXII:2), affecting 22.1% of phosphoproteins, but only 6.5% and 5.2% of the transcripts and proteins of the 402 genes whose products were detected on all three molecular layers (Fig 4A). Remarkably, this is despite the molecular function of HAP1 as a transcription factor (Jensen‐Pergakes *et al*, 2001). Thus, this hotspot exemplifies widespread genetic effects on the phospho‐layer, which could not be observed by only studying transcript or protein abundance.

Due to the notion that multiple phosphosites on the same protein are often targeted by a common kinase or phosphatase (Ben‐Levy *et al*, 1995), we asked whether genomic variation could result in coordinated effects on different phosphosites on the same protein. Indeed, phRes traits of the same protein (i.e., different phosphosites on the same protein) had a higher chance to be targeted by the same phResQTL than random pairs of phosphosites (*P* < 3E‐8, one‐sided Fisher's exact test; Fig 4B).

Local phResQTLs might be caused by genetic variants directly affecting the phosphorylation state of a protein, for example, through the modification of residues close to a kinase or phosphatase binding site. In support of that notion, we found that proteins that were affected by a local phResQTL had a higher number of missense polymorphisms than proteins that only had distant phResQTLs (*P* < 1.3E‐7, one‐sided Wilcoxon rank‐sum test; Appendix Fig S9). In addition, phosphosites with a local phResQTL were closer to a missense variant than those with a distant phResQTL (Wilcoxon rank‐sum test: *P* < 7E‐4, Fig 4C). Further, if two phosphosites on the same protein were both affected by two different phResQTLs—one local and the other distant—the phosphosite with the local phResQTL was on average closer to a polymorphism than the distant one (*P* = 0.005, one‐sided paired Wilcoxon rank‐sum test; Appendix Fig S10). In summary, these results suggest that many local phResQTLs are caused by missense polymorphisms. These polymorphisms might cause differences in phosphorylation by changing the strength or accessibility of kinase and phosphatase binding motifs, by changing the localization of the protein, or by other covalent modifications of the protein.

### Phospho‐traits are strongly associated with physiological traits

**Figure EV2:**
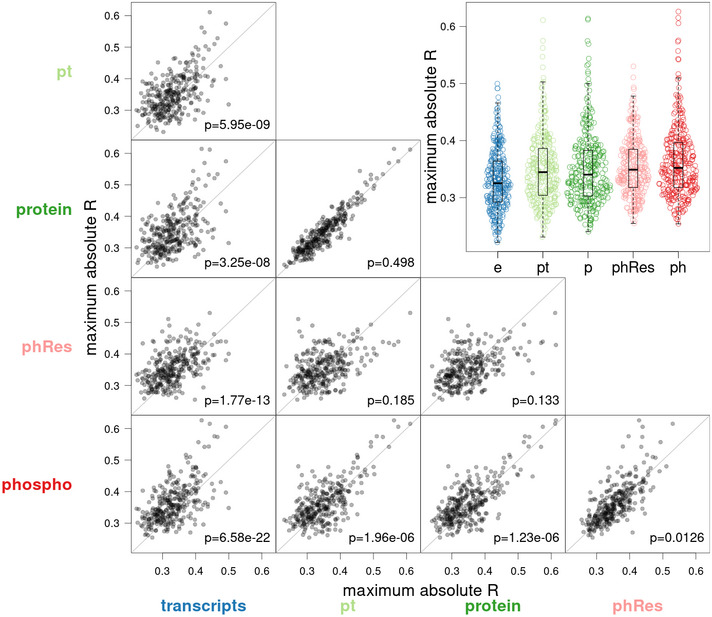
Molecular traits of each molecular layer were correlated with all compound resistance traits, respectively The analysis was restricted to genes that are available in all molecular layers. For each resistance trait, the most correlated feature of each layer was extracted (i.e., the most correlated transcript, pt trait, protein, phospho‐residual, and phosphopeptide). Each point represents the correlation between a compound resistance trait and the most correlated molecular feature. The gray lines represent the diagonal, and *P*‐values of paired Wilcoxon rank‐sum tests are indicated for each comparison. For example, there are more points above the diagonal than below when comparing phospho‐traits and transcripts, meaning that there are many growth traits where a phospho‐trait is better correlated than the best transcript. The inset in the top right shows the collective distributions of correlation coefficients at each layer.

In order to better understand the mechanisms by which genetic variation affects physiological traits, we integrated our molecular QTL data with two datasets that encompass chemical resistance traits (Perlstein *et al*, 2007) and morphological traits (Nogami *et al*, 2007) for subsets of the same yeast cross used in our study. In the former, growth was measured in the presence of 92 chemical compounds at multiple dosages each, resulting in a total of 307 trait measurements for 95 strains. The second dataset comprises 501 morphological parameters from high‐throughput imaging for 55 strains. We found that QTLs affecting resistance and morphology were more likely to overlap with p‐ and phQTLs than with eQTLs (Dataset [Link], [Link]). Further, protein and phosphopeptide abundance was, on average, more strongly correlated with the physiological traits compared with transcript abundance (Fig EV2 and Appendix Fig S11).

**Figure 5:**
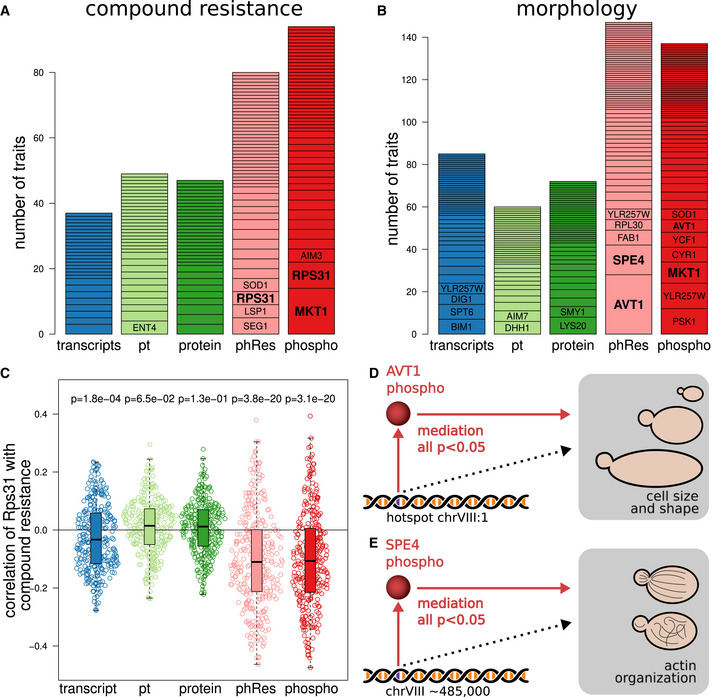
Integration with physiological phenotypes For a collection of 307 compound resistance traits (Perlstein *et al*, 2007), the highest correlators among molecular traits (i.e., transcripts, proteins, phospho‐traits, and their derived traits) were extracted. We then counted from which layer each top correlator originated (total bar height). The bar segments represent growth traits for which the same gene was the top correlator. Genes that are discussed in the main text are highlighted in bold. The analysis was limited to genes that were measured in all layers.Same as in a, but for 501 morphological traits (Nogami *et al*, 2007).Comparison of the distribution of correlation between Rps31 at different layers and compound resistance traits. Each observation (dot) is a single correlation between the RPS31 molecular trait and one compound resistance trait (*n* = 307 for each box). Difference from zero was determined using a one‐sample Wilcoxon rank‐sum test. The boxes extend from the first quartile to the third quartile of the data. The median is represented by the central line. Whiskers extend up to the most extreme data point within a distance of 1.5 times the interquartile range relative to the closest border of the box.Schematic representation of mediation analysis for the transmission of genetic variants toward morphological traits related to cell size and shape through Avt1 phospho‐residuals.Schematic representation of mediation analysis for the transmission of genetic variants toward morphological traits related to actin region size and brightness through Spe4 phospho‐residuals.

Due to those findings, we hypothesized that resistance and morphology traits were frequently conveyed through changes in abundance or phosphorylation of specific proteins. To test that, we identified the molecular trait that correlated most strongly with each cellular trait (resistance or morphology). Strikingly, phospho‐traits were more likely to be top correlators with physiological traits compared with transcript or protein abundance (Fig 5A and B).

The two phosphoproteins Mkt1 and Rps31 stood out in that they were most strongly correlated with multiple resistance traits. We observed a single, biphosphorylated phosphopeptide from Mkt1 at amino acid positions 354–378. Mkt1 was previously identified as harboring the causal variant of a hotspot on chromosome XIV (Smith & Kruglyak, 2008) and linked to multiple phenotypes including sporulation deficiency and decreased growth at high temperature (Sinha *et al*, 2006). As stated above, our data indicated that the *MKT1* hotspot regulates the abundance of mitochondrial ribosomal proteins, with enhanced effects on the protein levels compared with the mRNA levels. Thus, Mkt1 phosphorylation might be a key effector adapting the cellular biosynthesis to various stress responses.

In addition, phosphorylation of Rps31 stood out as being correlated with many resistance traits. The primary protein product of Rps31 is cleaved into ubiquitin and a small ribosomal subunit and thus influences both protein degradation and synthesis (Shrestha *et al*, 2019). The detected phosphosite on Rps31 (S122) is located in the ribosomal part, and its phosphorylation is negatively correlated with resistance (i.e., strains with less phosphorylated Rps31 are more resistant to the applied chemicals; Fig 5C). Hence, RPS31 may be involved in chemical stress resistance by regulating protein metabolism.

Avt1 and Spe4 phosphorylations were correlated with multiple morphological traits. Avt1 is involved in the import of large neutral amino acids into the vacuole (Russnak *et al*, 2001). The phospho‐residuals of Avt1 were correlated with traits related to general cell shape and size (Dataset [Link], [Link]), and all of those traits were targets of the same QTL within hotspot chrVIII:1 (*GPA1*/*STE20* hotspot, discussed below). Spe4 phospho‐residuals were correlated with traits related to actin (Dataset [Link], [Link]). Spe4 is required for the synthesis of spermine, and disruption of spermine synthesis was previously associated with abnormal distribution of actin (Balasundaram *et al*, 1991). Formal mediation analysis suggested that the phospho‐residuals of Avt1 and Spe4 significantly mediate the effects of genetic variation on the respective cell morphology traits (all FDR < 0.05 for both Avt1 and Spe4; Fig 5D and E).

### phQTL effects on regulatory network states

The above findings suggest that protein phosphorylation provides a crucial link between genomic variability and cellular (stress resistance) traits. Since these effects were likely mediated *via* signaling processes, we next investigated QTL effects on the states of signaling networks. To study QTL effects on the regulome, we integrated the five types of molecular traits with regulatory network information. We found that phResQTL targets were often functionally related to the putative causal genes underlying a given hotspot and/or targets of kinases that were affected by the same locus. The *IRA2* hotspot (XV:1) (Smith & Kruglyak, 2008) is an example for such a case. This hotspot affected resistance to six chemical compounds and had targets among all five types of molecular traits (Dataset [Link], [Link], Fig 3B). Ira2 inhibits the Ras/Pka pathway by promoting the GDP‐bound form of Ras2, which is crucial for the adaptation of cellular metabolism to conditions with different nutrient availabilities (Tatchell *et al*, 1985). Earlier work had shown that the polymorphisms of this cross in the *IRA2* coding sequence affect the activity of the Ras/Pka pathway (Smith & Kruglyak, 2008). Several targets of phQTLs in the *IRA2* hotspot were functionally connected to *IRA2* including a local phQTL targeting Ira2 itself, a phQTL targeting Cdc25, which promotes the GTP‐bound form of Ras2, and a phQTL targeting the Ras2 protein (Jian *et al*, 2010). In addition, we detected phQTLs for numerous downstream targets of Ras/Pka signaling at the *IRA2* hotspot (Dataset [Link], [Link]). Hence, our data reveal widespread differences in phosphorylation states of key signaling molecules in the Ras/Pka pathway upon genetic variation and therefore contribute to a better understanding of the molecular mechanisms through which this QTL hotspot affects cellular phenotypes.

Another example illustrating the effect of genetic variants on cellular signaling is the *HAP1* hotspot on chromosome 12 (chrXII:2) (Brem *et al*, 2002; Albert *et al*, 2014), which was previously shown to affect cellular growth in multiple conditions (Bloom *et al*, 2013). Hap1 regulates respiration in response to oxygen and iron deprivation (Verdière *et al*, 1985). Again, our analysis revealed that this locus has much more prominent effects on the phospho‐layer than on transcript or protein abundance (Fig 4A). To determine a potential regulator through which the HAP1 hotspot affects the phosphorylation state of these proteins, we leveraged a previously published dataset of strains with deleted kinases and phosphatases (Bodenmiller *et al*, 2010) (see Materials and Methods for details). Among all tested regulators, targets of the HAP1 hotspot were most significantly enriched in proteins changing their phosphorylation upon perturbation of the protein kinase PSK2, a known regulator of carbohydrate metabolism (Rutter *et al*, 2002). Strikingly, we found Psk2 itself to be a target of the HAP1 hotspot both on the transcript and on the phosphorylation layer. Together, these findings suggest a signaling cascade from the *HAP1* locus *via* altered Psk2 activity to Psk2 substrates.

**Figure 6:**
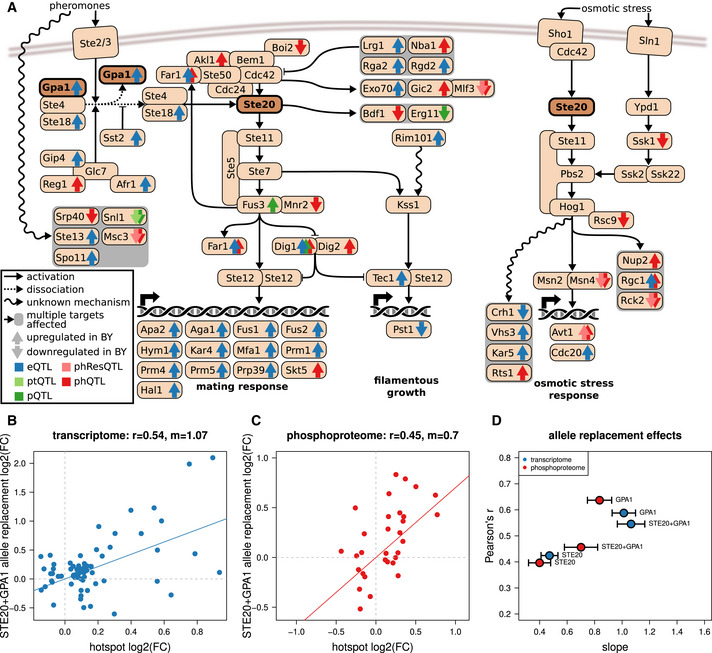
Multi‐layer effects of the hotspot around *GPA1* and *STE20* on chromosome 8 Gpa1 and Ste20 play key roles in the mating response pathway (left, through transcription factor complex Ste12/Ste12) and filamentous growth regulation pathway (middle, through transcription factor complex Tec1/Ste12). Ste20 additionally regulates the osmotic stress response pathway (right, through transcription factors Msn2/Msn4). Depicted are genes that are known to have a direct or indirect connection with *GPA1* and/or *STE20*. Genes that we found to be affected by the *GPA1*/*STE20* hotspot on any molecular layer are indicated with arrows in the respective color of each layer. The arrow orientation indicates the fold change direction. References for each known connection are given in Dataset [Link], [Link].Replacement of the reference alleles of STE20 and GPA1 causes changes in the transcriptome of the BY strain similar to those observed in the QTL cross. Each transcript that was targeted by a QTL at the GPA1/STE20 locus is represented as a blue dot. A linear fit of the effects of the double replacement and the effects of the hotspot is shown as a solid line. The slope (m) is indicated in the figure title.Effects of the allele replacements on the phosphoproteome are shown, with each affected phosphopeptide affected by a QTL at this locus being represented as a red dot. A linear fit of the effects of the double replacement and the effects of the hotspot is shown as a solid line. The slope (m) is indicated in the figure title.Pearson’s *r* and the slope of a linear model (m, as depicted in B and C) are shown for each replacement strain for the phosphoproteome and transcriptome, respectively. The standard error of each slope is shown as bars. Each dot shows the comparison of two genotypes based on three biological replicates per genotype.

The added value of integrating protein phosphorylation with transcript and protein abundance was even more apparent from a hotspot on chromosome 8 (chrVIII:1). *GPA1*, a key factor in the pheromone response pathway, was earlier shown to harbor a variant that explains effects on some targets of this hotspot (Yvert *et al*, 2003). The hotspot had a disproportionate effect on phospho‐traits, harboring only 69 eQTLs, 8 pQTLs, and 1 ptQTL, but 41 phQTLs (Dataset [Link], [Link], Fig 3B). Many of the hotspot eQTL targets (41%) could be found downstream of the mating pheromone response pathway, which supports *GPA1* as a causal gene. In addition, the hotspot region included the kinase *STE20*. Ste20 is the key activator of multiple mitogen‐activated protein kinase (MAPK) pathways, including not only the mating pheromone response but also the invasive growth regulation, regulation of sterol uptake, and osmotic stress response (Klipp & Liebermeister, 2006) (Fig 6A). *STE20* contains variants that co‐segregate with those in *GPA1,* making it a second candidate for a causal gene for this hotspot. Indeed, for up to 60% of the target genes of this hotspot we found a link to Ste20 and/or Gpa1 (Dataset [Link], [Link] and Dataset [Link], [Link]). Further, most of the phosphopeptides and phRes traits targeted by the hotspot corresponded to proteins phosphorylated by components of MAPK pathways downstream of Ste20 (51%; Fig 6A, Dataset [Link], [Link] and Dataset [Link], [Link]). GO enrichment analysis of targets of the hotspot at the transcript layer revealed enrichment for biological processes under the influence of *STE20* (Dataset [Link], [Link]). Together, these results suggest that the effects of the chrVIII:1 hotspot are due to the combined effects of the polymorphisms in *GPA1* and *STE20*. To test this hypothesis, we first generated allele replacement strains for *GPA1* and *STE20,* carrying the RM allele of one or both of the candidate genes in the BY background. We then characterized their transcriptome, proteome, and phosphoproteome. Indeed, replacing either *STE20* or *GPA1* with the RM allele each partially replicated the effects of the hotspot on its targets on the transcriptomic (*r* = 0.42 for *STE20* and *r* = 0.59 for *GPA1*; Fig 6B–D) and phosphoproteomic layers (*r* = 0.33 for *STE20* and *r* = 0.55 for *GPA1*; Fig 6B–D), including well‐characterized members of the pheromone response pathway such as Dig1, Dig2, and Far1. The double replacement strain showed changes comparable to those observed in the GPA1 replacement strain, indicating an (alleviating) epistatic relationship between STE20 and GPA1 for the traits considered here. Overall, our results support that *GPA1* and *STE20* contribute together to the downstream effects of this hotspot.

The examples of these hotspots illustrate how phQTL mapping provides information on signaling networks that is orthogonal to transcript and protein abundance data: Genetic variants often affect the phosphorylation states of gene products that are distinct from the genes affected by abundance changes (i.e., eQTL and pQTL targets). Furthermore, those protein activity changes can act either upstream (as in the case of the *GPA1/STE20* hotspot) or downstream (as in the case of the HAP1 hotspot) of genetic effects on transcript and/or protein abundance. Overall, we show that by quantifying the effects of sequence polymorphisms on multi‐layer molecular networks our integrated approach can provide clues toward reconstructing the molecular architecture underlying complex traits.

## Discussion

In this study, we present the first dataset that concurrently quantifies the effects of natural genomic variation on the transcript (eQTL), protein (pQTL), and protein phosphorylation (phQTL) layers in the same sample set. Whereas previous “omics” QTL studies have primarily focused on the genetic effects on the *abundance* of transcripts and proteins, our study integrates abundance with the phosphorylation *states* of proteins. This allows us to associate the consequences of genomic variation not just on transcript and protein abundance changes but also on phosphorylation‐mediated activation of signaling pathways and regulatory networks. The detailed dissection of the three hotspots around *IRA2*, *HAP1*, and *GPA1*/*STE20* exemplifies this notion.

Quantifying transcript and protein levels along with the activity state of the proteins indicated by phosphorylation from the same yeast culture maximized the comparability of the data, thus facilitating data integration. This three‐layer molecular profiling enabled the following findings: (i) RNA effects are generally transmitted to protein levels in a 1:1 relationship. There are, however, numerous exceptions, and specific classes of genes are subject to enhanced (e.g., mitochondrial ribosomes) and buffered (e.g., cytosolic ribosomes and nucleolus) protein‐level effects. (ii) A substantial fraction of phosphopeptides (44%) were under direct control of at least one QTL, independent of the abundance of its protein of origin. (iii) We observed multiple cases in which protein abundance effects were buffered (i.e., absent) at the level of phosphopeptides. (iv) QTLs affect multiple phosphosites on the same protein more often than expected by chance. (v) Protein phosphorylation is much more often affected by local missense variants than protein levels, indicating that structural destabilization of proteins through segregating variants is rare and presumably under strong negative selection. (vi) Phosphosites closer to sequence variants are more likely to be affected by them compared to other phosphosites on the same protein. (vii) The characterization of phosphorylation events in addition to transcript and protein abundance enabled the separation of upstream and downstream effects of genetic variants on signaling pathways as exemplified by the dissection of the IRA2, HAP1 and GPA1/STE20 hotspots. Importantly, as shown for HAP1, the same QTL can affect distinct sets of genes at different molecular layers. (viii) Although previous eQTL studies were able to identify causal genes underlying regulatory hotspots, the mechanism of their widespread effects often remained unclear. The additional information of altered protein phosphorylation states contributes significantly to understanding the mechanisms of QTL hotspots leading to the activation of pleiotropic downstream effects. (ix) Protein and phosphopeptide abundance and their respective QTLs (pQTLs and phQTLs) were more strongly connected with cellular traits, compared with transcripts and eQTLs. Furthermore, we identified specific phosphorylation traits that were the best predictors for a variety of physiological traits. This suggests that state changes of critical regulatory proteins often have more dramatic functional consequences for the cell than molecular abundance changes. In essence, protein abundance/activity can be seen as a summation of all upstream regulations on the transcript layer, which is why protein/phospho‐traits are more correlated with physiological traits than transcripts. For instance, the phosphorylation of Rps31 was, to our knowledge, never explicitly studied, but considering that the phosphorylation level of Rps31 is more tightly connected to many growth traits compared to its protein abundance (Fig 5C), we propose that the phosphorylation of Rps31 is critical for its function in stress responses. Further studies will be needed to fully reveal how phospho‐traits mediate QTL effects to physiological traits, for example, using amino acid replacements to modify the phosphosites of the mediator and evaluating whether the genetic effect on the physiological trait is then blocked.

Our findings also have implications for future work on complex human diseases and complex traits in other species. They suggest that mapping phospho‐traits and molecular states in general may greatly contribute to understanding the mechanisms through which GWAS loci act. We showed here that phosphorylation states harbor orthogonal information about physiological processes in addition to abundance of transcripts and proteins (Mehnert *et al*, 2020). Furthermore, our results support the notion that causal *cis*‐variants are often nearby the altered phosphosite. Thus, in order to interpret genomic variants affecting complex traits (including loci identified in human GWAS), it would help to leverage comprehensive catalogues of functional phosphorylation sites (Needham *et al*, 2019; Ochoa *et al*, 2020).

In conclusion, our study is a first step toward a better mechanistic understanding of genomic effects on multi‐layered cellular networks and physiological traits. This study captures some of the enormous complexity of how genetic variants influence signal transmission between different molecular layers. While the data here were obtained from just one standard growth condition, mapping phosphorylation states will be even more critical when aiming to understand the effects of genetic variation in the context of dynamic environmental cues. In addition, future work should address additional aspects of protein state changes, including other PTMs, changes in protein folding, protein complex formation, and protein–ligand interactions.

## Materials and Methods

### Sample preparation

All media were prepared in a single batch to limit experimental variability. The BYxRM yeast strain collection, which we obtained from Rachel Brem, was originally derived from a cross between the two parental strains, BY4716, an S288C derivative (MATα lys2Δ0), and RM11‐1a (MATa leu2Δ0 ura3Δ0 ho::KAN) (Brem *et al*, 2002). A subset of 129 strains were picked in random series of 16 (Dataset EV3), pre‐cultured in in‐house made synthetic dextrose medium (S.D., containing per liter: 1.7 g yeast nitrogen base without amino acids (Chemie Brunschwig), 5 g ammonium sulfate, 2% glucose (w/v), 0.03 g isoleucine, 0.15 g valine, 0.04 g adenine, 0.02 g arginine, 0.02 g histidine, 0.1 g leucine, 0.03 g lysine, 0.02 g methionine, 0.05 g phenylalanine, 0.2 g threonine, 0.04 g tryptophan, 0.03 g tyrosine, 0.02 g uracil, 0.1 g glutamic acid, and 0.1 g aspartic acid), and then grown in 115 ml fresh S.D. medium at 30°C until a maximal optical density at 600 nm (OD600) of 0.8 (± 0.1). In total, 180 cell cultures were successfully grown to OD600 of 0.8 and then subdivided as follows for the transcript and proteomic analyses, respectively. Of the 115 ml of cultures, 15 ml was collected, centrifuged at 2,000 *g* for 3 min at 4°C, transferred into an Eppendorf tube, and snap‐frozen in liquid nitrogen for transcriptomic analysis. The remaining 100 ml was processed for proteomic analysis essentially as described in Bodenmiller *et al* (2010). In short, 6.66 ml of 100% trichloroacetic acid (TCA) was added to the 100 ml culture media to a final concentration of 6.25% and the cells were harvested by centrifugation at 1,500 *g* for 5 min at 4°C and washed three times with cold acetone. The cell pellets were transferred into 2‐ml Eppendorf tubes and frozen in liquid nitrogen. Six different segregants were processed with three replicates each, and the parent strains had six (BY) and eight (RM) replicates each.

### RNA sequencing

#### RNA extraction

Total RNA was isolated from deep frozen aliquots of yeast pellets using the RiboPure™ RNA Purification Kit, yeast (Ambion), which includes a DNase treatment to eliminate contamination. RNA quality was assessed using RNA ScreenTape assay (Agilent). All RNAs were of very high quality (Dataset EV3 median RIN 9.8, minimal RIN 9.1).

#### RNA‐seq library preparation

cDNA libraries were prepared from poly(A) selected RNA applying the Illumina TruSeq protocol for mRNA using a total of 1 μg RNA per sample and 14 PCR cycles.

#### Sequencing

The cDNA libraries were sequenced on a HiSeq 2000 with 20 samples per lane (8 million reads per sample). The generated reads were stranded and single‐end, and had a length of 100 bp.

### RNA‐seq‐based genotyping

The BYxRM yeast cross has been widely used for QTL mapping. Microarray‐based genotype information at 2,957 markers is available for the segregants (Brem & Kruglyak, 2005). However, deep sequencing enables a more accurate genotyping of recombinant lines. To this end, we have exploited published resequencing data of the parental strains (Bloom *et al*, 2013) together with the RNA‐seq data generated here to infer the genotype of the segregants of the BYxRM cross using a method that we previously developed (Clément‐Ziza *et al*, 2014).

#### Resequencing data of the parental strains

Sequence variation information of the parental strains was obtained from http://genomics‐pubs.princeton.edu/YeastCross_BYxRM/; only calls with a MQ ≥ 30 were considered, which represents 42,769 polymorphic sites.

#### Genotype inferring from RNA‐seq data

RNA‐seq data were mapped to the *S. cerevisiae* reference genome (SaCer3) using TopHat 2 (Ref. Kim *et al*, 2013) with the following options: ‐‐min‐intron‐length 10 ‐‐min‐segment‐intron 10 ‐‐b2‐very‐sensitive ‐‐max‐multihits 1 ‐‐library‐type fr‐secondstrand. Read group information was added, and BAM files were sorted using Picard utilities (http://broadinstitute.github.io/picard/).

RNA‐seq data were further processed using the GATK pipeline (version 3.4‐46‐gbc02625) following the best practice guide (Van der Auwera *et al*, 2013). First reads containing exon–exon junction were split using the following options: ‐T SplitNCigarReads ‐rf ReassignOneMappingQuality ‐RMQF 255 ‐RMQT 60 ‐U ALLOW_N_CIGAR_READS; then, the variants were called using the UnifiedGenotyper at the 42,769 polymorphic sites identified in the resequencing data of the parental strains. Genotype calls where the GATK genotype score was below 40 (GQ ≤ 40) or that were covered with < 5 reads (DP ≤ 4) were considered as missing values.

#### Filtering and missing value imputing

For every polymorphic site between the parental strains, we compared the polymorphisms in the segregants and the parental strains to infer which allele was inherited. We further excluded polymorphisms (i) that could not be called (or correctly called) in the parental strains based on RNA‐seq data, (ii) that could be called in < 70% of the segregants, and (iii) with a lower allele frequency of < 20%. As previously discussed (Clément‐Ziza *et al*, 2014), genotypes called differing from the two direct flanking markers in more than one segregant (93 cases) probably denote erroneous genotype calls; the corresponding polymorphisms were excluded from the analysis. This resulted in 25,590 polymorphisms that were considered as genetic markers.

Finally, missing genotype values were inferred from the two neighboring polymorphisms if those were each within 20 kb of the polymorphism of interest and were inherited from the same parental strain (Clément‐Ziza *et al*, 2014) (Dataset EV2).

#### Assembling a set of markers for linkage analyses

Adjacent markers with the same segregation pattern across all segregants were collapsed into one unique marker, resulting in a set of 3,593 unique mapping genotypic markers (Dataset EV2). Thus, each marker represents a genomic interval in which all polymorphisms are in full linkage disequilibrium in the cross.

### Gene expression quantification and normalization

In previous work, we have shown that accounting for individual genome variations for RNA‐seq alignment improved gene expression quantification and deflated the number of falsely detected local eQTLs (Clément‐Ziza *et al*, 2014). Therefore, we used the strategy we had previously developed to map RNA‐seq reads. It consists of generating a strain‐specific genome, for each segregant, against which the corresponding reads are aligned.

First, we generated both strain‐specific genome sequences and strain‐specific annotations from the reference genome sequence (SaCer3), the reference genome annotation, and the genomic variations information (VCF files) previously created using RNA‐seq data. Then RNA‐seq reads were aligned to the corresponding strain‐specific genomes using STAR (ver 2.5.0a; Dobin *et al*, 2013) with the following options: ‐‐alignIntronMin 10 ‐‐quantMode GeneCounts. The gene‐specific read counts (strand‐specific) generated by STAR were used to quantify gene expression.

RNA‐seq coverage was computed by dividing the sum of the length of all reads by the sum of the length of the coding regions of the quantified transcripts per sample. Raw read counts were normalized using the *rlog* method of DESeq2 (Love *et al*, 2014). Normalized data were further corrected for effects due to culture batches using the non‐parametric empirical Bayes framework ComBat (Johnson *et al*, 2007) (Dataset EV1). Normalized and batch‐corrected read counts were corrected for gene length as follows for gene *i* in sample *j*:

$$c_{\mathrm{ij}}^{\prime }=\log_{2}\left(2^{\mathrm{cij}}\;\cdot \;\frac{1000}{l_{i}}\right),$$

where *l_i_* is the length of the coding region of gene *i*, excluding intronic regions.

### Proteomics

#### Sample preparation and phospho‐enrichment

Cell pellets were resuspended in lysis buffer containing 8 M urea, 0.1 M NH_4_HCO_3_, and 5 mM EDTA, and cells were disrupted by glass bead beating (five times for 5 min at 4°C, allowing the samples to cool down between cycles). The total protein amount from the pooled supernatants was determined by BCA Protein Assay Kit (Thermo, USA). Three milligrams of extracted yeast proteins was reduced with 5 mM TCEP at 37°C for 30 min and alkylated with 12 mM iodoacetamide at room temperature in the dark for 30 min. The samples were then diluted with 0.1 M NH_4_HCO_3_ to a final concentration of 1 M urea, and the proteins were digested with sequencing‐grade porcine trypsin (Promega, Switzerland) at a final enzyme:substrate ratio of 1:100 (w/w). Digestion was stopped by adding formic acid to a final concentration of 1%. Peptide mixtures were desalted using 3cc reverse‐phase cartridges (Sep‐Pak tC18, Waters, USA) and according to the following procedure: washing of column with one volume of 100% methanol, washing with one volume of 50% acetonitrile, washing with three volumes of 0.1% formic acid, loading acidified sample, reloading flow‐through, washing column with sample with three volumes of 0.1% formic acid, and eluting sample with two volumes of 50% acetonitrile in 0.1% formic acid. Peptides were dried using a vacuum centrifuge and resolubilized in 100 µl of 0.1% formic acid. Retention time standard peptides (iRT‐Kit, Biognosys, Switzerland) were spiked into the samples before they were analyzed by LC‐MS for total protein abundance (“non‐enriched samples”). The remaining 95 µl was supplemented with 300 µl of an overnight recrystallized and cleared‐up phthalic acid solution prepared by carefully dissolving 5 g of phthalic acid in 50 ml of 80% acetonitrile before adding 1.75 ml of trifluoroacetic acid. The samples were then enriched for phosphopeptides by incubating for 1 h under rotation with 1.25 mg of TiO_2_ resin (GL Sciences, Japan) preequilibrated twice with 500 µl of methanol, and twice with 500 µl of phthalic acid solution. Peptides bound to the TiO_2_ resin were then washed twice with 500 µl phthalic acid solution, then twice with 80% acetonitrile with 0.1% formic acid, and finally twice with 0.1% formic acid. The phosphopeptides were eluted from the beads twice with 150 µl of 0.3 M ammonium hydroxide at pH 10.5 and immediately acidified again with 50ul of 5% trifluoroacetic acid to reach about pH 2.0. The enriched phosphopeptides were desalted on microspin columns (The Nest Group, USA) with the protocol described above, dried using a vacuum centrifuge, and resolubilized in 10 µl of 0.1% formic acid. Again, retention time standard peptides (iRT‐Kit, Biognosys, Switzerland) were spiked into the samples before they were analyzed by LC‐MS for total peptide abundance (“phosphopeptides samples”).

#### LC‐MS data acquisition

The peptide concentration in all samples was measured on a NanoDrop at OD280 and normalized to allow injection ~1 µg of material into the mass spectrometer. The samples were randomized and then either injected individually for SWATH‐MS acquisition or pooled and injected in technical duplicates for shotgun acquisition. The LC‐MS acquisitions were performed on an AB Sciex 5600 TripleTOF coupled to a NanoLC‐2D Plus HPLC system (for the main QTL dataset), or on an AB Sciex 6600 TripleTOF coupled to a NanoLC‐1D Plus HPLC system (for the STE20/GPA1 validation). The liquid chromatographic separation and mass spectrometric acquisition parameters were essentially as described earlier (Selevsek *et al*, 2015). The peptide separation was performed on a 75‐µm‐diameter PicoTip/PicoFrit emitter packed with 20 cm of Magic C18 AQ 3 resin using a 2–35% buffer B at 300 nl/min (buffer A: 2% acetonitrile, 0.1% formic acid; buffer B: 98% acetonitrile, 0.1% formic acid). For shotgun experiments, the mass spectrometer was operated with a “top 20” method, with a 500‐ms survey scan followed by a maximum of 20 MS/MS events of 150 ms each. The MS/MS selection was set for precursors exceeding 200 counts per second and charge states greater than 2. The selected precursors were then added to a dynamic exclusion list for 20 s. Ions were isolated using a quadrupole resolution of 0.7 amu and fragmented in the collision cell using the collision energy equation.

$$CE=0.0625\;\cdot \;\frac{m}{z}‐3.5$$

with a collision energy spread of 15 eV. For SWATH‐MS acquisition, a 100‐ms survey scan was followed by a series of 32 consecutive MS/MS events of 100 ms each with 25 amu precursor isolation with 1 amu overlap. On both the AB Sciex 5,600 and 6,600 TripleTOF instruments, the sequential precursor isolation window setup was as follows: 400–425, 424–450, 449–475, …, 1,174–1,200 *m/z*. The collision energy for each window was determined based on the collision energy for a putative doubly charged ion centered in the respective window using the same equation as above with a collision energy spread of 15 eV.

All the MS data files were visually inspected and curated at this stage for low total ion chromatogram intensities, and the corresponding samples were reinjected when possible. For the main QTL dataset, this resulted in a final set of 179 SWATH data files for non‐enriched and 179 SWATH data files for phospho‐enriched samples (Dataset EV3) that were used for data extraction. Similarly, 40 DDA files for non‐enriched and 30 DDA files for phospho‐enriched samples were selected for database searching and library generation. The STE20 validation dataset additionally contained 16 phospho‐ and 16 non‐phospho‐data files.

#### LC‐MS database searching

The shotgun data were searched with Sorcerer‐Sequest (TurboSequest v4.0.3rev11 running on a Sage‐N Sorcerer v4.0.4) and Mascot (version 2.3.0) against the SGD database (release 03 Feb. 2011, containing 6,750 yeast protein entries, concatenated with 6,750 corresponding “tryptic peptide pseudo‐reverse” decoy protein sequences). For the search, we allowed for semi‐tryptic peptides and up to two missed cleavages per peptide. For the non‐enriched samples, we used carbamidomethylation as a fixed modification on cysteine residues and oxidation as variable modification on methionine residues. For the phospho‐enriched samples, we additionally allowed for phosphorylation as variable modification on serine, threonine, and tyrosine residues. The Sequest and Mascot search results were converted to pep.xml and then combined using iProphet (included in TPP version 4.5.2) both for the non‐enriched and for the phospho‐enriched samples. Both search results were filtered at 1% FDR by decoy counting at the peptide spectrum match (PSM) level, resulting in a total of 698,652 identified spectra, 26,893 unique peptides, and 4,310 proteins for the non‐enriched sample set; in the phospho‐enriched sample set, there were a total of 224,551 identified spectra, 16,515 unique peptides (thereof 14,466 unique phosphopeptides), and 2,333 proteins (thereof 1,911 phosphoproteins). Those data were compiled into two spectra libraries (one “non‐enriched” and one “phospho‐enriched”) using SpectraST (included in TPP 4.5.2) essentially as described earlier (Schubert *et al*, 2015), including the specific splitting of the consensus spectra when MS/MS scans identifying the same peptide sequence were recorded more than 2 min apart, also described earlier (Schubert *et al*, 2015). Those “split peptide assays” were given different protein entry names labeled Subgroup_0_ProteinX to Subgroup_N_ProteinX, respectively. The fragment ion coordinates for the peptides contained the top 6 most intense (singly or doubly charged) y or b fragment ions for each spectrum, excluding those in the SWATH precursor isolation window for the corresponding peptide. The non‐enriched assay library comprised assays for 19,473 peptides (thereof 18,074 proteotypic peptides matching a total of 3,119 unique proteins). The phospho‐enriched assay library comprised assays for 14,339 peptides (thereof 12,969 phosphopeptide sequences) or assays for 13,786 proteotypic peptides (thereof 12,678 proteotypic phosphopeptides, matching a total of 1,676 unique phosphoproteins). The same phospho‐ and non‐phospho‐libraries were used to extract both the QTL and the STE20 validation datasets.

The SWATH‐MS data extraction was performed separately for the main QTL and the STE20 validation datasets using the iPortal workflow manager (Kunszt *et al*, 2015) calling OpenSWATH (openMS v. 1.10) (Röst *et al*, 2014) and pyProphet (Teleman *et al*, 2015). The precursors were then realigned across runs using TRIC (Röst *et al*, 2016). The two resulting SWATH identification result files contained a total of 18,273 identified peptides (thereof 16,922 proteotypic peptides matching a total of 2,940 proteins) for the non‐enriched datasets; in the phospho‐enriched datasets, there were 13,748 identified peptides (thereof 12,412 phosphopeptides) or 13,218 proteotypic peptides (thereof 12,139 proteotypic phosphopeptides matching a total of 2,247 unique phosphoproteins). After alignment, we used a set of in‐house scripts to compare the chromatographic elution profiles of the various isobaric phosphopeptide isoforms matching a same delocalized peptide form (peptide sequence + number of phosphorylations) within each single run and to eventually group those co‐eluting phosphopeptide assays into the proper corresponding number of phospho‐peak clusters (labeled _cluster0 to _clusterN, respectively). The phospho‐peak clusters were then consistently re‐numbered across runs, and those were used as input to mapDIA (Teo *et al*, 2015) to select for the best suitable transitions and peptides for quantification. This resulted in the final peptide and protein quantification matrices for the non‐enriched and phospho‐enriched datasets that were used for further processing.

#### Preprocessing of proteome data

First, features detected after 7,000 s and those corresponding to decoys, reverse proteins, or not unique peptides were removed. We also removed fragments with oxidized methionine and their corresponding non‐oxidized fragments. Next, native retention times were converted to iRTs (Escher *et al*, 2012). Fragments corresponding to peptides, whose sequence was existing only in the reference proteome (i.e., BY1416 background) and not in RM11‐1a background were excluded from subsequent analysis. Normalization of the fragment‐level data and aggregation into peptides and protein‐level data were performed using mapDIA (Teo *et al*, 2015) with the following options: NORMALIZATION = RT 10, MIN_CORREL = 0.3, MIN_FRAG_PER_PEP = 2, MIN_PEP_PER_PROT = 1, and a maximum of 20% missing data for each fragment. Finally, abundance data were further corrected for effects due to culture batches and proteomics measurement batches using the non‐parametric empirical Bayes framework ComBat (Johnson *et al*, 2007).

#### Preprocessing of phosphoproteome data

First, fragments detected after 6,000 s, corresponding to non‐phosphorylated peptides (0P), corresponding to non‐unique proteins, and oxidized peptides were removed. As for non‐phosphorylated proteomics data, native retention times were replaced by iRT (Escher *et al*, 2012), and fragments corresponding to peptides whose sequence did not exist in the RM11‐1a background were excluded from subsequent analysis. To normalize the fragment‐level data and to aggregate them at the phosphopeptide cluster level, we used mapDIA (Teo *et al*, 2015). Fragments derived from phosphopeptides belonging to the same phosphopeptides cluster were aggregated together. The following mapDIA options were used: NORMALIZATION = RT 10, MIN_CORREL = 0.3, MIN_FRAG_PER_PEP = 2, and a maximum proportion of missing data of 20% for each fragment as for the non‐phosphoproteomics data. Data were corrected for culture batch and phosphoproteomics measurement batch effects using ComBat (Johnson *et al*, 2007).

### Derivation of the post‐transcriptional traits (protein abundance regressed to RNA levels) and phosphorylation levels (phosphopeptide abundance regressed to protein abundance)

In order to (i) separate the changes in protein abundance due to RNA changes from the post‐transcriptional specific regulation, and (ii) to distinguish changes in phosphopeptide abundance due to protein abundance changes from modification of the phosphorylation levels, we have generated regressed traits (as already proposed in Foss *et al*, 2011). The same procedure has been applied for both traits and will be detailed below using the phosphopeptide abundance example.

First, for each pair of corresponding traits (i.e., a phosphopeptide and its protein of origin) relative abundance across sample was normalized using a modified transformation to standard score (i.e., centering and scaling). To compute mean and standard deviation for this normalization, only the values corresponding to the samples in which measurements were available for both phosphopeptides and protein were used. Then, for each pair, normalized phosphopeptide data were regressed on protein data using a robust linear regression using a MM estimate (Yohai, 1987), initialized by an S‐estimate using Hubber’s weight function and using an M‐estimator as final estimate using Tukey's biweight function as implemented MM estimation option in the *rlm* function of the MASS R package (Venables & Ripley, 2002). The residuals of these regressions were averaged per strain and then used as traits in subsequent analyses.

### QTL mapping

We employed a previously developed QTL detection method based on Random Forest (Michaelson *et al*, 2010; Clément‐Ziza *et al*, 2014), with slight modifications. In short, Random Forest is used to model the phenotype using genetic variants as predictive variables. A combination of variable importance measures (described below) is used to score the effect of each variant on the phenotype. The significance of these scores is then determined by comparing them with an empirical distribution created through permutations. This approach is implemented in the “RFQTL” R package (http://cellnet‐sb.cecad.uni‐koeln.de/resources/qtl‐mapping/).

To correct for population substructure, we included population structure as covariates in the model, as we previously proposed (Michaelson *et al*, 2010), with the following modifications. First, the genotype matrix was normalized as described in Patterson *et al* (2006) (see equations (1) to (3) there). Then, we carried out a singular value decomposition on the normalized genotype matrix followed by an eigenvector decomposition. We then selected those eigenvectors corresponding to the top seven eigenvalues as covariates for the QTL mapping (additional predictors for growing the Random Forests). These first seven vectors explained more than 25% of the genotype variance (Dataset EV2).

We adapted the approach described in Clément‐Ziza *et al* (2014) using a different score describing the importance of each predictor in the Random Forest (variable importance measure, VIM). While previous work relied on the selection frequency as the VIM, i.e., the number of times a predictor was used in a forest, to quantify the importance of each predictor, we combined two previously published VIMs, RSS and PI (Liaw & Wiener, 2002), to compute an informative combined score serving as the VIM in our study. While the RSS describes the average reduction in the sum of the squared residuals after splitting a node with a predictor, the PI refers to the relative reduction in predictive accuracy after permuting the information for the respective predictor.

RSS and PI are combined in the following way to generate a more robust score *S_i_* for predictor *i*:

$$S_{i}=\max(0,RSS_{i})\;\cdot \;\max(0,PI_{i}).$$

We averaged replicates for the same strain to avoid the detection of false‐positive QTLs. Consecutive markers linked to the same trait and/or markers in high LD (Pearson’s *r* > 0.8) linked to the same trait were counted as a single linkage as we previously described (Clément‐Ziza *et al*, 2014).

### QTL hotspot detection

To formally identify hotspots in our dataset, the genome was divided into 40‐kb bins (293 bins, bins at chromosome extremities could be bigger). As proposed before (Brem *et al*, 2002), if the linkages were randomly distributed across the genome, the number of linkages in each bin would be expected to follow a Poisson distribution with the mean of $N_{\mathrm{linkage}}/N_{\mathrm{bin}}$. We use this distribution to estimate the highest number of linkages that a bin could contain at a probability lower than 0.01. These numbers were 30 for eQTLs, 13 for pQTLs, 9 for ptQTLs, 11 for phQTLs, and 4 for phResQTLs. Bins with a sufficient number of QTLs at a given molecular layer were considered as QTL hotspots. Consecutive bins on the same chromosome with enough QTLs were combined into single hotspots.

### Integration of physiological traits

We downloaded the compound resistance traits from Perlstein *et al* (2007). After merging replicates by taking their mean, the data included 307 distinct phenotypes, representing growth with 94 compounds at multiple exposure times and concentrations each. We restricted the data to the 95 strains for which where we could match the sample IDs between our and their data. The morphology traits were taken from Nogami *et al* (2007). Again, we took the subset of the 55 strains that were present in both their and our study. In total, there were 501 morphology traits, representing measurements of cells and organelles (including length, bud size, area, and nucleus size) and combined values thereof (including the sum of length and size of bud, and the product of nucleus area and nucleus density). In order to facilitate integration with our molecular QTLs, we used our sequencing‐based genotypes and RF‐based approach to remap QTLs for these resistance and morphology traits, as described above. In addition to merging markers in LD, we merged QTLs that affected the same compound but at different concentrations, according to the approach in Perlstein *et al* (2007). This resulted in 171 unique compound resistance QTLs and 115 morphology QTLs (gQTLs) at a FDR of 10%, compared with 124 in their original study.

We used Pearson’s correlation to investigate interrelationships between morphological and physiological traits. In order to summarize multiple phosphopeptides belonging to the same protein, we took the mean of the normalized, centered peptides of each protein. Although the different peptides in the same protein are not equivalent, this simplification was necessary in order to compare results for the different molecular layers.

Mediation analyses were performed with the "mediation" R package (Tingley *et al*, 2014). Bootstraps with 999 iterations were used to estimate *P*‐values of average causal mediation effects (ACME).

### Identification of local QTL

QTLs were considered to be local to their target if the QTL contained at least one genetic marker that had a correlation of 0.8 or higher with one of the markers that are directly up‐ or downstream of the target gene (Pearson’s *r*). For eQTLs, the target gene corresponds to the gene, whose expression is investigated; for pQTLs, it corresponds to the gene encoding the protein, whose abundance is studied; for phQTLs and phResQTLs, it corresponds to the gene encoding the protein of which the phosphopeptide is part of to provide context for the LD threshold, we also computed the genetic linkage in cM between neighboring markers a and b as follows:

$$cM=\frac{r}{n}\;\cdot \;100,$$

where *r* corresponds to the number of strains that experienced a recombination event between markers a and b, and *n* corresponds to the total number of strains (excluding parental strains).

To provide an estimate of genetic linkage that corresponds to the correlation threshold we used, we computed the average linkage distance of all marker pairs with a correlation of 0.8 or greater.

### GO enrichment

Gene Ontology (GO) enrichment analyses were performed using topGO (Alexa *et al*, 2006). We performed Fisher’s exact tests with the “weight01” algorithm and a minimal node size of 10. We always tested a group of significant genes against an appropriate background set of genes whose products could be measured on the respective molecular layer. For instance, we tested the pQTL targets of a hotspot for enrichment in comparison with other proteins whose levels also could be reliably determined. We employed this approach to avoid conflating measurement biases (e.g., for highly abundant proteins) with functional enrichment. We used annotations from SacCer3.

### Broad‐sense heritability estimation

Broad‐sense heritability estimates were computed based on replicate measurements of some strains, as described elsewhere (Bloom *et al*, 2013). We used six different segregants with three replicates each, as well as the parents with six (BY) and eight (RM) replicates each. In short, the “lmer” function from the lme4 R package was used to create a linear mixed‐effects model with the phenotype as the response and the segregant labels as random effects (Bates *et al*, 2015). The variance components $\sigma_{G}^{2}$ (the variance due to genetic effects, i.e., different segregants) and $\sigma_{E}^{2}$ (the error variance) were extracted, and broad‐sense heritability was calculated as $H^{2}=\sigma_{G}^{2}/\left(\sigma_{G}^{2}+\sigma_{E}^{2}\right)$. Standard errors were calculated using the delete‐one jackknife procedure, as proposed previously (Bloom *et al*, 2013). Random distributions of heritability estimates for each molecular layer were generated by permuting the strain labels and compared with the real distributions using a Mann–Whitney test. In order to be able to compare heritability estimates between the different molecular levels, we restricted the analysis to the set of 402 proteins where we had measurements for expression, protein, and (at least one) phosphopeptide.

### Polymorphisms in and around genes

To investigate the cause of local QTLs, we counted polymorphisms in the upstream region, downstream region, 3′ and 5′ UTRs, coding sequence, and amino acid sequence of each coding gene between the BY and RM genomes. We considered all SNPs reported by Bloom *et al* (2013). We excluded all genes with insertions and deletions from this analysis. UTR annotations were downloaded from www.yeastgenome.org in October 2017. If the UTR was reported multiple times with differing lengths, we used the largest annotation. The up‐ and downstream regions of a gene spanned 2 kb each and began at the outer borders of the UTRs relative to the gene of interest. The number of coding polymorphisms was defined as the number of amino acid changes in a protein between BY and RM. Multiple SNPs in the same codon were only counted once.

Figure 3C shows only genes with available UTR annotations and available measurements on the protein level. Genes with indels are not included.

For each phosphosite, we identified the closest polymorphism in the amino acid sequence space by computing the absolute difference of the position of the modified serine and all polymorphic amino acids within the protein and using the minimum of those distances. Note that phosphopeptides were only included in this study if they did not have any polymorphisms.

### Classification of QTLs based on their effects on the transcriptome and proteome

To analyze how QTLs affect the proteome, we classified eQTLs into three distinct classes: (i) eQTLs with “similar” effects on the transcriptome and proteome, (ii) “buffered” eQTLs, and (iii) “enhanced” eQTLs. We computed the effect *E* of a QTL at locus *i* on a molecular trait *j* as a log2 fold change by subtracting the average of the log2‐transformed trait values *t* of all strains with the RM allele at this locus from the average trait value for all strains with BY allele.

$$E_{\mathrm{ij}}={\bar{t}}_{j,G_{i}=BY}‐{\bar{t}}_{j,G_{i}=RM}$$

The eQTLs were classified based on the difference in their effects on the target transcript and the encoded protein:

$$\Delta ep_{i}=\frac{E_{\mathrm{ie}}}{\left|E_{\mathrm{ie}}\right|}\left(E_{\mathrm{ie}}‐E_{\mathrm{ip}}\right)$$

where *E_ie_* and *E_ip_* are the effects of eQTL *i* on the mRNA and protein levels, respectively. If the absolute difference |Δ*ep_i_* | was below 0.15, the eQTL effect was classified as “similar”. If Δ*ep_i_* was above +0.15, it was classified as “buffered”, and if the effect difference was below −0.15 (i.e., Δ*ep_i_* < −0.15), it was classified as “enhanced”.

ptQTLs at FDR < 10% that did not overlap with an eQTL for the same gene were added as a fourth class (“protein only”).

The effects of local and distant eQTLs on the proteome were compared, by generating simple least squares regression models for local and distant eQTLs separately. Here, we considered the log2 fold change on the protein level as the dependent variable and the log2 fold change on the transcript level as the independent variable. Models were generated with the *lm* function of the *stats* package in R (R Core Team, 2020).

### Validation of causal variation within STE20 and GPA1

#### Generation of allele replacement strains

The allele replacement strains generated in this study are derivatives of *Saccharomyces cerevisiae* BY4716 (Brachmann *et al*, 1998) (Table 1). Gene deletions were achieved by PCR‐based targeted homologous recombination replacing ORFs by indicated genes conveying amino acid prototrophy or drug resistance (Sheff & Thorn, 2004). In detail, the *STE20* ORF was replaced by either STE20 or polymorphic *RM11‐1a‐STE20* both linked to kanMX6 cassettes (pFA6a‐kanMX6) in the BY4716 MATalpha *lys2*Δ20 background. For the genomic modification of essential *GPA1*, an additional copy was ectopically expressed from plasmid (pRS316) containing the *LYS1* ORF for selection. Subsequently, the genomic *GPA1* ORF was replaced by either *GPA1* or polymorphic *RM11‐1a‐GPA1* both linked to natMX6 cassettes (pFA6a‐natMX6). Single clones were selected for according to drug resistance and loss of ectopically GPA1. All strains created were verified by PCR‐based methods and sequencing of targeted ORFs.

**Table 1:** Genetically modified *S. cerevisiae* strains used in this study.

*S. cerevisiae*	Genotype	Reference
**Sc1**	BY4716 *lys2*Δ*20*	Brachmann *et al* (1998)
**BY:GPA1‐BY:STE20**	**Sc1** Δ*ste20::WT‐STE20‐kanMX6* Δ*gpa1::GPA1‐natMX6*	this study
**BY:GPA1‐RM:STE20**	**Sc1** Δ*ste20::RM11‐1a‐STE20‐kanMX6* Δ*gpa1::GPA1‐natMX6*	this study
**RM:GPA1‐BY:STE20**	**Sc1** Δ*ste20::WT‐STE20‐kanMX6* Δ*gpa1:: RM11‐1a ‐GPA1‐natMX6*	this study
**RM:GPA1‐RM:STE20**	Sc1 Δ*ste20::RM11‐1a‐STE20‐kanMX6* Δ*gpa1:: RM11‐1a ‐GPA1 ‐natMX6*	this study

#### Sample preparation

Four replicates were grown for each of the four derived strains carrying either the BY4716 or RM11‐1a allele of *GPA1* and *STE20*. Samples were grown, harvested, and processed as described above for the full panel of strains.

#### RNA extraction and sequencing

RNA extraction was performed as described above. RNA extraction failed for sample BY:GPA1‐BY:STE20‐3. This sample was removed for all further analysis on all molecular layers. All other RINs were > 8.5. cDNA libraries were prepared with the Illumina TruSeq stranded mRNA kit, according to the manufacturer’s specifications, by the Cologne Center for Genomics (CCG) facility. The samples were sequenced on a single lane of an Illumina HiSeq 4000 to produce 2 × 100 nt reads.

#### Transcriptomic data processing

Reads were trimmed with Trimmomatic v0.36 (Bolger *et al*, 2014), with the following parameters differing from default settings: LEADING:0 TRAILING:0 SLIDINGWINDOW:4:15 MINLEN:25.

For quantification, the reads were mapped against strain‐specific versions of the BY‐genome that carry the expected polymorphisms within GPA1 and STE20. This was done to avoid additional mismatches for the strains carrying RM11‐1a alleles for STE20 and GPA1. Reads were mapped using bowtie2 (v2.3.4.1) (Langmead & Salzberg, 2012) with default parameters. Aligned reads were counted using *intersect* from the bedtools package (v2.27.1) (Quinlan & Hall, 2010), with the parameters ‐wb ‐f 0.55 ‐s ‐bed. Identical reads were only counted once.

The matrix containing the read counts of all samples where RNA extraction was possible (all except BY:GPA1‐BY:STE20‐3) was processed with the function *DESeqDataSetFromMatrix* to generate a DESeq dataset. The *DESeq* function was run on this object using the following design: ~batch + strain, where batch refers to the RNA‐extraction batch. For each pair of strains, we used the *results* function from DESeq2 to compute log2 fold changes and *P*‐values for differential expression.

### Enrichment of targets of kinases and phosphatases among *HAP1* targets

We tested the phosphoproteins targeted by the *HAP1* locus for enrichment in the previously annotated targets of a large number of kinases and phosphatases. Here, we used the data generated by Bodenmiller *et al* (2010) who measured changes in the abundance of phosphopeptides upon the deletion of selected kinases and phosphatases. We only considered target proteins that were reported to be phosphorylated or dephosphorylated at serine residues. We also considered proteins that were measured in Bodenmiller *et al* (2010) but not found to be regulated by any genetic perturbation. Among the 315 proteins that were measured in both studies, a total of 45 proteins had at least one phResQTL at the *HAP1* locus. We used one‐sided Fisher’s exact tests to assess the significance of the enrichment of targets of a kinase or phosphatase among the genes that were also targeted by the *HAP1* locus.

### Comparison with site‐specific fitness assays

A previous study by Viéitez and colleagues has investigated the fitness effects of a selection of phosphosites in a range of growth conditions (Viéitez *et al*, 2022). They did this by changing the phospho‐residue to an alanine each for 474 specific phosphosites, resulting in 474 strains, each carrying a different mutation. The fitness effects of these genetic perturbations were determined by observing deviations from an expected growth rate (S‐score) on a range of growth media. We integrated these findings with our results by classifying phospho‐traits based on their overlap with a phosphosite that was tested by Vieitez and colleagues. If a phosphopeptide contained at least one phosphosite that had a fitness effect in at least one condition (at FDR < 5%), we classified the phospho‐trait as being “functionally relevant” (39 phospho‐traits). All phosphopeptides that did not include a site with significant effects (but were tested) were classified as being “non‐functional” (37 phospho‐traits). Phosphopeptides that did not include phosphosites that were tested were not classified as being functional or non‐functional (2040 phospho‐traits). These were not further considered for this subanalysis.

## Author contributions

**Jan Grossbach:** Software; Investigation; Visualization; Methodology; Writing—original draft; Writing—review & editing. **Ludovic Gillet:** Data curation; Methodology; Writing—original draft; Data acquisition. **Mathieu Clément‐Ziza:** Conceptualization; Software; Formal analysis; Investigation; Methodology; Writing—original draft. **Corinna L Schmalohr:** Data curation; Software; Investigation; Visualization; Writing—original draft; Writing—review & editing. **Olga T Schubert:** Investigation; Writing—original draft; Writing—review & editing. **Maximilian Schütter:** Validation. **Julia S P Mawer:** Validation. **Christopher A Barnes:** Methodology; Writing—original draft. **Isabell Bludau:** Methodology; Writing—original draft. **Matthias Weith:** Investigation; Writing—original draft. **Peter Tessarz:** Supervision; Validation. **Martin Graef:** Supervision; Validation. **Ruedi Aebersold:** Conceptualization; Supervision; Funding acquisition; Methodology; Writing—original draft; Writing—review & editing. **Andreas Beyer:** Conceptualization; Formal analysis; Supervision; Funding acquisition; Investigation; Writing—original draft; Project administration; Writing—review & editing.

In addition to the CRediT author contributions listed above, the contributions in detail are:

AB, RA, and MCZ envisioned the study. LG, MCZ, and CAB performed the experiments. JG, CLS, MCZ, OTS, IB, MW, and LG performed the data analysis. MS and MG designed and engineered the allele replacement strains. JM and PT planned and performed the extraction and preparation of RNA for sequencing. AB, RA, PT, and MG acquired funding and supervised the work. All authors contributed to the writing of the manuscript.

## Disclosure and competing interests statement

In the course of the study, MCZ became an employee of Lesaffre International, which produces and sells yeasts and yeast‐based products. RA holds shares of Biognosys AG, which operates in the field of proteomics. The other authors declare no competing interests.

## Supporting information



Appendix

Expanded View Figures PDF

Dataset EV1

Dataset EV2

Dataset EV3

Dataset EV4

Dataset EV5

## Data Availability

The datasets and computer code produced in this study are available in the following databases:
RNA‐seq reads: ArrayExpress (E‐MTAB‐8146, https://www.ebi.ac.uk/arrayexpress/experiments/E‐MTAB‐8146/) MS data: Pride (PXD010893, http://proteomecentral.proteomexchange.org/cgi/GetDataset?ID=PXD010893) Analysis scripts: GitHub (https://github.com/Jan88/phosphoQTL)
