# Supplementary material for: The impact of genomic variation on protein phosphorylation states and regulatory networks
Source: Mol Syst Biol. 2022 May 16;18(5):e10712. doi: 10.15252/msb.202110712 (PMC9109056; doi:10.15252/msb.202110712)
Supplement: Supplementary file 1 — Appendix [file MSB-18-e10712-s002.docx]

***Appendix for***

**The impact of genomic variation on protein phosphorylation states and regulatory networks**

# Table of contents

**Appendix Text**

- Appendix Text S1

**Appendix Figures**

- Appendix Figure S1: Genotypes of parental strains and segregants.
- Appendix Figure S2: Correlation between molecular traits of the same gene.
- Appendix Figure S3: Broad-sense heritabilities calculated for the same genes at the five layers compared to each other.
- Appendix Figure S4: Average number of SNPs in and around genes affected by local QTLs at the respective molecular layer compared to permutations.
- Appendix Figure S5: Proportion of local and distant QTL that are located in hotspots.
- Appendix Figure S6: Transmission of local and distant eQTL effects to the proteome.
- Appendix Figure S7: Correlation between transcript- and protein-levels for functional groups of genes.
- Appendix Figure S8: Transmission of eQTL effects from the IRA2 hotspot.
- Appendix Figure S9: Number of missense mutations in proteins with phResQTL.
- Appendix Figure S10: Distances to the closest missense mutations for multiple phosphosites on the same protein.
- Appendix Figure S11: Correlation of molecular traits with morphological traits.

# Appendix Text

## Appendix Text S1

Chick *et al*. observed that, after correcting for transcript abundance, the significance of local pQTLs decreased more than that of distant pQTLs ^1^. Foss and colleagues detected markedly fewer local pQTLs than local eQTLs and concluded that protein levels are largely regulated in trans ^2^. However, the extent to which eQTLs impact protein levels might also depend on effect sizes of the eQTL. Indeed, earlier studies have established that the effects of local eQTLs are often larger than those of distant eQTLs ^3^. This was supported by our data: the average effect size of local eQTLs at FDR<10% was 26% larger than that of distant eQTLs.

Correspondingly, we found more local QTLs than expected by chance (p<2.2E-16 for all five types of molecular traits considered here, Fisher’s exact test), which is also recognizable by the presence of diagonal bands in the QTL maps (Supplementary Figures S3-S7). Transcripts with a local eQTL had on average lower expression levels than those that were only affected by eQTLs acting in trans, further emphasizing that local QTLs are easier to detect (p<2.2E-16, Wilcoxon’s rank sum test).

# Appendix Figures


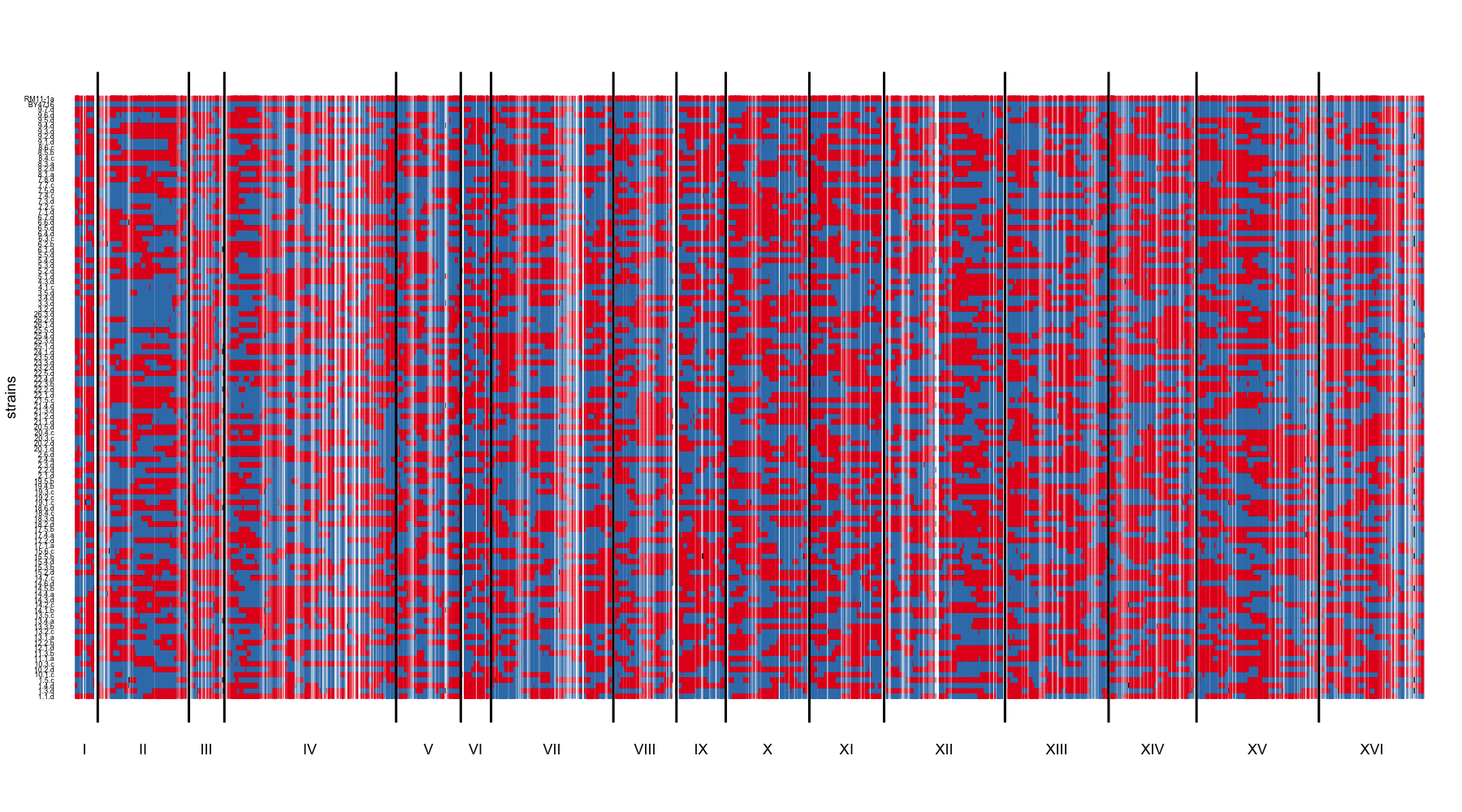


**Appendix Figure S1** Genotypes of parental strains and segregants. Alleles called from variant positions in transcripts are shown in red and blue depending of the genome of origin. Alleles from RM are shown in red while alleles from BY are shown in blue.

**
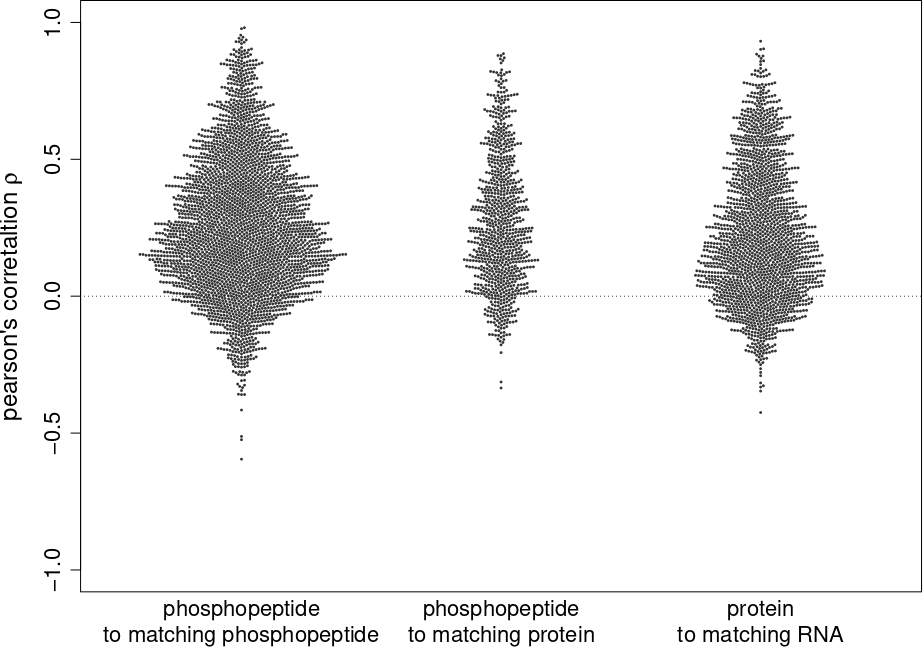
**

**Appendix Figure S2** Correlation between molecular traits of the same gene. The pearson’s correlations between phosphopeptides from the same protein (left), phosphopeptides and their host protein (middle) and that between protein levels and transcript levels of the same gene (right) are shown.


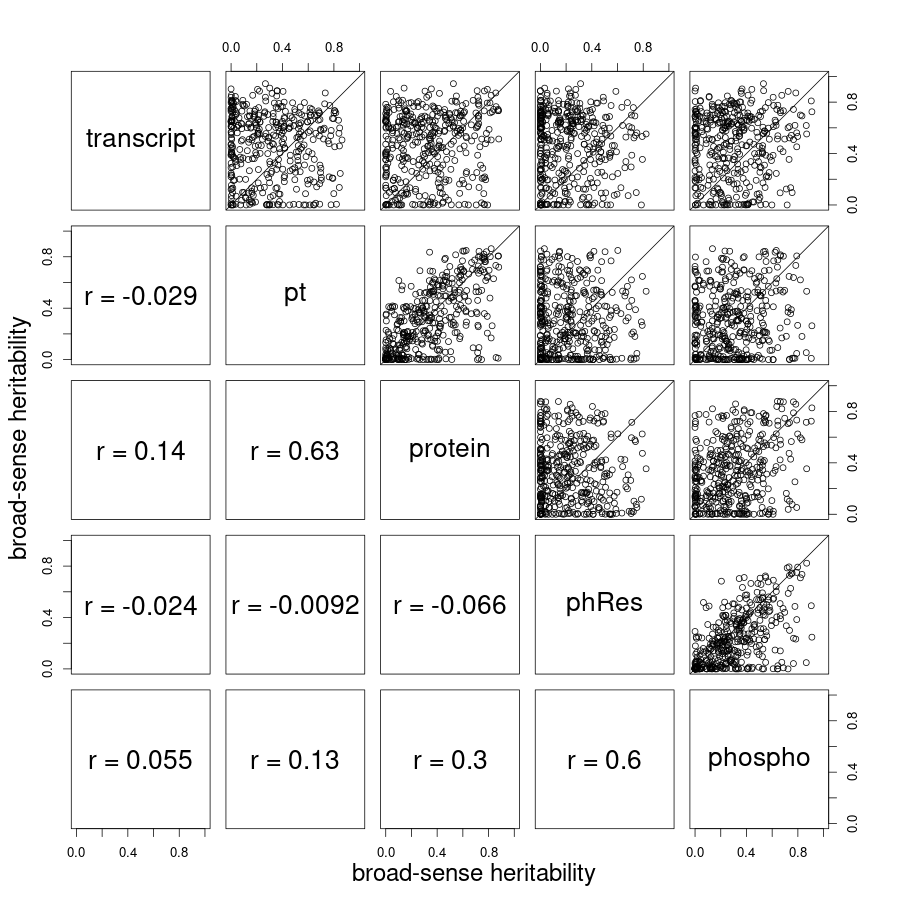
**Appendix Figure S3** Broad-sense heritabilities calculated for the same genes at the five layers compared to each other. Each dot in the upper right panels represents heritabilities for one gene, Panels in the lower left show pair-wise correlation coefficients. This analysis is based on the set of genes that were available in all layers. For the phospho traits, heritabilities were averaged over peptides belonging to the same protein.


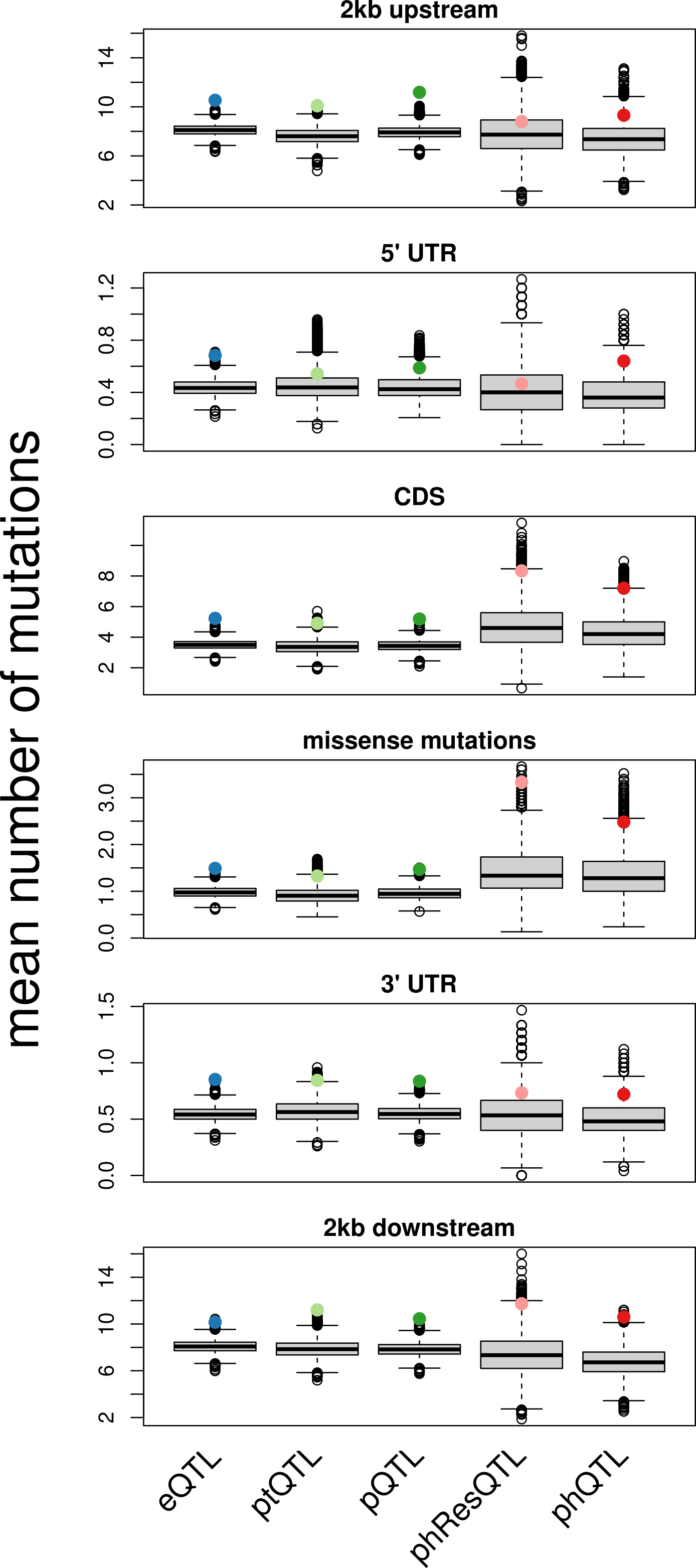


**Appendix Figure S4** Average number of SNPs in and around genes affected by local QTLs at the respective molecular layer compared to permutations. Each box represents 10.000 permutations, where we randomly drew n genes (with n being the number of genes with local QTL) from all genes with a QTL on this layer. The colored dots represent the oberseved average frequency of SNPs in each gene region for local QTL on the respective molecular layer. This Figure supports our conclusions from Figure 3A in the main text that genes affected by local QTL tend to be located in regions with more polymorphisms compared to genes affected by distant QTLs exclusively. The increased number of missense mutations in genes with local phResQTL is not to be expected by chance (permutation based p-value: p=0.0012).

**
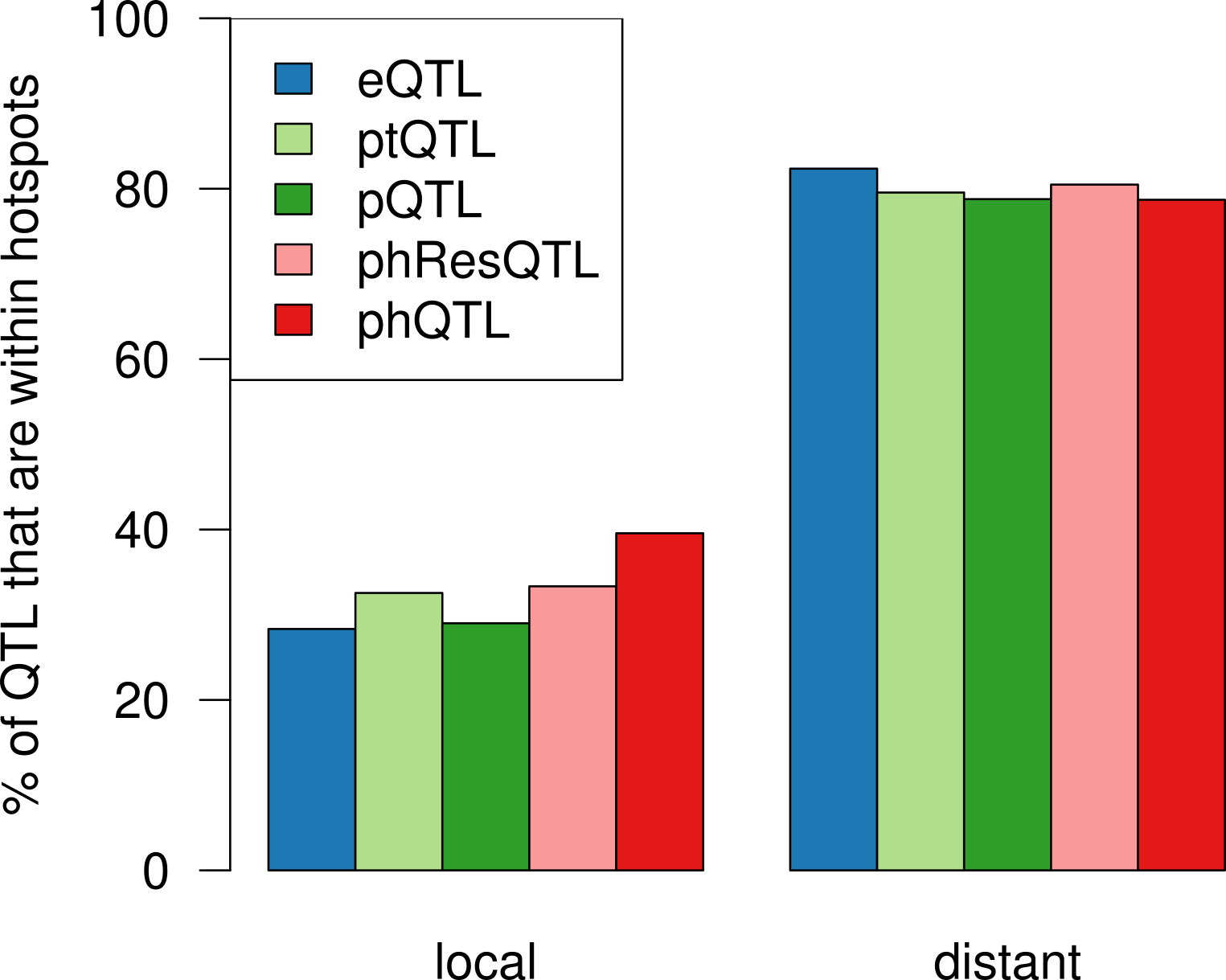
**

**Appendix Figure S5** Proportion of local and distant QTL that are located in hotspots, shown separately for each molecular layer.

**
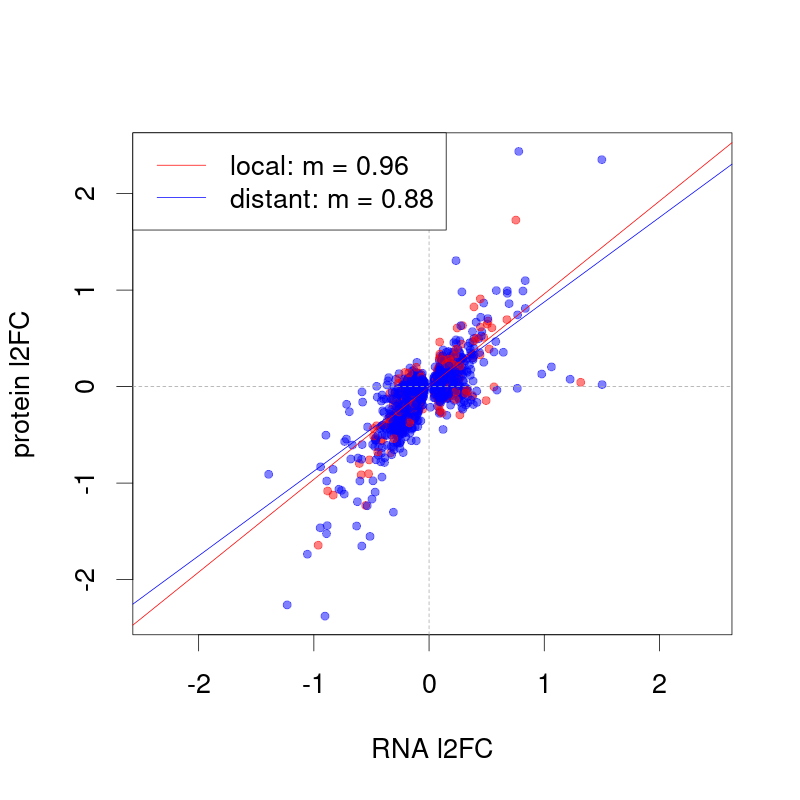
**

**Appendix Figure S6** Transmission of local and distant eQTL effects to the proteome. For all significant eQTL at FDR<10% the effect on the transcript- and protein-levels of the target gene is shown. Local eQTL are shown as red dots, distant QTL are shown in blue. Protein-effects were regressed against transcripts-effects for each class of eQTL. Both linear models had similar.

**
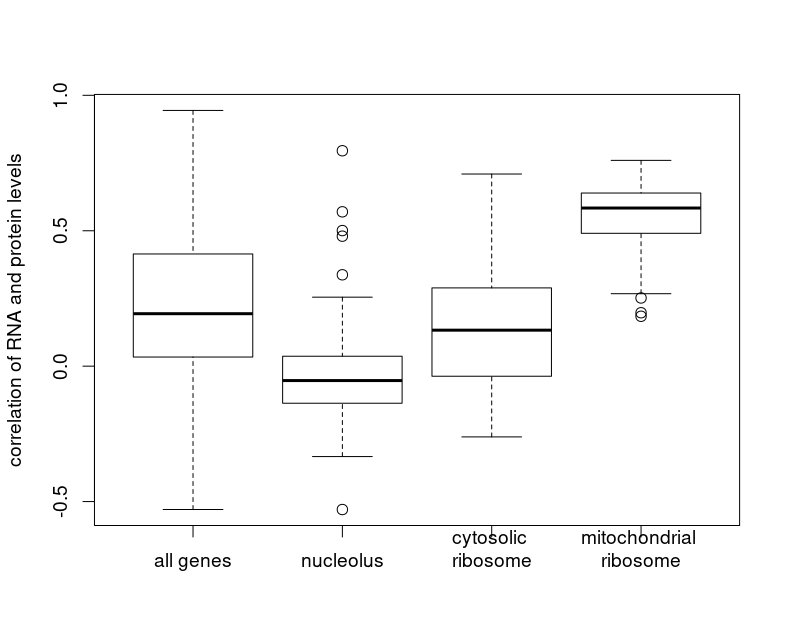
**

**Appendix Figure S7** Correlation between transcript- and protein-levels for functional groups of genes. Levels of transcripts and proteins were correlated for all genes with available transcript- and protein-levels, genes annotated to the nucleolus (GO:0005730), the cytosolic ribosome (GO:0022626) and the mitochondrial ribosome (GO:0005761). Outlier are shown as circles outside of the whiskers of the boxplots.

**
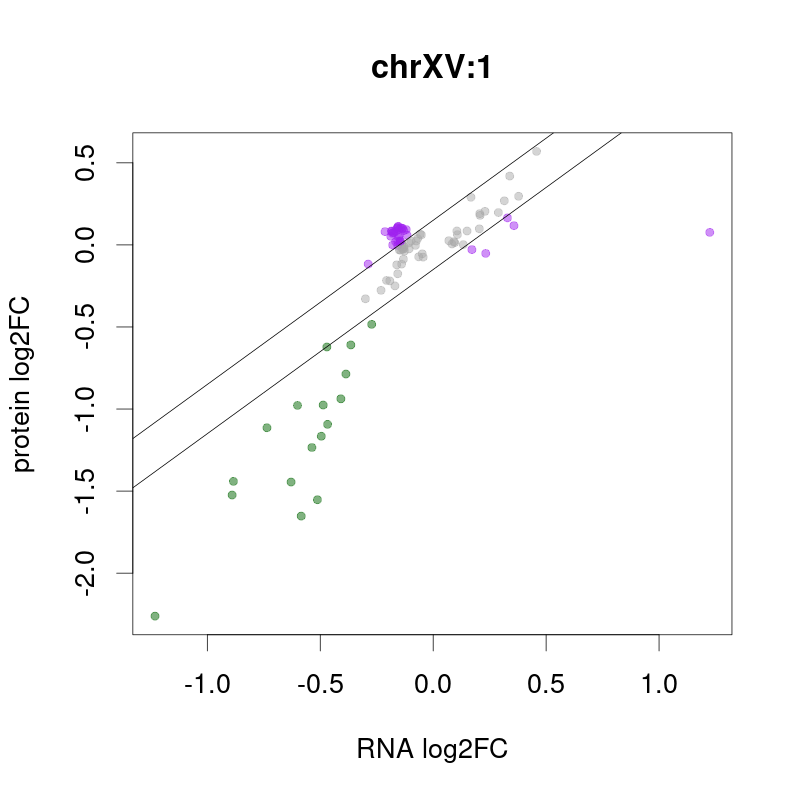
**

**Appendix Figure S8** Transmission of eQTL effects from the IRA2 hotspot. Effects of eQTL at the IRA2-locus on the transcript and protein levels of the target genes at FDR<10%. Each dot represents a target gene and is colored according to the difference in its effect on transcript and protein levels as described in the main text.

**
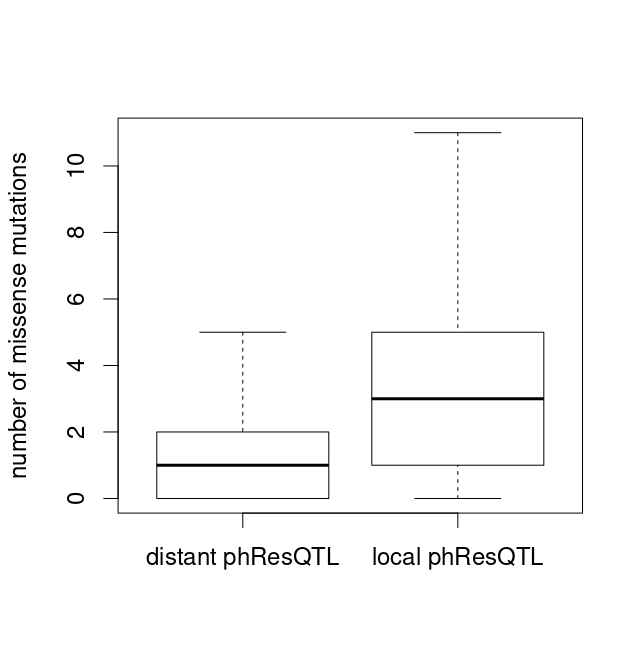
**

**Appendix Figure S9** Number of missense mutations in proteins with phResQTL. The amount of missense mutations is shown for proteins with only distant phResQTL and for proteins with a local phResQTL.

**
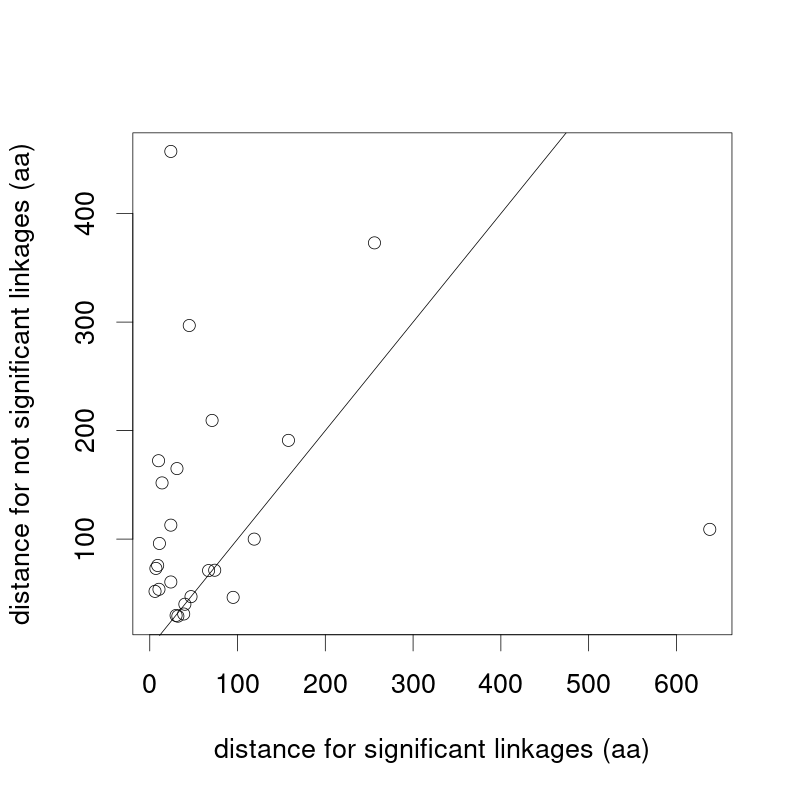
**

**Appendix Figure S10** Distances to the closest missense mutations for multiple phosphosites on the same protein. For proteins with one phosphosite with a local phResQTL and a different phosphosite with a distant phResQTL the distance of both phosphosites to the closest missense mutation in the sequence space is shown. A solid black line shows the diagonal. Dots above the line represent proteins for which the phosphosite with the distant phResQTL is located further away from the closest missense mutation than the phosphosite with the local phResQTL. Dots under the line represent proteins where the reverse is true.

**
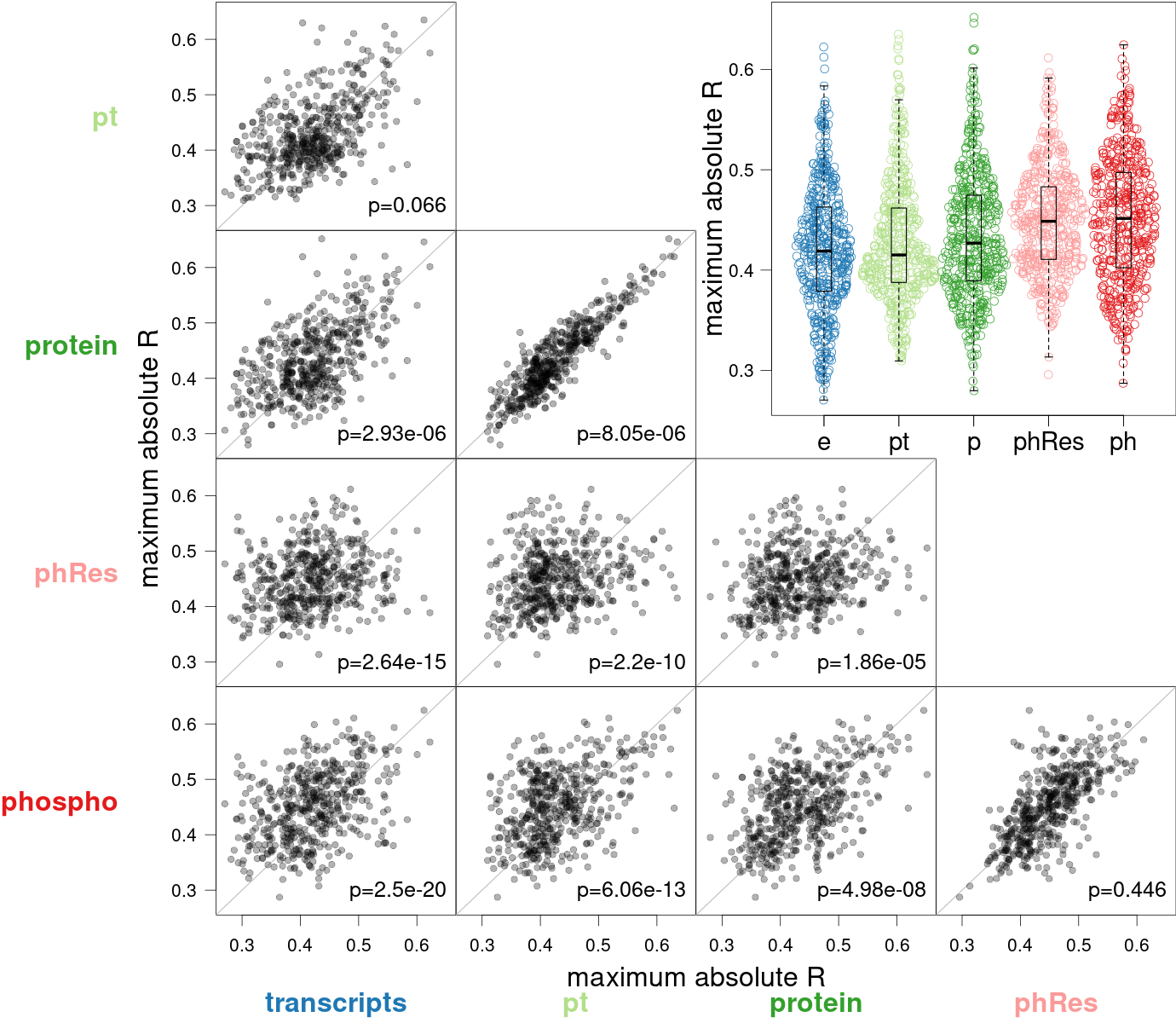
**

**Appendix Figure S11** Correlation of molecular traits with morphological traits. . The analysis was restricted to genes that are available in all molecular layers. For each morphology trait, the most correlated feature of each layer was extracted (i.e., the most correlated transcript, pt trait, protein, phosphoresidual and phophopeptide). Each point represents the correlation between a morphology trait and the most correlated molecular feature. The grey lines represent the diagonal, and p-values of paired Wilcoxon rank sum tests are indicated for each comparison. For example, there are more points above the diagonal than below when comparing phospho and transcripts, meaning that there are many morphology traits where a phospho-trait is better correlated than the best transcript. The inset in the top right shows the collective distributions of correlation coefficients at each layer.
