# Supplementary material for: The impact of genomic variation on protein phosphorylation states and regulatory networks
Source: Mol Syst Biol. 2022 May 16;18(5):e10712. doi: 10.15252/msb.202110712 (PMC9109056; doi:10.15252/msb.202110712)
Supplement: Supplementary file 2 — Expanded View Figures PDF [file MSB-18-e10712-s006.pdf]

Expanded View Figures

Figure EV1. Significant QTL at FDR < 10%.

A–E Associations between QTL and targets are shown as dots with X-coordinates showing the position of the QTL and Y-coordinates showing the position of the affected trait in the genome. Vertical bars indicate QTL hotspots while the diagonal consists of local QTL. Shown are eQTL (A), ptQTL (B), pQTL (C), phResQTL (D), and phQTL (E).

© 2022 The Authors

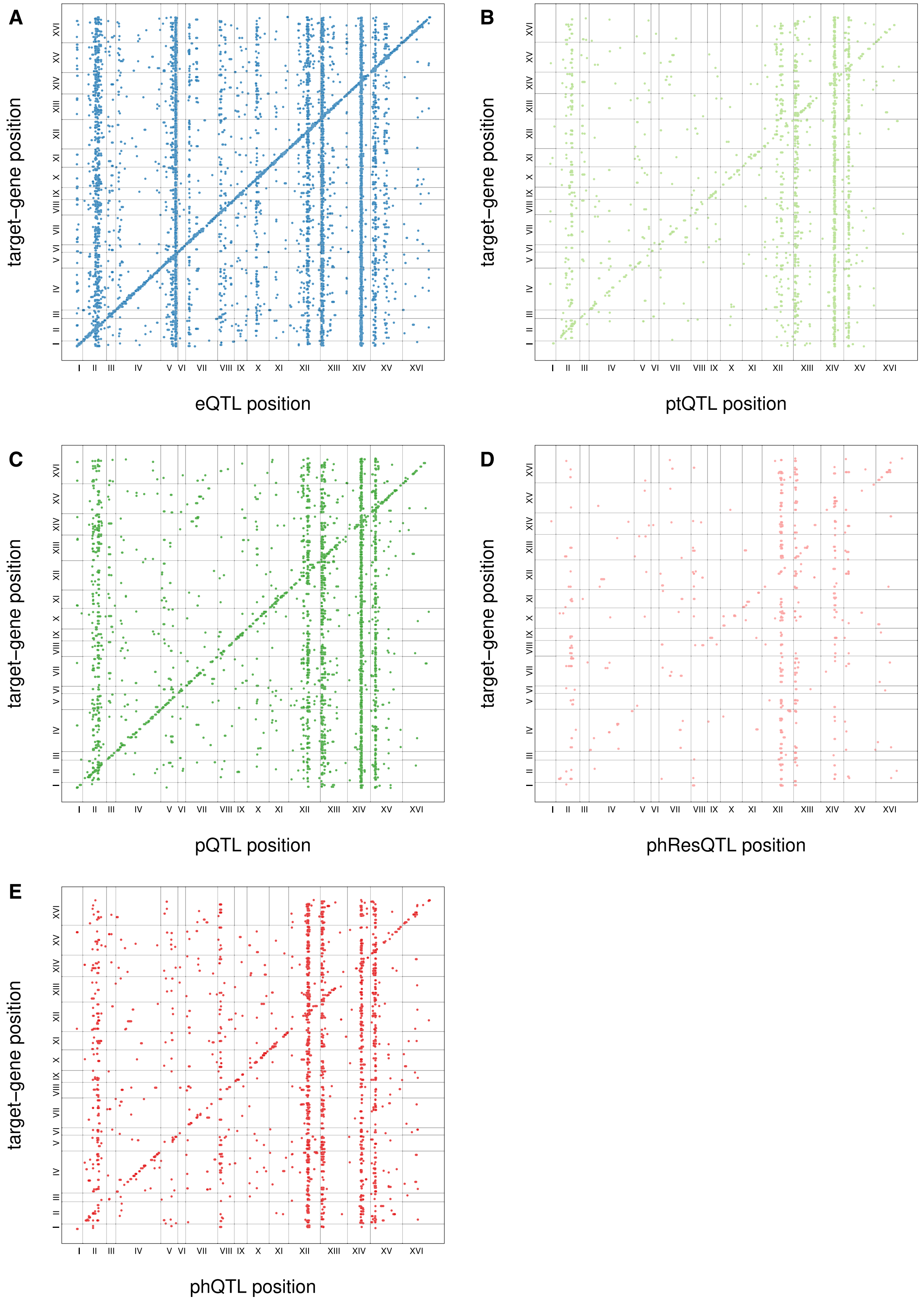

Figure EV1.

© 2022 The Authors

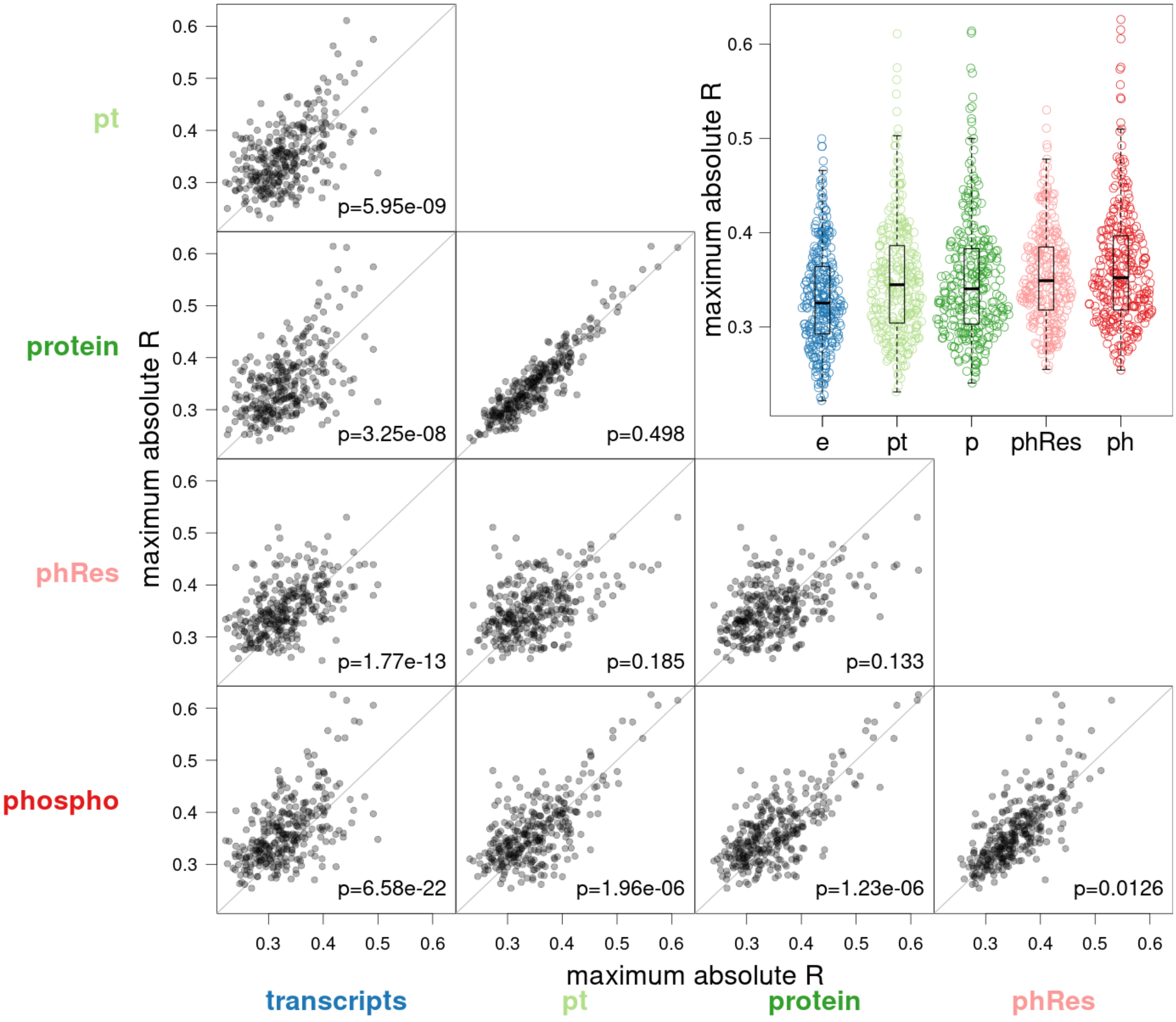

Figure EV2. Molecular traits of each molecular layer were correlated with all compound resistance traits, respectively.

The analysis was restricted to genes that are available in all molecular layers. For each resistance trait, the most correlated feature of each layer was extracted (i.e., the most correlated transcript, pt trait, protein, phospho-residual, and phosphopeptide). Each point represents the correlation between a compound resistance trait and the most correlated molecular feature. The gray lines represent the diagonal, and *P*-values of paired Wilcoxon rank-sum tests are indicated for each comparison. For example, there are more points above the diagonal than below when comparing phospho-traits and transcripts, meaning that there are many growth traits where a phospho-trait is better correlated than the best transcript. The inset in the top right shows the collective distributions of correlation coefficients at each layer.

 © 2022 The Authors
