# Supplementary material for: The impact of genomic variation on protein phosphorylation states and regulatory networks
Source: Mol Syst Biol. 2022 May 16;18(5):e10712. doi: 10.15252/msb.202110712 (PMC9109056; doi:10.15252/msb.202110712)
Supplement: Supplementary file 8 [file MSB-18-e10712-s005.pdf]

molecular systems biology

The impact of genomic variation on protein phosphorylation states and regulatory networks

Jan Grossbach, Ludovic Gillet, Mathieu Clément-Ziza3, Corinna Schmalohr, Olga T. Schubert, Maximilian Schütter, Julia Mawer, Christopher Barnes, Isabell Bludau, Matthias Weith, Peter Tessarz, Martin Graef, Ruedi Aebersold, and Andreas Beyer
DOI: 10.15252/msb.202110712

Corresponding author(s): Andreas Beyer (andreas.beyer@uni-koeln.de) , Ruedi Aebersold (aebersold@imsb.biol.ethz.ch)

Review Timeline:

Submission Date:	24th Sep 21
Editorial Decision:	28th Oct 21
Revision Received:	2nd Feb 22
Editorial Decision:	9th Mar 22
Revision Received:	8th Apr 22
Accepted:	12th Apr 22

Editor: Maria Polychronidou

Transaction Report:

(Note: With the exception of the correction of typographical or spelling errors that could be a source of ambiguity, letters and reports are not edited. Depending on transfer agreements, referee reports obtained elsewhere may or may not be included in this compilation. Referee reports are anonymous unless the Referee chooses to sign their reports.)

Thank you again for submitting your work to Molecular Systems Biology. We have now heard back from the three referees who agreed to evaluate your study. Overall, the reviewers acknowledge that analysing the effect of genetic variation on phosphorylation is a relevant contribution to the field. They raise however a series of concerns, which we would ask you to address in a major revision.

Most issues raised by reviewers #2 and #3 are relatively straightforward to address. However, reviewer #1 raises quite major concerns. Without repeating all of them, some of the more fundamental ones are the following:

- The conclusions drawn from the analyses shown in Figure 5 (i.e. that QTLs affecting resistance and morphology more likely overlap p and phQTLs than eQTLs) need to be better supported, ensuring that there is no bias in the analyses.

- The effect of hotspots on the reported correlations need to be assessed.

- Reviewer #1 also raises several methodological concerns regarding the QTL mapping, genotype analysis and trait integration.

- References to previous work on omics QTL mapping and trait variation in organisms other than human and yeast should be included, to offer a more comprehensive overview of the literature on the topic.

All issues raised by the reviewers would need to be satisfactorily addressed. Please contact me in case you would like to discuss in further detail any of the issues raised, I would be happy to schedule a video call.

On a more editorial level, we would ask you to address the following points:

REFEREE REPORTS

Reviewer #1:

This manuscript takes a classical Yeast biparental RIL population and reanalyzes it for a combination of transcripts, protein abundance and phosphorylation status to identify QTLs at these different levels and compare the genetics of these trait levels. This follows a similar omics integration efforts going from transcript to protein abundance to enzyme activity in plants using omics QTL analysis for example as conducted by Joost Keurentjes. The central difference in this effort is that instead of enzyme activity, the study measure protein phosphorylation. The connection of transcript/protein to phosphorylation state is a very interesting addition but I have a number of questions that are confusing me.

Figures and Results
In Figure 3A, what is the genomic variance in these values if random permutations were drawn. Although, these permutations would by default have to take out genes with no variation between the parents as by definition they cannot be found as QTLs.

In Figure 3C, I'm not fully following what this plot is showing. Is this the estimated effect size of the allelic swap at each QTL when the QTL influences both the protein and the RNA abundance? And is this across all the QTLs? It would seem that if this is this case that each hotspot should be treated as a separate analysis to test if there is differences in the relationship between hotspots in how the relationship plays out. Especially if some hotspots are ribosomal and possibly different as suggested.

In the statement that QTLs affecting resistance and morphology more likely overlap p and phQTLs than eQTLs and as shown in Figure 5, there are a couple of possible concerns. For example, by using individual transcripts with a pearson correlation when most of the cis-eQTL are in fact nearly bimodal could cause a potential bias against finding these associations. This would require a comparison of the trait distributions in the different classes to ensure that the use of pearson correlation for these tests isn't creating an ascertainment bias for or against a trait class.

Another note of caution is that this analysis did not appear to include an assessment of the role of hotspots in these correlations but instead treats the traits as a set of unconnected individuals. Often with eQTL hotspots the use of a summary value summing across the network of genes influenced by this locus finds a much higher relationship to any individual physiological phenotype as the network of transcripts controls for stochastic noise in individual transcripts. It would seem that this analysis should really work to account for the influence of the hotspot loci especially when single loci influence large suites of transcripts and proteins in conjunction. This significantly alters the assumption of omics trait independence that seems to be made in this analysis.

Admittedly, Figure 6, then goes into this network level analysis but it would seem that if these causal networks exist as shown in Figure 6 that the analysis in Figure 5 would need to directly assess if these networks create systematic associations that are missed when treating the omics traits as individual values.

Methods
I'm a bit unsure about the QTL mapping, this appears to be a GWA based analysis approach being applied to a biparental RIL. I understand the use of population structure in a GWAS setting in which there are demographic processes that can bias the genotype sampling and lead to false positives. But in a simple biparental structure, the 129 strains should represent a random sampling of the parental admixture potential with almost no population structure? Is there population structure in this random RIL collection? The concern is that by including a population structure matrix that may not capture any meaningful variation that the random forest model is over structuring the data leading to issues. Have the authors compared the model with and without structure in comparison to a gold standard set of loci to show that structure is not inflating error rates?

In the genotyping section, the manuscript comments that they increased their resolution by using resequencing data to infer segregant genotypes. But the paper should really discuss that the major limitation in resolution is only having 129 genotypes

which limits recombination to at best 1cM resolution for maybe a single large effect locus but more likely 5-10 cM for more polygenic traits no matter the genomic sequence.

In the integration of physiological traits, the study says that replicates were merged by taking their simple means. Does this mean that the data was collected by a perfect randomized complete block design? It is only proper to take a simple mean when a RCBD was utilized and there is not blocking structure in the data that could bias the mean. Similarly, are the data normally distributed to justify the use of pearson correlation?

I am a bit puzzled by the definition of a local QTL. The sentence says that if the QTL contains at least one genetic marker with a 0.8 Pearson correlation to a marker in the gene it is considered local. What does this mean in actual units of Linkage. I did a rough back of the envelope calculation and this almost seems to be creating at least a nearly 20cM window around the causal locus to call local. This seems extremely large. This definition of local should be placed in the linkage terminology to allow the reader to interpret the window being used. Also, marker data isn't normally distributed and a pearson correlation would be inappropriate.

Editorial Suggestions
As an editorial comment if the manuscript is meant to appeal to a general audience, it seems somewhat odd to have only yeast and human citations on the application of omics QTL mapping to understand trait variation. This seems to preclude the large body of literature on these topics from Drosophila, C. elegans, Arabidopsis, Maize and Rice, to mention a few. Especially as there are plant papers going from eQTL to enzyme activity in a similar manner as here.

In a similar vein, the statement on page 9 that hotspots are typically transcription factors or kinases is not supported in the plant literature. In the plant literature, these are as often enzymes or other non TF/Kinase genes. This is an example of where a broader reading of the omics literature beyond yeast and humans could contribute to a manuscript with broader appeal.

Reviewer #2:

In this manuscript, Grossbach et al. address an important question: what is the extent and properties of natural genetic variation of the phosphoproteome? They describe an experimental study of systems genetics where three layers of molecular traits are mapped in yeast: transcripts abundance (eQTL), proteins abundance (pQTL) and phosphopeptides abundance (phQTL). In addition, they applied a regression analysis to search for QTLs that account for post-transcriptional control of protein abundance (ptQTL) and for phosphorylation levels relative to protein abundance (phResQTL). To characterize these genetic regulators, the authors compared their locations, their redundancy, their nature and their correlation to physiological traits. They also validated the effects of two linked variants using allele-replacement strains. As expected, the results obtained at the RNA and protein levels are not novel and are totally concordant with previous studies. Results on phosphopeptides are totally novel and have important implications: genetic variation of phosphorylation states per se does exist and it is highly correlated to physiological traits. This should invite colleagues conducting human GWAS to include phosphoproteomics in their designs.

The work is of excellent quality. First, the authors spent considerable efforts to obtain a consistent dataset. The yeast cross they used has previously been extensively studied for mRNA and protein levels. It would have been much easier to use published datasets, and "simply" quantify phosphoproteomes of previously-characterized segregants. Instead, they re-generated all the data themselves, so that all three molecular layers were quantified from the exact same samples, which is important to avoid biases of lab-to-lab and batch-to-batch variability. Second, the analysis of the dataset is thorough, with careful statistical inferences and interpretations of the various molecular effects.

Overall the work is of very high quality and potential impact. I have minor comments that may help the authors improve the manuscript.

1. What is the correlation between heritabilities of the different layers? (data of fig2b). It would be interesting to visualize if there are cases (genes) where an elevated heritability is specifically seen for the phosphoproteome layer. Note that this is independent from the QTL results: heritability does not depend on mapping power.

2. Although not needed for this first phosphoQTL study, the discussion could mention possible future experiments that can completely demonstrate the mediation effects of phospho sites (eg. those of fig 5d-e). In principle, it may be possible to combine allele-replacement at the phResQTL with specific amino-acid substitutions on the target protein to evaluate if the genetic effect on the physiological trait
is blocked.

3. Are the present results on eQTL, pQTL and ptQTL consistent with those of Brion et al. (https://elifesciences.org/articles/60645) who deconvoluted the protein/RNA layers in the same yeast cross?

4. Fig6d: Although allele-replacement of STE20 has a moderate effect alone, it has no effect in the context of GPA1-STE20

double replacement. Can the authors discuss this deeper?

- page 18: the IRA2 hotspot is not flagged on Fig3b and we don't know where to look on the Fig panel.
- page 20, typo in legend: "by the a QTL".
- page 21 bottom. Replace "Further" by "(iii)" ?

Reviewer #3:

Grossbach et al present a genetic analysis of transcript, protein and protein phosphorylation in a widely-used yeast panel. They report QTL across all omic-domain and extensively examine and dissect the levels of regulation that give rise to the observed patterns of genetic effects. The impact of genetics on phosphorylation is widespread and is shown to play a significant role in chemical sensitivity and morphological traits that were previously described for the yeast panel. The genetic analysis of phQTL appears to be novel and will undoubtedly spur attention to this important, emerging area of mass-spec proteomics.

The presentation is clear, concise and methods are appropriate and well documented. I have no major concerns.

Minor points:

The authors avoided a common statistical error by not taking ratios of ph-peptide to total-peptide abundance. The use of residuals (ph-peptide regressed on total) is an important (positive) feature of the analysis. Although it is unlikely to make a big difference, I wonder why they did not instead directly use the total peptide abundance as a covariate (forced inclusion in the RF models). This would more correct approach - residual regression is a corner cutting approximation with some potential pitfalls (https://doi.org/10.1002/gepi.20607).

The RF QTL mapping method appeared in 2010 but has not been widely adopted. Could this choice be further justified? Do the authors expect non-additive QTL effects? Could the RF QTL be compared to a standard method (e.g. HK regression) or compared to previously reported eQTL and pQTL to confirm that similar results are being obtained? If new QTL are emerging from the RF analysis, a comment and closer look at why would be helpful.

Please add a comment in the main text to indicate that polymorphic peptides were removed from analysis. I found this deep in the Methods but fretted about it until I got there.

Reviewer #1:

This manuscript takes a classical Yeast biparental RIL population and reanalyzes it for a combination of transcripts, protein abundance and phosphorylation status to identify QTLs at these different levels and compare the genetics of these trait levels. This follows a similar omics integration efforts going from transcript to protein abundance to enzyme activity in plants using omics QTL analysis for example as conducted by Joost Keurentjes. The central difference in this effort is that instead of enzyme activity, the study measure protein phosphorylation. The connection of transcript/protein to phosphorylation state is a very interesting addition but I have a number of questions that are confusing me.

We very much thank the reviewer for appreciating the novelty of our work.

Figures and Results

In Figure 3A, what is the genomic variance in these values if random permutations were drawn. Although, these permutations would by default have to take out genes with no variation between the parents as by definition they cannot be found as QTLs.

The analysis in Figure 3a compares the frequency in genes affected by local QTLs (bars) to the background frequency of variants across all genes considered in the analysis (horizontal dashed line). This analysis clearly shows an enrichment of variants in genes affected by local QTLs (all bars end above the dashed line), which is consistent with the common interpretation that many or even most local QTLs are in fact cis-QTLs.

The Reviewer raises a valid point in that we do not show whether the deviation of genes with local QTL from the genomic background in Fig 3a is statistically significant, or is likely to occur by chance. First, we wish to note that the fact that all bars end above the background (dashed line) already indicates a non-random association between increased numbers of variants and local QTLs. (If there was globally across all layers no association, one would expect fluctuations above and below the background.)

In order to further strengthen our conclusions we conducted a permutation analysis. We generated background distributions for each layer individually by randomly drawing n genes (with n being the number of genes with local QTL) from all genes with a QTL on this layer. The distribution of the frequencies of the respective kind of genetic variation from 10.000 permutations are shown as boxplots below, and the colored dots represent the observed average frequency of SNPs in each gene region for local QTL on the respective molecular layer.

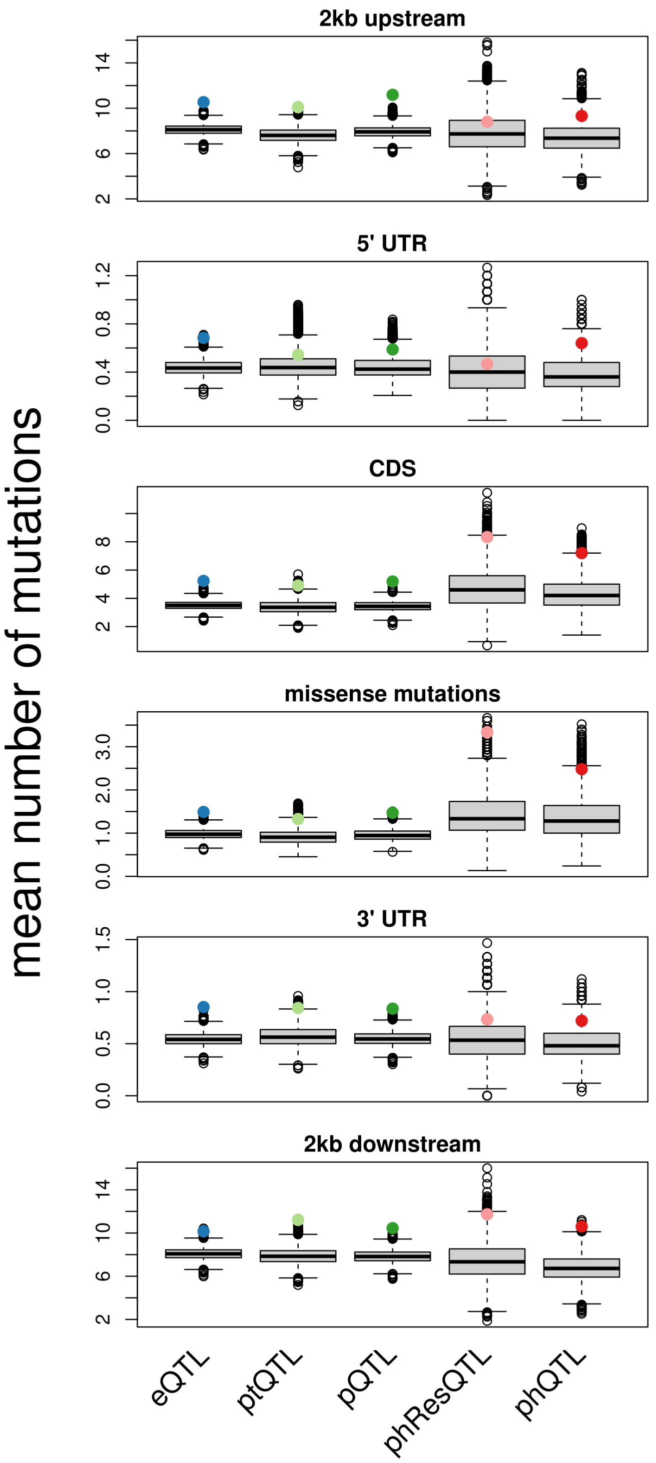

In general, the permutations support our conclusions that genes affected by local QTLs tend to be located in regions with more polymorphisms compared to genes affected by distant QTLs exclusively. Importantly, the increased number of missense mutations in genes with local phResQTL is not to be expected by chance (permutation based p-value: p=0.0012).

We have added this new figure as Appendix Figure S4 to the submission.

In Figure 3C, I'm not fully following what this plot is showing. Is this the estimated effect size of the allelic swap at each QTL when the QTL influences both the protein and the RNA abundance? And is this across all the QTLs? It would seem that if this is this case that each hotspot should be treated as a separate analysis to test if there is differences in the relationship between hotspots in how the

relationship plays out. Especially if some hotspots are ribosomal and possibly different as suggested.

Figure 3c shows the effects of QTLs on transcript and protein levels. Each dot represents the log2-transformed fold changes of BY versus RM alleles corresponding to an association of a QTL with a gene. Reviewer 1 is right, they could be interpreted as estimates of allelic swap effects. This analysis includes all significant eQTLs and ptQTLs. We have edited the figure description to make this more explicit.

We also analyzed individual hotspots in a subsequent analysis (see the two last paragraphs of the corresponding section and Supplementary Figure S7 in the old version/Appendix Figure S8 in the current submission) where we focused on the IRA2 and the MKT1 hotspots:

"This notion was further supported by detailed investigation of individual hotspots with heterogeneous eQTL and pQTL effects. For example, the effects of the *IRA2* hotspot (XV:1) on transcript levels of cytoplasmic ribosomal genes was not transmitted to protein levels, but the same locus affected protein levels of genes related to mitochondrial respiration without changing their transcript levels (Appendix Fig S8). While the effects of the *MKT1* hotspot (XIV:1) on the protein levels of cytosolic ribosomes were buffered, the effects of the same locus on the protein levels of mitochondrial ribosomes were enhanced."

Here we did not consider protein specific QTL, but focused on the transmission of eQTL effects to the proteome. Below we show a plot equivalent to Figure 3c, only subsetting to individual hotspots (the plot for chrXV:1 corresponds to Supplementary Figure S7 in the old version/Appendix Figure S8 in the current submission). Even when considering hotspots individually, this does not change the conclusions drawn in the manuscript. We observe both buffered and enhanced eQTL effects for individual hotspots, demonstrating that the different eQTL effect classes are not a function of the hotspot but the target.

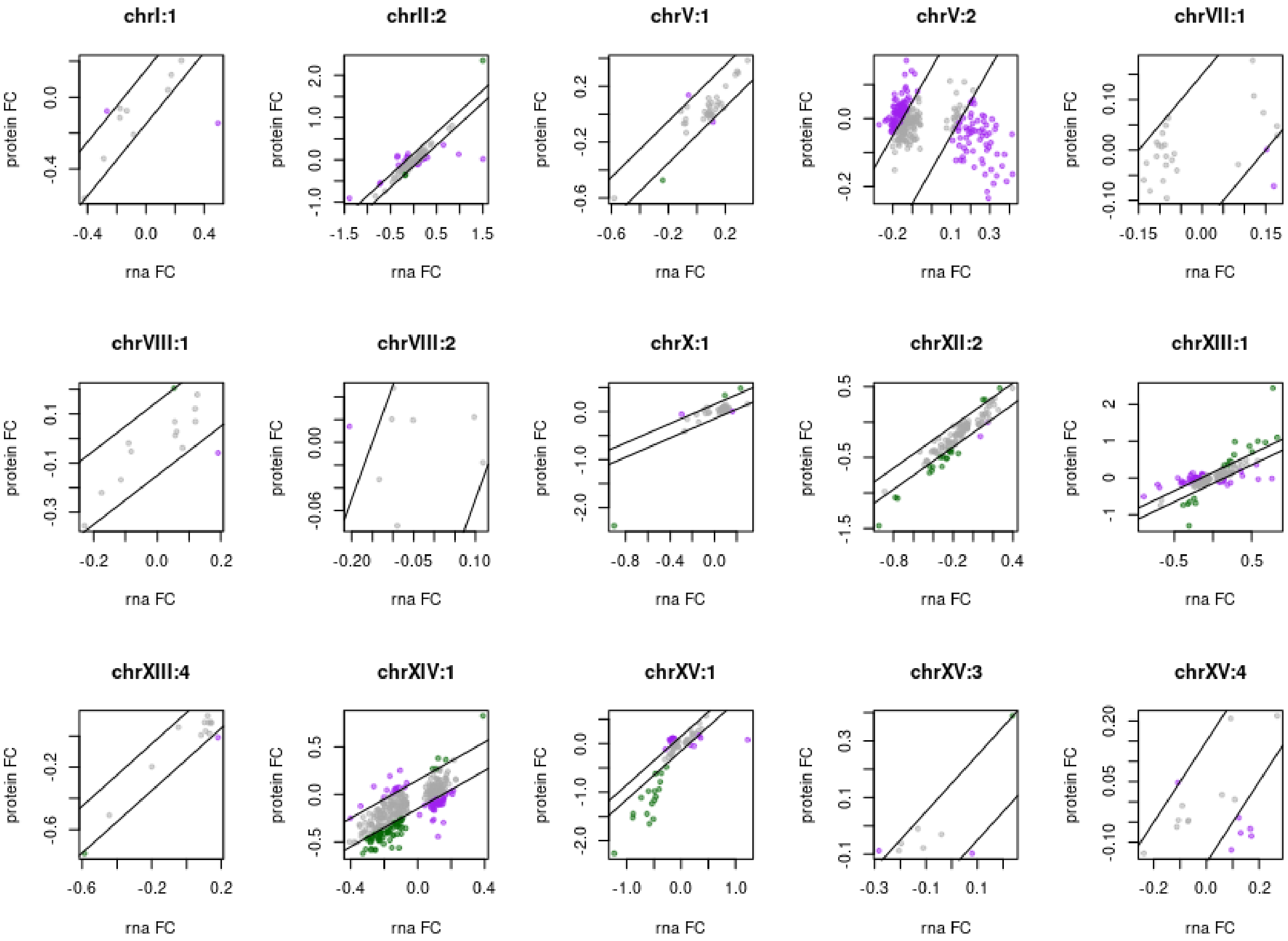

In the statement that QTLs affecting resistance and morphology more likely overlap p and phQTLs than eQTLs and as shown in Figure 5, there are a couple of possible concerns. For example, by using individual transcripts with a pearson correlation when most of the cis-eQTL are in fact nearly bimodal could cause a potential bias against finding these associations. This would require a comparison of the trait distributions in the different classes to ensure that the use of pearson correlation for these tests isn't creating an ascertainment bias for or against a trait class.

First, we would like to point out that the exact statement that QTLs affecting resistance and morphology more likely overlap p and phQTLs than eQTLs is based on Supplementary Table S8 (in the old version)/dataset EV5-S8 (new submission), not on Figure 5. There were did not use Pearson correlation, but analyzed the overlap of QTL loci, as is explained in the Supplementary Table 8's description ("Test of overlap of growth QTL with molecular QTL. Shown are p-values and Odds ratios for one-sided Fisher's exact tests checking whether QTL for compound resistance traits or morphological traits significantly overlap with QTLs for molecular traits from different layers.").

To determine whether trait values for different molecular layers follow qualitatively different distributions, we compared trait distributions of 63 randomly selected genes (among the 402 genes available in all layers) below. In our data, transcripts did not show a bimodal distribution, at least not more than observed in the other molecular layers. We believe that the use of Pearson correlation was appropriate even if there were a bimodal distribution of the molecular trait, Pearson correlation would still be able to capture a linear relationship with physiological traits, if it exists.

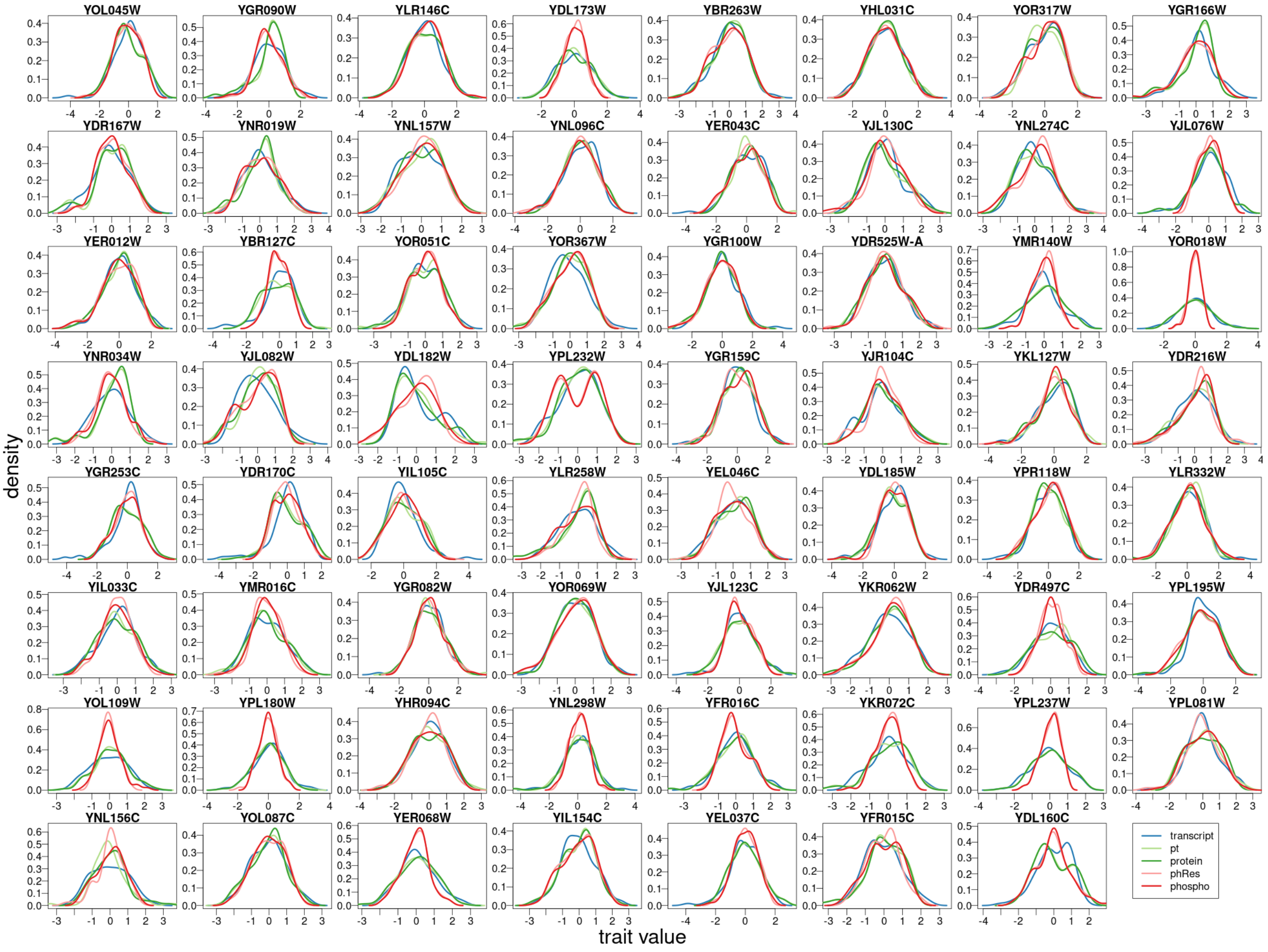

Another note of caution is that this analysis did not appear to include an assessment of the role of hotspots in these correlations but instead treats the traits as a set of unconnected individuals. Often with eQTL hotspots the use of a summary value summing across the network of genes influenced by this locus finds a much higher relationship to any individual physiological phenotype as the network of transcripts controls for stochastic noise in individual transcripts. It would seem that this analysis should really work to account for the influence of the hotspot loci especially when single loci influence large suites of transcripts and proteins in conjunction. This significantly alters the assumption of omics trait independence that seems to be made in this analysis.

Admittedly, Figure 6, then goes into this network level analysis but it would seem that if these causal networks exist as shown in Figure 6 that the analysis in Figure 5 would need to directly assess if these networks create systematic associations that are missed when treating the omics traits as individual values.

First of all, we would like to point out that these analyses were independent of which QTL affect a trait (with exception of the statement "We found that QTLs affecting resistance and morphology were more likely to overlap with p- and phQTLs than with eQTLs" and, indirectly, the mediation analyses, Fig 5d-e). We directly correlated all molecular phenotypes (that were available in all five layers) with physiological phenotypes, irrespective of whether they were targets of hotspots or even a QTL.

For instance, we showed examples of correlation between physiological traits and protein/phospho traits that are hotspots components (Mkt1: MKT1 hotspot, Avt1: GPA1 hotspot), but also examples where there was no evident connection to any hotspot (Rps31, Spe4). Importantly, we did not

“cherry-pick” these examples to include both hotspot and non-hotspot cases, but simply picked them based on how often they were top correlators with physiological traits. Since we saw comparable correlations for both classes (hotspot traits and non-hotspot traits), we can assume that the detection is equally possible for both.

The reviewer’s concern that individual molecular traits are subject to stochastic noise applies to all five layers. Since we treated all layers equally for these analyses, e.g. having transcript and protein abundance of a gene directly “compete” each other for correlation with physiological traits, our conclusions are valid irrespective of the correlation structure between molecular traits.

We do agree with the reviewer that molecular traits operate in networks and we did explore some of them in our work as pointed out by the reviewer. The Reviewer’s suggestion of a "summary value" is very interesting. In essence, this is our hypothesis why we think that protein/phospho traits are more correlated with morphological traits than transcripts. Protein abundance/activity can be seen a summation of all upstream regulations on the transcript layer and therefore to some extent represents the "summary values" that Reviewer 1 suggests. The integration with growth traits can therefore be seen as a validation of our hypothesis that the direction of information flow (usually, with exceptions of course) is RNA -> protein -> phospho -> function (i.e., morphology/growth). We have added the following sentence to the discussion to reflect this idea: “In essence, protein abundance/activity can be seen as a summation of all upstream regulations on the transcript layer, which is why protein/phospho traits are more correlated with physiological traits than transcripts.“

Methods

I'm a bit unsure about the QTL mapping, this appears to be a GWA based analysis approach being applied to a biparental RIL. I understand the use of population structure in a GWAS setting in which there are demographic processes that can bias the genotype sampling and lead to false positives. But in a simple biparental structure, the 129 strains should represent a random sampling of the parental admixture potential with almost no population structure? Is there population structure in this random RIL collection? The concern is that by including a population structure matrix that may not capture any meaningful variation that the random forest model is over structuring the data leading to issues. Have the authors compared the model with and without structure in comparison to a gold standard set of loci to show that structure is not inflating error rates?

The mapping approach used in this work has been partially developed for and used on biparental yeast crosses previously (Michaelson *et al*, 2010; Picotti *et al*, 2013; Clément-Ziza *et al*, 2014). In this earlier work we demonstrated that this approach outperforms conventional mapping approaches in simulated and real data. Based on these prior experiences and the observed overlap between the QTL that we detected and previously published hotspots (Supplementary Table S3 of the old version/dataset EV5-S3 in the new submission) we are confident that the method is generally suitable for QTL-mapping for the present dataset.

Indeed, in a biparental cross we expect much less severe population structure compared to a GWAS in natural populations. Nevertheless, we decided to use a conservative approach and prioritized avoiding false positive associations over potential losses in mapping power. Please note that correcting for population structure tends to reduce the number of detected QTLs. The fact that we see large numbers of QTL for all molecular layers clearly shows that we have sufficient power in this setup despite correcting for population structure. Importantly, if there is very little population

structure, our correction will only have a minor effect on the results, because the kinship matrix will be very homogeneous.

Furthermore, the comparison of our method to HK mapping below (see our response to Reviewer #3) shows a strong qualitative agreement between the scores of our method and those generated with HK, where we did not account for population structure.

In the genotyping section, the manuscript comments that they increased their resolution by using resequencing data to infer segregant genotypes. But the paper should really discuss that the major limitation in resolution is only having 129 genotypes which limits recombination to at best 1cM resolution for maybe a single large effect locus but more likely 5-10 cM for more polygenic traits no matter the genomic sequence.

The Reviewer is of course correct in that the resolution of the QTL mapping is limited by sample size. We added the following sentence in the Results section to make this clear:

'While the resolution of the QTL-mapping is mainly limited by the number of segregants (i.e., recombinations), this procedure allows for more accurate genotyping.'

In the integration of physiological traits, the study says that replicates were merged by taking their simple means. Does this mean that the data was collected by a perfect randomized complete block design? It is only proper to take a simple mean when a RCBD was utilized and there is not blocking structure in the data that could bias the mean. Similarly, are the data normally distributed to justify the use of pearson correlation?

We only merged replicates for the data from Perlstein et al., not for the data from Nogami et al. (Perlstein *et al*, 2007; Nogami *et al*, 2007).The data we took from the Perlstein study was created using 384-well plates containing the segregants, with one such plate for each compound at a specific concentration, including several DMSO controls. The data we downloaded was already corrected by regressing out the growth effects of the control (DMSO) replicates and normalized. Thus, we averaged measurements of multiple plates containing the same compound at the same concentrations only after normalization. Since all segregants were always grown on the same plate, we do not expect that batch effects will have an effect on our analyses, including QTL mapping or correlation.

Data being normally distributed is not a prerequisite for Pearson correlation, as we discussed in a previous comment.

I am a bit puzzled by the definition of a local QTL. The sentence says that if the QTL contains at least one genetic marker with a 0.8 Pearson correlation to a marker in the gene it is considered local. What does this mean in actual units of Linkage. I did a rough back of the envelope calculation and this almost seems to be creating at least a nearly 20cM window around the causal locus to call local. This seems extremely large. This definition of local should be placed in the linkage terminology to allow the reader to interpret the window being used. Also, marker data isn't normally distributed and a pearson correlation would be inappropriate.

The Reviewer raises two points here. 1) They are worried that the window in which we call local QTL might be too large, and 2) that it is based on the Pearson correlation coefficient. As the reviewer notes above, the mapping resolution is limited by sample size. Therefore, some QTL are relatively large and further fine mapping is not feasible. Here we followed the rationale of identifying QTL that are consistent with local variation, i.e. our data does not allow to determine

that the causal marker is *not* local. Using a wider window for defining local QTLs will likely include more QTL that actually have a *trans*-effect on the target. This approach is conservative for our analyses: as shown in Figures 3A, 4C, Supplementary Figure S9/Appendix Fig S10 (old/new submission, respectively), and Supplementary Figure S10/Appendix Fig S11, we do observe significant differences between local and distant QTLs despite a potential 'dilution' with *trans*-effects. As a consequence, we might classify QTL as local whose causal SNP is not necessarily very close in terms of physical base pair distance, but in strong LD with the target gene.

With regards to the concern that the Pearson correlation coefficient is not suitable for computing LD (i.e. applying the coefficient to binary, non-normal data) we want to clarify that in case of binary data, such as genetic markers in a biparental haploid cross, the Pearson correlation coefficient and Spearman's rho are identical. Furthermore, using Pearson correlation is established practice for estimating LD (e.g., (Lin *et al*, 2012)).

Editorial Suggestions

As an editorial comment if the manuscript is meant to appeal to a general audience, it seems somewhat odd to have only yeast and human citations on the application of omics QTL mapping to understand trait variation. This seems to preclude the large body of literature on these topics from Drosophila, C. elegans, Arabidopsis, Maize and Rice, to mention a few. Especially as there are plant papers going from eQTL to enzyme activity in a similar manner as here.

In a similar vein, the statement on page 9 that hotspots are typically transcription factors or kinases is not supported in the plant literature. In the plant literature, these are as often enzymes or other non TF/Kinase genes. This is an example of where a broader reading of the omics literature beyond yeast and humans could contribute to a manuscript with broader appeal.

Thank you for your suggestions on how we can appeal to a larger audience. We agree that there is a rich collection of QTL studies from various species that preclude our work. We have therefore added citations from multiple species to the introduction, as well as edited the Discussion to reflect this:

"Likewise, mass spectrometry has been used to identify QTLs for hundreds of proteins (pQTLs) (Foss *et al*, 2011; Holdt *et al*, 2013; Picotti *et al*, 2013; Wu *et al*, 2013; Fu *et al*, 2009; Singh *et al*, 2016; Okada *et al*, 2016; Keele *et al*, 2021)" and "Our findings also have implications for future work on complex human diseases and *complex traits in other species*."

We have specified in the manuscript that the statement about hotspots often being attributed to master regulators pertains to yeast. We would like to point out that we named transcription factors and kinases only as examples of potential "master regulators". We then state examples of hotspots we observed where the causal genes include a transcription factor (*HAP1)*, a GTPase-activating protein (IRA2), and a component of a translation regulation complex (MKT1). While studies in plants have identified enzymes as such master regulators of hotspots, for example the study by (Fu *et al*, 2009), these studies studied metabolite abundances and metabolically relevant phenotypes. For instance, in that particular study, hotspots thought to be caused by enzymes (e.g. INV, MAM1/2) mainly influenced metabolites and physiological phenotypes, whereas hotspots attributed to transcription factors and kinases (e.g., ER, HUA2/FRL1) also were eQTL or pQTL hotspots. Since we did not quantify metabolites in our study, we did not expect enzymes as causal genes for hotspots.

We have changed the corresponding statement in the manuscript as follows: “Regions in the genome that affect significantly more traits than expected by chance are referred to as QTL hotspots (Smith & Kruglyak, 2008). Since these loci affect large numbers of molecular traits, they often act through master regulators, *for instance* transcription factors or kinases *in yeast* (Albert *et al*, 2018; Yvert *et al*, 2003)“

We wanted to be careful not to over-interpret our data, including speculating about whether the effects we observed could be applicable to other species, including humans, plant species, or other organisms. Although many mechanisms are conserved between species, our data do not allow us to generalize to other organisms. Thus, we have refrained from directly comparing our results to observations from other species.

Reviewer #2:

In this manuscript, Grossbach et al. address an important question: what is the extent and properties of natural genetic variation of the phosphoproteome? They describe an experimental study of systems genetics where three layers of molecular traits are mapped in yeast: transcripts abundance (eQTL), proteins abundance (pQTL) and phosphopeptides abundance (phQTL). In addition, they applied a regression analysis to search for QTLs that account for post-transcriptional control of protein abundance (ptQTL) and for phosphorylation levels relative to protein abundance (phResQTL). To characterize these genetic regulators, the authors compared their locations, their redundancy, their nature and their correlation to physiological traits. They also validated the effects of two linked variants using allele-replacement strains. As expected, the results obtained at the RNA and protein levels are not novel and are totally concordant with previous studies. Results on phosphopeptides are totally novel and have important implications: genetic variation of phosphorylation states per se does exist and it is highly correlated to physiological traits. This should invite colleagues conducting human GWAS to include phosphoproteomics in their designs.

The work is of excellent quality. First, the authors spent considerable efforts to obtain a consistent dataset. The yeast cross they used has previously been extensively studied for mRNA and protein levels. It would have been much easier to use published datasets, and "simply" quantify phosphoproteomes of previously-characterized segregants. Instead, they re-generated all the data themselves, so that all three molecular layers were quantified from the exact same samples, which is important to avoid biases of lab-to-lab and batch-to-batch variability. Second, the analysis of the dataset is thorough, with careful statistical inferences and interpretations of the various molecular effects.

Overall the work is of very high quality and potential impact. I have minor comments that may help the authors improve the manuscript.

We very much thank the reviewer for this encouraging statement.

1. What is the correlation between heritabilities of the different layers? (data of fig2b). It would be interesting to visualize if there are cases (genes) where an elevated heritability is specifically seen for the phosphoproteome layer. Note that this is independent from the QTL results: heritability does not depend on mapping power.

We thank the reviewer for this excellent suggestion! Below we show heritabilities calculated for the same genes at the five layers plotted against each other. For the phospho traits, heritabilities were averaged over peptides belonging to the same protein. In general, heritabilities between layers seem to be independent, except for traits that are correlated anyways (e.g., pt versus protein). This new analysis underlines the fact that genomic variation may affect molecular traits independently at any layer.

Indeed, while we show that transcripts and proteins tend to reach greater heritabilities, there are still genes with greater heritability on the phospho layer. This is in line with the fact that we find QTL for some phospho traits without finding corresponding QTL on the transcript layer.

We have added this plot to the manuscript as Appendix Figure S3.

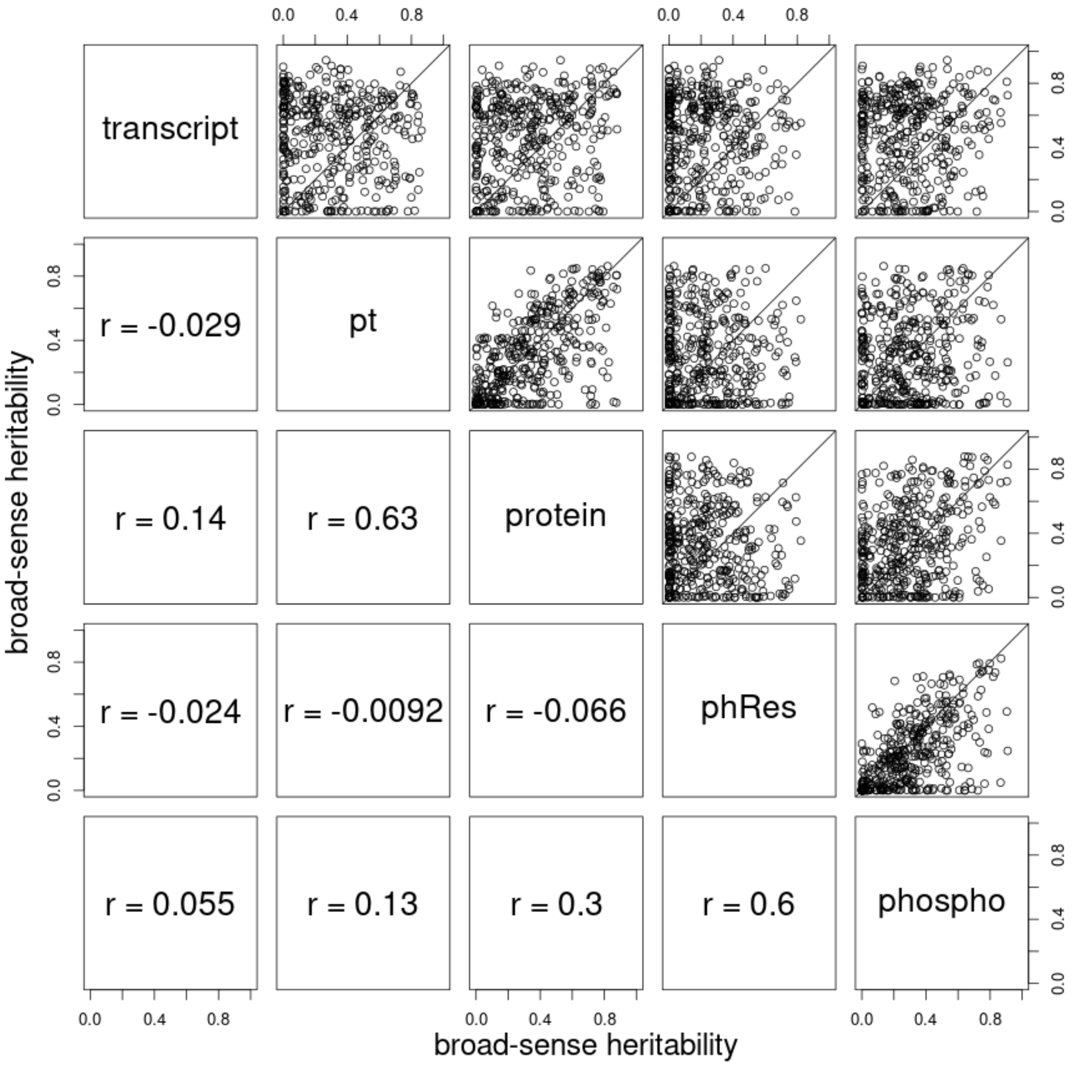

2. Although not needed for this first phosphoQTL study, the discussion could mention possible future experiments that can completely demonstrate the mediation effects of phospho sites (eg. those of fig 5d-e). In principle, it may be possible to combine allele-replacement at the phResQTL with specific amino-acid substitutions on the target protein to evaluate if the genetic effect on the physiological trait is blocked.

Thank you for this suggestion. We have added the following sentence to the discussion:

"Further studies will be needed to fully reveal how phospho traits mediate QTL effects to physiological traits, for example by using amino acid replacements to modify the phospho sites of the mediator and evaluating if the genetic effect on the physiological trait is then blocked."

3. Are the present results on eQTL, pQTL and ptQTL consistent with those of Brion et al. (https://elifesciences.org/articles/60645) who deconvoluted the protein/RNA layers in the same yeast cross?

We compared our results to those of Brion et al. for the six genes that were included in both studies (CTS1, UGP1,CYC1, ARO8, OLE1, GPD1) (Brion *et al*, 2020). In the plot below we show both the association of QTL to the transcripts and proteins of each of these genes as well as the genomic loci identified as QTL for the respective molecular layer by Brion et al. as red bars below.

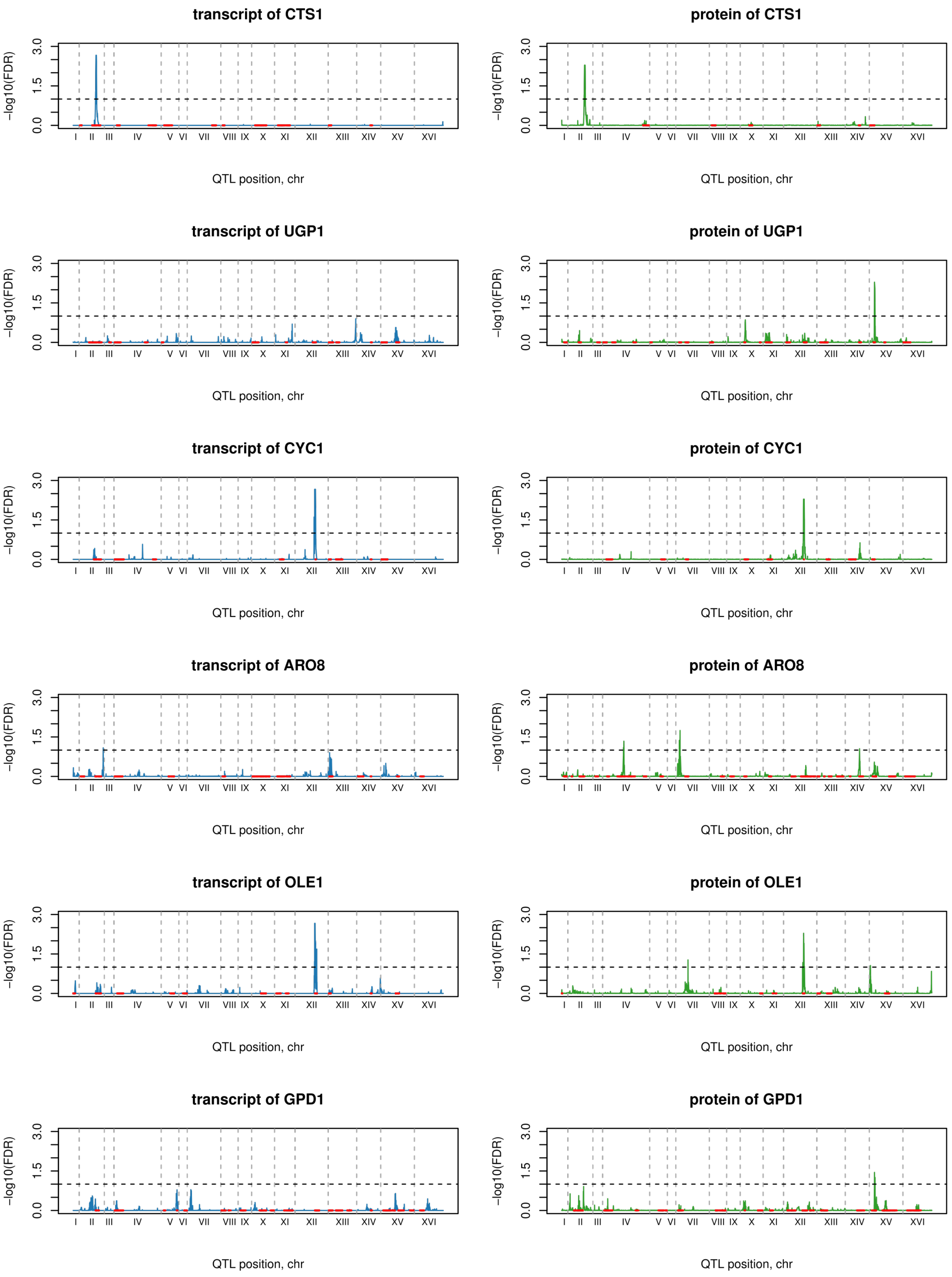

We find limited agreement in QTL between the study by Brion et al. and our data. For example, the HAP1 locus affects both transcript and protein abundance of OLE1 and CYC1 in both studies. Brion et al. further find that the HAP1 locus affects the protein levels of UGP1 and GPD1, which we did not replicate in our study. For CTS1, both studies observed an effect of genomic variation on chrII on the transcript levels but only our study found an accompanying pQTL at the same locus.

Brion et al. observed strong effects of MKT1 on all transcripts they studied and also on a technical control without a guide RNA. Therefore Brion et al. excluded this locus from further analysis. While MKT1 also has a large number of targets on both molecular layers in our study, we do not observe significant effects for the six transcripts studied here. This suggests that differences in the assays used might explain some differences in the observed associations as well.

There are several potential reasons why the results of the two studies differ for these individual genes, most of which can be attributed to technical differences in the study approaches. As Brion et al. state in their article, the trait they study is explicitly not mRNA abundance but production, and some RNA regulation might be altered due to the introduction of an artificial polyA-tail. Some differences might be therefore explained through mechanisms that act on RNA half-life. In addition, while they compared strains with extreme expression (e.g., comparison of strains with high mCherry versus low mCherry), we studied the entire spectrum of variation in expression. Another factor is the fact that they measured single-cell expression while we used bulk data. We expect that transcripts (especially transcript production) are subject to a higher fluctuation in expression over time and thus between individual cells, for example due to transcriptional bursts (Donovan *et al*, 2019). Proteins, on the other hand would be expected to maintain more constant expression levels. This issue is exacerbated by the fact that they selected cells with extreme expression for QTL mapping. On the other hand, their single-cell approach and them measuring RNA and protein abundance in the very same cells might allow them to identify effects that are obscured in our bulk data. Further, the genetic variation studied here differs slightly from that present in the cross studied by Brion et al.. This is evident in the QTL detected for the protein levels of GPD1 on chrX. This QTL was not replicated in our study due to the reason that the parental strains show no variation at this locus. The QTL detected by Brion et al. is, as they discussed, likely caused by mutations within the GFP library strains. While in the case of YAK1 on chrX, this is known, there might be additional variation obscuring the similarities between the results of the two studies.

Despite these differences, the overall conclusions between the studies agree. In both studies, QTL effects can be observed both on the e- and the pQTL layer, but there are also many cases where transcripts and proteins are regulated independently. The main difference are estimates of how prevalent such independent regulation is.

4. Fig6d: Although allele-replacement of STE20 has a moderate effect alone, it has no effect in the context of GPA1-STE20 double replacement. Can the authors discuss this deeper?

We added a statement to the respective section, stating that GPA1 and STE20 have an epistatic relationship:

“The double replacement strain shows changes comparable to those observed in the GPA1 replacement strain, indicating an (alleviating) epistatic relationship between STE20 and GPA1 for the traits considered here.”

- page 18: the IRA2 hotspot is not flagged on Fig3b and we don't know where to look on the Fig panel.
- page 20, typo in legend: "by the a QTL".
- page 21 bottom. Replace "Further" by "(iii)" ?

Thank you for pointing these out, we fixed the manuscript in the respective places.

Reviewer #3:

Grossbach et al present a genetic analysis of transcript, protein and protein phosphorylation in a widely-used yeast panel. They report QTL across all omic-domain and extensively examine and dissect the levels of regulation that give rise to the observed patterns of genetic effects. The impact of genetics on phosphorylation is widespread and is shown to play a significant role in chemical sensitivity and morphological traits that were previously described for the yeast panel. The genetic analysis of phQTL appears to be novel and will undoubtedly spur attention to this important, emerging area of mass-spec proteomics.

The presentation is clear, concise and methods are appropriate and well documented. I have no major concerns.

We very much appreciate this positive evaluation and constructive feedback.

Minor points:

The authors avoided a common statistical error by not taking ratios of ph-peptide to total-peptide abundance. The use of residuals (ph-peptide regressed on total) is an important (positive) feature of the analysis. Although it is unlikely to make a big difference, I wonder why they did not instead directly use the total peptide abundance as a covariate (forced inclusion in the RF models). This would more correct approach - residual regression is a corner cutting approximation with some potential pitfalls (https://doi.org/10.1002/gepi.20607).

The point raised by the reviewer is important in that the relationship of phosphopeptides to their host protein is not trivial. We decided against using the protein level as a predictor in the RF decision trees, because quantitative predictors can be used many times in a single decision tree. Even using forced inclusion in the RF, a single split on the covariate (i.e. peptide abundance) does not correct for it when the covariate is continuous as opposed to categorical (like the genotypes are). Thus, a predictor with a linear relationship with the outcome (in our case the abundance of the phosphopeptide) would have to be split on multiple times in the same tree, which ultimately means that we lose sensitivity to detect influences of binary genetic markers.

While we also model population structure by including continuous predictors in the RF trees this is expected to lead to a much less severe loss of power. This is because the relationship between the population structure and a given phosphopeptide level is expected to be much weaker than that between a phosphopeptide and its host protein. (Especially because there is very little population sub-structure in this cross.)

In addition, there are other technical considerations, for example that it is unknown how including continuous predictors would influence importance estimates for the genetic predictors. Permutations might no longer be comparable between traits anymore, because the Forests include different sets of predictors, leading to a vastly increased runtime for QTL mapping. In short, by including protein levels as a covariate we would force RF to learn the relationship between phospho-peptides and protein levels from scratch for each trait individually, which likely leads to a loss of power and/or overfitting issues.

As stated in the paper referenced by the Reviewer, the two-stage approach that we used mainly introduces a bias towards the null, i.e. a loss of power, but not false positives (Demissie & Cupples, 2011). Thus, we took a more conservative approach by modeling the relationship between phosphopeptides and corresponding proteins directly at the cost of losing QTL detection power. This might partly explain why we detect slightly fewer QTL for the intermediate layers (pt and phRes) than for the separate layers.

The RF QTL mapping method appeared in 2010 but has not been widely adopted. Could this choice be further justified? Do the authors expect non-additive QTL effects? Could the RF QTL be compared to a standard method (e.g. HK regression) or compared to previously reported eQTL and pQTL to confirm that similar results are being obtained? If new QTL are emerging from the RF analysis, a comment and closer look at why would be helpful.

The QTL mapping method used in this study was validated on comparable data where it outperformed other methods including HK (Michaelson *et al*, 2010). Further it has been used by other groups for similar purposes (Francesconi & Lehner, 2014; Heyn *et al*, 2014). Our Random Forest-based approach especially excels in cases where there are non-additive effects between markers (Stephan *et al*, 2015). Epistasis was previously shown to play a role in yeast QTL, although estimates of the proportion of epistasis on total trait heritability vary (Bloom *et al*, 2013, 2015; Costanzo *et al*, 2016).

In order to ensure that our method for mapping QTL does not lead to artifacts which could bias the downstream analysis, we computed LOD-Scores using HK regression for all traits in the transcriptome. A comparison of the scores computed by RFQTL and HK as implemented through the 'scanone' function of the R-package 'qtl' is shown below. Note that we did not account for population structure in the HK regression.

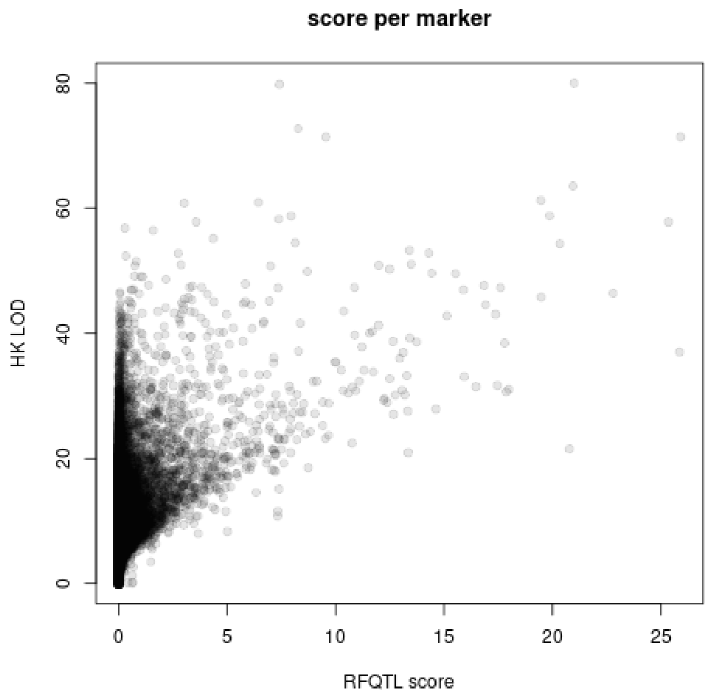

While our method does not generate high scores for associations with low LODs, we occasionally observe the reverse, i.e. associations with high LODs but low RFQTL-scores. These are due to more narrow peaks around causal loci in the RFQTL method as exemplified by the transcript of YDR264C:

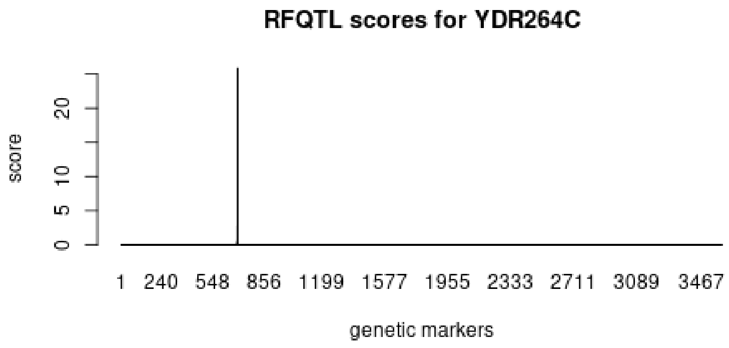

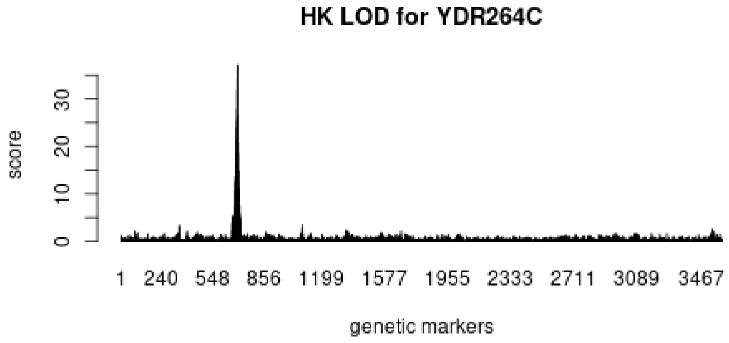

When only considering the maximum score per chromosome, no major differences in the results of the two methods remain:

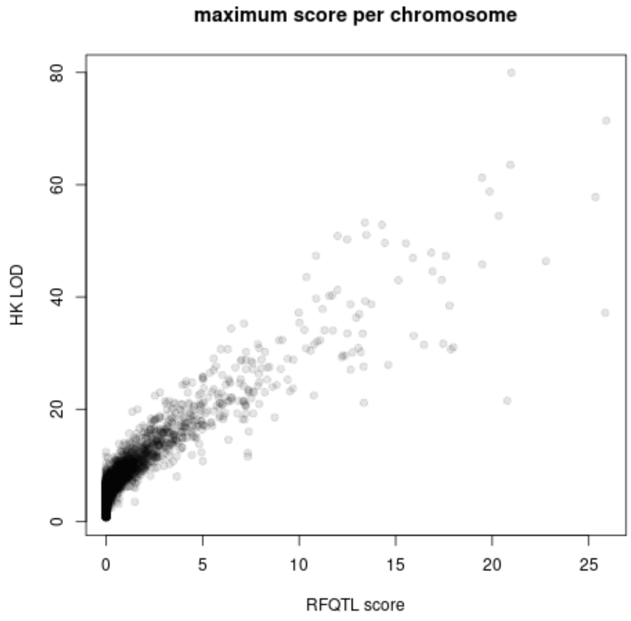

We are therefore confident that our choice in RFQTL as mapping method does not bias our results in a meaningful way. Further, we detect previously known hotspots for eQTL and pQTL (Supplementary Table S3/dataset EV5-S3 in the old/new submission, respectively).

Please add a comment in the main text to indicate that polymorphic peptides were removed from analysis. I found this deep in the Methods but fretted about it until I got there.

To make this important point more clear we added this sentence to the results section:
"Only peptides that were not polymorphic between the parental strains were considered here."

References

Albert FW, Bloom JS, Siegel J, Day L & Kruglyak L (2018) Genetics of trans-regulatory variation in gene expression. *eLife* 7

Bloom JS, Ehrenreich IM, Loo WT, Lite T-LV & Kruglyak L (2013) Finding the sources of missing heritability in a yeast cross. *Nature* 494: 234–237

Bloom JS, Kotenko I, Sadhu MJ, Treusch S, Albert FW & Kruglyak L (2015) Genetic interactions contribute less than additive effects to quantitative trait variation in yeast. *Nat Commun* 6: 8712

Brion C, Lutz SM & Albert FW (2020) Simultaneous quantification of mRNA and protein in single cells reveals post-transcriptional effects of genetic variation. *eLife* 9: e60645

Clément-Ziza M, Marsellach FX, Codlin S, Papadakis MA, Reinhardt S, Rodríguez-López M, Martin S, Marguerat S, Schmidt A, Lee E, *et al* (2014) Natural genetic variation impacts expression levels of coding, non-coding, and antisense transcripts in fission yeast. *Molecular Systems Biology* 10: 764

Costanzo M, VanderSluis B, Koch EN, Baryshnikova A, Pons C, Tan G, Wang W, Usaj M, Hanchard J, Lee SD, *et al* (2016) A global genetic interaction network maps a wiring diagram of cellular function. *Science*

Demissie S & Cupples LA (2011) Bias due to two-stage residual-outcome regression analysis in genetic association studies. *Genetic Epidemiology* 35: 592–596

Donovan BT, Huynh A, Ball DA, Patel HP, Poirier MG, Larson DR, Ferguson ML & Lenstra TL (2019) Live-cell imaging reveals the interplay between transcription factors, nucleosomes, and bursting. *EMBO J* 38: e100809

Foss EJ, Radulovic D, Shaffer SA, Goodlett DR, Kruglyak L & Bedalov A (2011) Genetic variation shapes protein networks mainly through non-transcriptional mechanisms. *PLoS biology* 9: e1001144

Francesconi M & Lehner B (2014) The effects of genetic variation on gene expression dynamics during development. *Nature* 505: 208–211

Fu J, Keurentjes JJB, Bouwmeester H, America T, Verstappen FWA, Ward JL, Beale MH, de Vos RCH, Dijkstra M, Scheltema RA, *et al* (2009) System-wide molecular evidence for phenotypic buffering in Arabidopsis. *Nat Genet* 41: 166–167

Heyn H, Sayols S, Moutinho C, Vidal E, Sanchez-Mut JV, Stefansson OA, Nadal E, Moran S, Eyfjord JE, Gonzalez-Suarez E, *et al* (2014) Linkage of DNA Methylation Quantitative Trait Loci to Human Cancer Risk. *Cell Reports* 7: 331–338

Holdt LM, von Delft A, Nicolaou A, Baumann S, Kostrzewa M, Thiery J & Teupser D (2013) Quantitative trait loci mapping of the mouse plasma proteome (pQTL). *Genetics* 193: 601–608

Keele GR, Zhang T, Pham DT, Vincent M, Bell TA, Hock P, Shaw GD, Paulo JA, Munger SC, Pardo-Manuel de Villena F, *et al* (2021) Regulation of protein abundance in genetically diverse mouse populations. *Cell Genomics* 1: 100003

Lin C-Y, Xing G & Xing C (2012) Measuring linkage disequilibrium by the partial correlation coefficient. *Heredity* 109: 401–402

Michaelson JJ, Alberts R, Schughart K & Beyer A (2010) Data-driven assessment of eQTL mapping methods. *BMC genomics* 11: 502

Nogami S, Ohya Y & Yvert G (2007) Genetic Complexity and Quantitative Trait Loci Mapping of Yeast Morphological Traits. *PLOS Genetics* 3: e31

Okada H, Ebhardt HA, Vonesch SC, Aebersold R & Hafen E (2016) Proteome-wide association studies identify biochemical modules associated with a wing-size phenotype in Drosophila melanogaster. *Nat Commun* 7: 12649

Perlstein EO, Ruderfer DM, Roberts DC, Schreiber SL & Kruglyak L (2007) Genetic basis of individual differences in the response to small-molecule drugs in yeast. *Nature Genetics* 39: 496–502

Picotti P, Clément-Ziza M, Lam H, Campbell DS, Schmidt A, Deutsch EW, Röst H, Sun Z, Rinner O, Reiter L, *et al* (2013) A complete mass-spectrometric map of the yeast proteome applied to quantitative trait analysis. *Nature* 494: 266–270

Singh KD, Roschitzki B, Snoek LB, Grossmann J, Zheng X, Elvin M, Kamkina P, Schrimpf SP, Poulin GB, Kammenga JE, *et al* (2016) Natural Genetic Variation Influences Protein Abundances in C. elegans Developmental Signalling Pathways. *PLOS ONE* 11: e0149418

Smith EN & Kruglyak L (2008) Gene-environment interaction in yeast gene expression. *PLoS biology* 6: e83

Stephan J, Stegle O & Beyer A (2015) A random forest approach to capture genetic effects in the presence of population structure. *Nat Commun* 6: 7432

Wu L, Candille SI, Choi Y, Xie D, Jiang L, Li-Pook-Than J, Tang H & Snyder M (2013) Variation and genetic control of protein abundance in humans. *Nature* 499: 79–82

Yvert G, Brem RB, Whittle J, Akey JM, Foss E, Smith EN, Mackelprang R & Kruglyak L (2003) Trans-acting regulatory variation in Saccharomyces cerevisiae and the role of transcription factors. *Nature Genetics* 35: 57–64

9th Mar 2022

RE: MSB-2021-10712R, The impact of genomic variation on protein phosphorylation states and regulatory networks

Thank you for sending us your revised manuscript. We have now heard back from the two reviewers who were asked to evaluate your revised study. As you will see below, the reviewers are satisfied with the modifications made and are overall supportive of publication. Reviewer #1 still requests some clarifications. We would ask you to address their comments in a minor revision. We would also ask you to address some minor editorial issues listed below.

REFEREE REPORTS

Reviewer #1:

The authors have responded to the majority of my comments. I only have a few points that need clarification within the manuscript.

It should be pointed out that in most quantitative genetics labs that I interact with that Haley Knot QTL mapping is no longer considered appropriate or publishable. This is because Haley Knot has a long known issue with ghost peaks wherein linkage with actual QTLs can create artificial signals of significance. This was the impetus towards composite interval, multiple interval and even more recent Bayesian QTL approaches that work to take this linkage information into account. Both the HK and GWAS/QTL approach in this paper don't account for this. As such, while comparison to HK is helpful, it doesn't provide a accurate comparison to more acceptable QTL approaches for linkage populations like this yeast population.

I agree that data normality is not a pre-requisite to using Pearson correlation. I was simply suggesting that the bimodality of eQTL and other aspects can create false positive and negative relationships. Based on personal experience with this type of data, spearman rank is a more cautious approach to conducting correlation analysis across phenotypic levels. The authors should at least discuss their choice and how this may or may not influence the analysis and interpretation.

For the window size question about using 0.8 Pearson correlation, I should qualify. I was not concerned about the use of pearson correlation but was simply saying that this was a non-standard descriptor of genetic window size. Usually window sizes are presented in cM or basepairs in the majority of literature on quantitative genetics. I was simply saying that I, and likely other readers, are able to place a 0.8 pearson correlation value into a comparative framework to interpret the window size. I was solely asking for the authors to provide a translation of 0.8 Pearson correlation into cM or basepairs to allow an ascertainment if the window is conservative or liberal.

Reviewer #3:

I thank the authors for address the points raised in my review. I have no further concerns or comments.

Comments of Reviewer 1

The authors have responded to the majority of my comments. I only have a few points that need clarification within the manuscript.

It should be pointed out that in most quantitative genetics labs that I interact with that Haley Knot QTL mapping is no longer considered appropriate or publishable. This is because Haley Knot has a long known issue with ghost peaks wherein linkage with actual QTLs can create artificial signals of significance. This was the impetus towards composite interval, multiple interval and even more recent Bayesian QTL approaches that work to take this linkage information into account. Both the HK and GWAS/QTL approach in this paper don't account for this. As such, while comparison to HK is helpful, it doesn't provide a accurate comparison to more acceptable QTL approaches for linkage populations like this yeast population.

We do agree with the reviewer that HK is not state-of-the-art and we have therefore used a different Bayesian approach for comparison (see below). However, we wish to correct the statement that the "GWAS/QTL approach in this paper" would not account for linkage information. The opposite is true: it is one of the major advantages of the Random Forest-based approach that it does consider all markers simultaneously. As a consequence it has much greater power identifying informative markers and it 'suppresses' markers in LD with an already selected marker. Within a decision tree genetic markers are considered in consecutive nodes, thereby conditioning the trait data on previously used markers. A marker that is very similar (in high LD) to another one previously used in the same tree will explain little information in the preconditioned data and hence will not be selected. This leads to narrower Peaks than those produced with single-marker methods, as shown in the comparison to HK in the previous revision. Please, see (Stephan *et al*, 2015) for more information on the multi-marker nature of the RF-based QTL mapping and a direct comparison to other multi-marker mapping methods.

To demonstrate that the QTL-mapping approach used in this work is comparable to other state-of-the-art methods, we mapped eQTL with a Bayesian method implemented in the iBMQ R-package (Imholte *et al*, 2013), building individual models for each trait. Iterations were set to 50,000 and the burn in was set to 25000.
Below we show a comparison of the posterior probabilities of genetic markers that either overlap with a QTL found with our approach or do not. Each point of data within one of the boxes indicates the strength of association between one trait and one genetic marker.

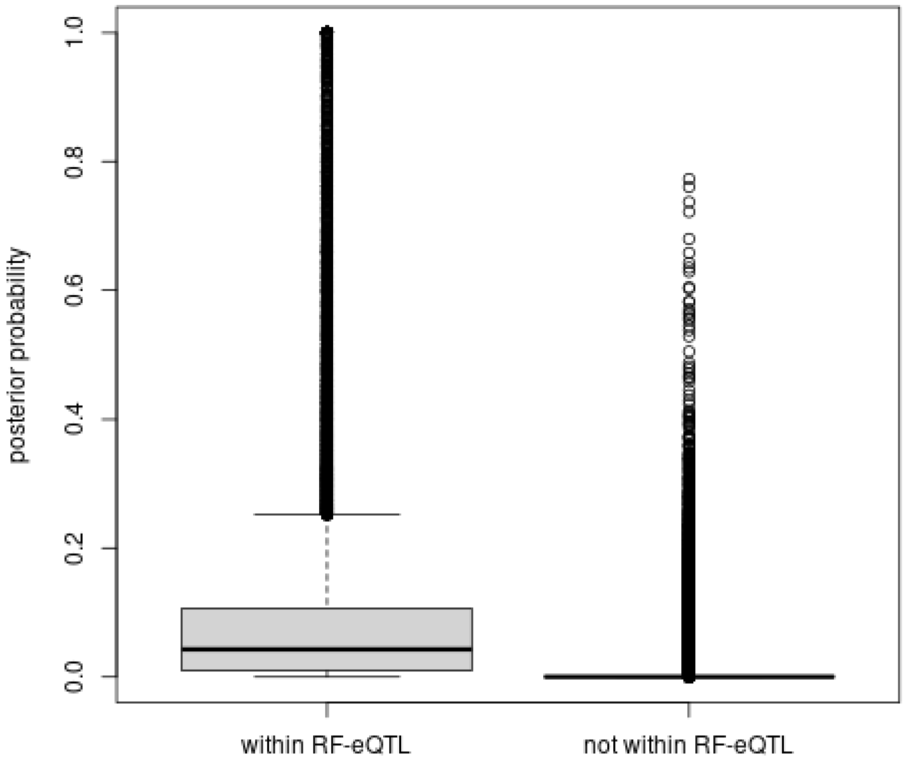

Markers that overlap with QTL detected by the Random Forest (RF)-based method for a given trait have on average greater posterior probabilities than those markers that do not overlap with RF-based QTL ($p<2.2*10^{-16}$).

Next, we chose a threshold for calling QTL with the Bayesian approach that results in a similar total number of eQTL as the RF approach (a PPA cutoff of 0.07 results in 5680 eQTL, vs. 5776 eQTL at 10% FDR with the RF approach). Here we also observed a striking overlap: 73% of eQTL called with the Bayesian approach were also discovered with the RF approach, and 75% of eQTL called with the RF approach were also discovered with the Bayesian approach. Also, the number of expression traits associated with each locus were highly similar between the two methods:

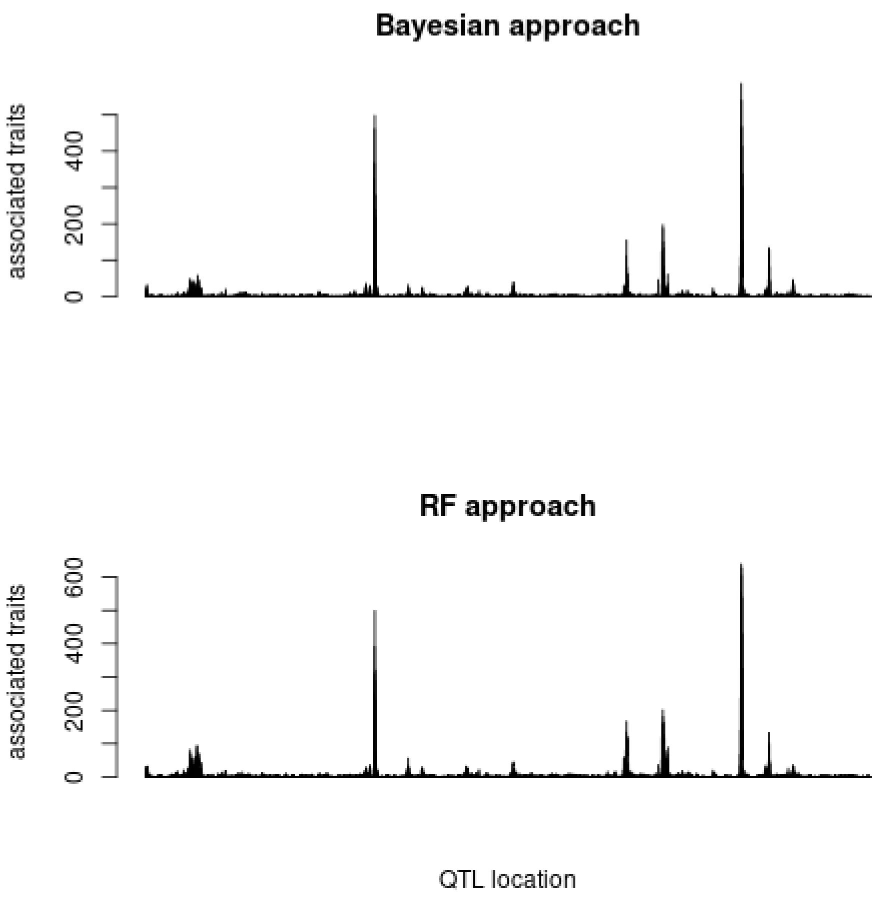

Based on the direct comparison and conceptual considerations we feel confident that our chosen approach for QTL-mapping is at least as suitable for this work as the Bayesian approach tested above.

I agree that data normality is not a pre-requisite to using Pearson correlation. I was simply suggesting that the bimodality of eQTL and other aspects can create false positive and negative relationships. Based on personal experience with this type of data, spearman rank is a more cautious approach to conducting correlation analysis across phenotypic levels. The authors should at least discuss their choice and how this may or may not influence the analysis and interpretation.

We thank the reviewer for their constructive suggestion. We agree that the Spearman rank correlation is a more conservative approach, whereas the Pearson's correlation has greater statistical power, provided the requirements are met. In order to examine if the choice of the correlation measure influences our conclusions, we have conducted the following analyses. To examine if the use of Pearson's r leads to an artificial increase or decrease of transcripts that strongly correlate with a growth trait as shown in **Figure 5**, we additionally computed Spearman's rho for each pair of growth and molecular trait. Below we show the strongest correlators of growth traits by their rho (Panels **a** and **b**). This is equivalent to **Figures 5a** and **b**, but computed with Spearman's approach. In Panels **c** and **d** we present a direct comparison

of the correlation values for the strongest correlators based on Pearson's r (which are shown in the original **5a** and **b**).

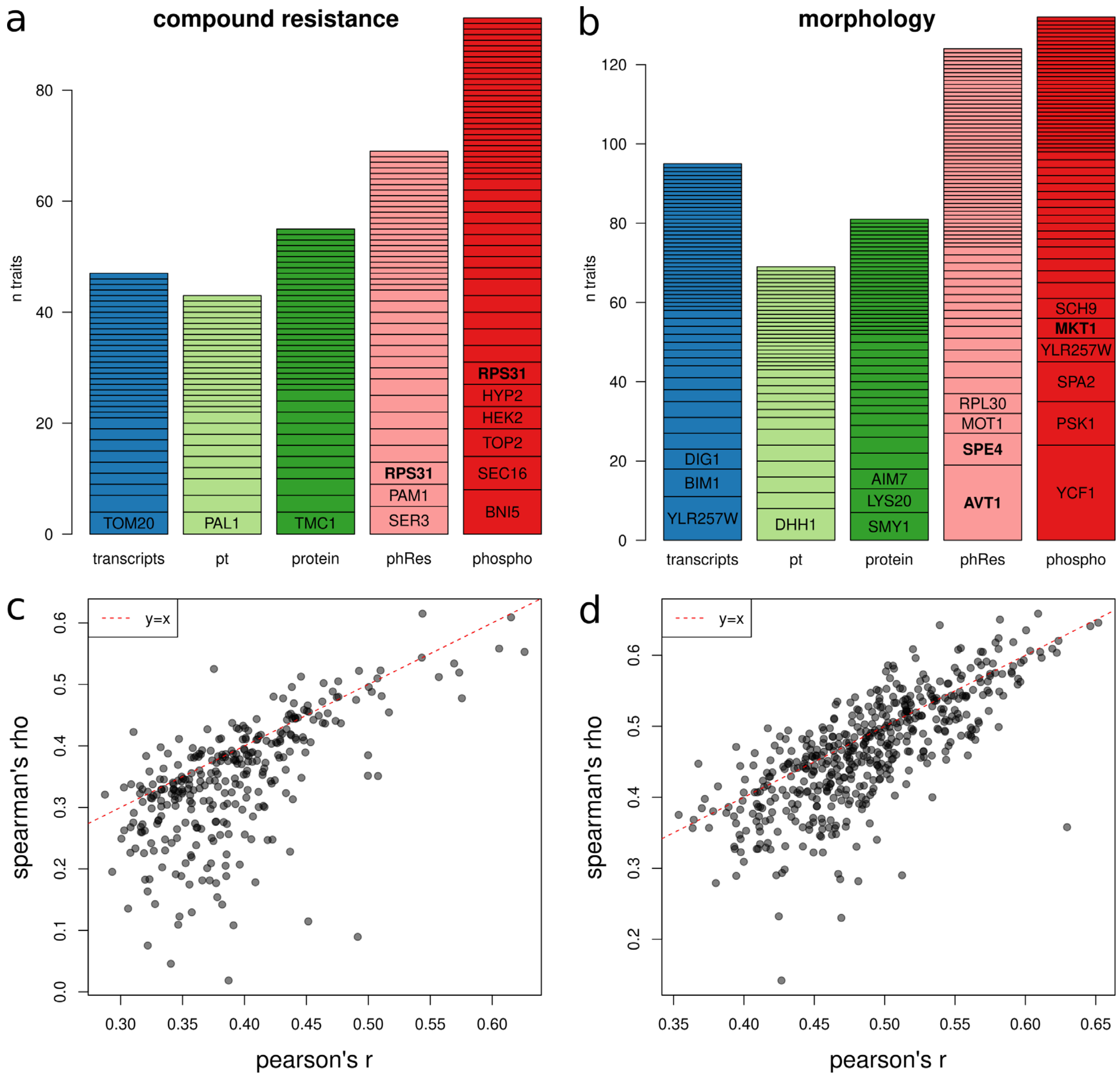

Comparing Panels a and b from above to the original Figure 5 in the manuscript shows that the choice of the correlation measure barely affects the results. The main conclusion that the phospho-traits are on average more predictive of cellular traits than the RNA- and protein abundance traits is not affected. When the top correlators are chosen based on Spearman's rho, we see an over-representation of phosphotraits. This was also observed when choosing the top correlators based on Pearson's r. The top correlators based on Pearson's r show rho values of similar magnitude (as shown in Panels **c** and **d**). This demonstrates that the use of Pearson's r and Spearman's rho leads to very similar results in this application. While some traits show lower correlation coefficients using Spearman's rho, this is not surprising since the traits were selected based on maximum Pearson's correlation coefficients.

This observation is consistent with the fact that in our data RNA expression values are distributed very similarly to other molecular traits, as shown in the first revision.

For the window size question about using 0.8 Pearson correlation, I should qualify. I was not concerned about the use of pearson correlation but was simply saying that this was a non-standard descriptor of genetic window size. Usually window sizes are presented in cM or basepairs in the majority of literature on quantitative genetics. I was simply saying that I, and likely other readers, are able to place a 0.8 pearson correlation value into a comparative framework to interpret the window size. I was solely asking for the authors to provide a translation of 0.8 Pearson correlation into cM or basepairs to allow an ascertainment if the window is conservative or liberal.

As the reviewer notes, providing the cM distance corresponding (on average) to a correlation of r>=0.8 allows the reader to put this information into context of other literature on the topic. We have therefore added this information when we first define the window of local linkage in the results section:
"QTL were considered local if they had a correlation of r>0.8 with the allele of the affected gene. Marker pairs fulfilling this criterion had a genetic linkage of 7.9 cM on average."

We also added a paragraph in the methods section explaining how we computed the linkage in cM.

Reviewer #3:

I thank the authors for address the points raised in my review. I have no further concerns or comments.

We thank the reviewer for the positive feedback.

References:

Imholte GC, Scott-Boyer M-P, Labbe A, Deschepper CF & Gottardo R (2013) iBMQ: a R/Bioconductor package for integrated Bayesian modeling of eQTL data. *Bioinformatics* 29: 2797–2798

Stephan J, Stegle O & Beyer A (2015) A random forest approach to capture genetic effects in the presence of population structure. *Nat Commun* 6: 7432

ACCEPTED **12th Apr 2022**

Thank you again for sending us your revised manuscript. We are now satisfied with the modifications made and I am pleased to inform you that your paper has been accepted for publication.

EMBO PRESS

YOU MUST COMPLETE ALL CELLS WITH A PINK BACKGROUND ➔

PLEASE NOTE THAT THIS CHECKLIST WILL BE PUBLISHED ALONGSIDE YOUR PAPER

Corresponding Author Name: Ruedi Aebersold, Andreas Beyer

Journal Submitted to: Molecular Systems Biology

Manuscript Number: MSB-2021-10712

Reporting Checklist For Life Sciences Articles (Rev. June 2017)

This checklist is used to ensure good reporting standards and to improve the reproducibility of published results. These guidelines are consistent with the Principles and Guidelines for Reporting Preclinical Research issued by the NIH in 2014. Please follow the journal's authorship guidelines in preparing your manuscript.

A- Figures
1. Data
The data shown in figures should satisfy the following conditions:

- ➔ the data were obtained and processed according to the field's best practice and are presented to reflect the results of the experiments in an accurate and unbiased manner.
- ➔ figure panels include only data points, measurements or observations that can be compared to each other in a scientifically meaningful way.
- ➔ graphs include clearly labeled error bars for independent experiments and sample sizes. Unless justified, error bars should not be shown for technical replicates.
- ➔ if n< 5, the individual data points from each experiment should be plotted and any statistical test employed should be justified
- ➔ Source Data should be included to report the data underlying graphs. Please follow the guidelines set out in the author ship guidelines on Data Presentation.

2. Captions
Each figure caption should contain the following information, for each panel where they are relevant:

- ➔ a specification of the experimental system investigated (eg cell line, species name).
- ➔ the assay(s) and method(s) used to carry out the reported observations and measurements
- ➔ an explicit mention of the biological and chemical entity(ies) that are being measured.
- ➔ an explicit mention of the biological and chemical entity(ies) that are altered/varied/perturbed in a controlled manner.

- ➔ the exact sample size (n) for each experimental group/condition, given as a number, not a range;
- ➔ a description of the sample collection allowing the reader to understand whether the samples represent technical or biological replicates (including how many animals, litters, cultures, etc.).
- ➔ a statement of how many times the experiment shown was independently replicated in the laboratory.
- ➔ definitions of statistical methods and measures:
  - common tests, such as t-test (please specify whether paired vs. unpaired), simple χ^2 tests, Wilcoxon and Mann-Whitney tests, can be unambiguously identified by name only, but more complex techniques should be described in the methods section;
  - are tests one-sided or two-sided?
  - are there adjustments for multiple comparisons?
  - exact statistical test results, e.g., P values = x but not P values < x;
  - definition of 'center values' as median or average;
  - definition of error bars as s.d. or s.e.m.

Any descriptions too long for the figure legend should be included in the methods section and/or with the source data.

In the pink boxes below, please ensure that the answers to the following questions are reported in the manuscript itself. Every question should be answered. If the question is not relevant to your research, please write NA (non applicable). We encourage you to include a specific subsection in the methods section for statistics, reagents, animal models and human subjects.

USEFUL LINKS FOR COMPLETING THIS FORM

http://www.antibodypedia.com
http://1degreebio.org
http://www.equator-network.org/reporting-guidelines/improving-bioscience-research-report
http://grants.nih.gov/grants/olaw/olaw.htm
http://www.mrc.ac.uk/Ourresearch/Ethicsresearchguidance/Useofanimals/index.htm
http://ClinicalTrials.gov
http://www.consort-statement.org
http://www.consort-statement.org/checklists/view/32-consort/66-title
http://www.equator-network.org/reporting-guidelines/reporting-recommendations-for-tumo
http://datadryad.org
http://figshare.com
http://www.ncbi.nlm.nih.gov/gap
http://www.ebi.ac.uk/ega
http://biomodels.net/
http://biomodels.net/miriam/
http://jjj.biochem.sun.ac.za
https://osp.od.nih.gov/biosafety-biosecurity-and-emerging-biotechnology/
http://www.selectagents.gov/

B- Statistics and general methods

Please fill out these boxes ➔ (Do not worry if you cannot see all your text once you press return)

1.a. How was the sample size chosen to ensure adequate power to detect a pre-specified effect size?	We used an existing cross which was previously shown to be adequately sized for QTL mapping.
1.b. For animal studies, include a statement about sample size estimate even if no statistical methods were used.	NA
2. Describe inclusion/exclusion criteria if samples or animals were excluded from the analysis. Were the criteria pre-established?	NA
3. Were any steps taken to minimize the effects of subjective bias when allocating animals/samples to treatment (e.g. randomization procedure)? If yes, please describe.	NA
For animal studies, include a statement about randomization even if no randomization was used.	NA
4.a. Were any steps taken to minimize the effects of subjective bias during group allocation or/and when assessing results (e.g. blinding of the investigator)? If yes please describe.	NA
4.b. For animal studies, include a statement about blinding even if no blinding was done	NA
5. For every figure, are statistical tests justified as appropriate?	Yes.
Do the data meet the assumptions of the tests (e.g., normal distribution)? Describe any methods used to assess it.	We used appropriate non-parametric tests where necessary, e.g. Wilcoxon rank sum test instead of Student's t-test when data did not follow a normal distribution.
Is there an estimate of variation within each group of data?	Yes.

Is the variance similar between the groups that are being statistically compared?	Yes.

C- Reagents

6. To show that antibodies were profiled for use in the system under study (assay and species), provide a citation, catalog number and/or clone number, supplementary information or reference to an antibody validation profile. e.g., Antibodypedia (see link list at top right), 1DegreeBio (see link list at top right).	NA
7. Identify the source of cell lines and report if they were recently authenticated (e.g., by STR profiling) and tested for mycoplasma contamination.	NA

* for all hyperlinks, please see the table at the top right of the document

D- Animal Models

8. Report species, strain, gender, age of animals and genetic modification status where applicable. Please detail housing and husbandry conditions and the source of animals.	All yeast strains used are described in Table 1 and Dataset EV1. Culture conditions are descibed in the Material and Methods section.
9. For experiments involving live vertebrates, include a statement of compliance with ethical regulations and identify the committee(s) approving the experiments.	NA
10. We recommend consulting the ARRIVE guidelines (see link list at top right) (PLoS Biol. 8(6), e1000412, 2010) to ensure that other relevant aspects of animal studies are adequately reported. See author guidelines, under 'Reporting Guidelines'. See also: NIH (see link list at top right) and MRC (see link list at top right) recommendations. Please confirm compliance.	NA

E- Human Subjects

11. Identify the committee(s) approving the study protocol.	NA
12. Include a statement confirming that informed consent was obtained from all subjects and that the experiments conformed to the principles set out in the WMA Declaration of Helsinki and the Department of Health and Human Services Belmont Report.	NA
13. For publication of patient photos, include a statement confirming that consent to publish was obtained.	NA
14. Report any restrictions on the availability (and/or on the use) of human data or samples.	NA
15. Report the clinical trial registration number (at ClinicalTrials.gov or equivalent), where applicable.	NA
16. For phase II and III randomized controlled trials, please refer to the CONSORT flow diagram (see link list at top right) and submit the CONSORT checklist (see link list at top right) with your submission. See author guidelines, under 'Reporting Guidelines'. Please confirm you have submitted this list.	NA
17. For tumor marker prognostic studies, we recommend that you follow the REMARK reporting guidelines (see link list at top right). See author guidelines, under 'Reporting Guidelines'. Please confirm you have followed these guidelines.	NA

F- Data Accessibility

18: Provide a "Data Availability" section at the end of the Materials & Methods, listing the accession codes for data generated in this study and deposited in a public database (e.g. RNA-Seq data: Gene Expression Omnibus GSE39462, Proteomics data: PRIDE PXD000208 etc.) Please refer to our author guidelines for 'Data Deposition'.<br><br>Data deposition in a public repository is mandatory for:<br>a. Protein, DNA and RNA sequences<br>b. Macromolecular structures<br>c. Crystallographic data for small molecules<br>d. Functional genomics data<br>e. Proteomics and molecular interactions	The datasets and computer code produced in this study are available in the following databases:RNA-seq reads: ArrayExpress (E-MTAB-8146, https://www.ebi.ac.uk/arrayexpress/experiments/E-MTAB-8146/)<br>MS data: Pride (PXD010893, http://proteomecentral.proteomexchange.org/cgi/GetDataset?ID=PXD010893)<br>Analysis scripts: GitHub (https://github.com/Jan88/phosphoQTL)
19. Deposition is strongly recommended for any datasets that are central and integral to the study; please consider the journal's data policy. If no structured public repository exists for a given data type, we encourage the provision of datasets in the manuscript as a Supplementary Document (see author guidelines under 'Expanded View' or in unstructured repositories such as Dryad (see link list at top right) or Figshare (see link list at top right).	All data produced in this study will be made publically available upon publication. Raw data is accessible using the links given in the "Data Availability" section, processed data is available as Expanded View Datasets and Tables.
20. Access to human clinical and genomic datasets should be provided with as few restrictions as possible while respecting ethical obligations to the patients and relevant medical and legal issues. If practically possible and compatible with the individual consent agreement used in the study, such data should be deposited in one of the major public access-controlled repositories such as dbGAP (see link list at top right) or EGA (see link list at top right).	NA
21. Computational models that are central and integral to a study should be shared without restrictions and provided in a machine-readable form. The relevant accession numbers or links should be provided. When possible, standardized format (SBML, CellML) should be used instead of scripts (e.g. MATLAB). Authors are strongly encouraged to follow the MIRIAM guidelines (see link list at top right) and deposit their model in a public database such as Biomodels (see link list at top right) or JWS Online (see link list at top right). If computer source code is provided with the paper, it should be deposited in a public repository or included in supplementary information.	All computations were done using R. Analysis scripts are available and necessary R packages are publically accessible.

G- Dual use research of concern

22. Could your study fall under dual use research restrictions? Please check biosecurity documents (see link list at top right) and list of select agents and toxins (APHIS/CDC) (see link list at top right). According to our biosecurity guidelines, provide a statement only if it could.	NA
